# Supplementary material for: Rapid Assay for Sick Children with Acute Lung infection Study (RASCALS): diagnostic cohort study protocol
Source: BMJ Open. 2021 Nov 29;11(11):e056197. doi: 10.1136/bmjopen-2021-056197 (PMC8634010; doi:10.1136/bmjopen-2021-056197)
Supplement: Supplementary data [file bmjopen-2021-056197supp002.pdf]

Department of Paediatric Intensive Care

Dr Roddy O'Donnell
Dr Rosalie Campbell
Dr David Inwald
Dr Shruti Agrawal
Dr Nazima Pathan
Dr Girish Neelegowda
Dr Riaz Kayani

Cambridge University Hospitals NHS
NHS Foundation Trust

Addenbrooke's Hospital
Hills Road
Cambridge CB2 0QQ

Switchboard: 01223 245151
www.addenbrookes.org.uk

Rapid Assay for Sick Children with Acute Lung infection Study
Invitation and Information Sheet – Parent/Carer of critically ill child

RASCALS
Rapid Assay for Sick Children with Acute Lung infection Study

Thank you for taking the time to consider having your child participate in 'RASCALS' – the Rapid Assay for Sick Children with Acute Lung infection Study.

Before you decide whether to have your child participate it is important for you to understand why the research is being done and what it will involve. Please take time to read the following information carefully and discuss it with others if you wish. Do ask us if there is anything that is not clear or if you would like more information. Take time to decide whether or not you wish to take part.

Key points about the study

- We are investigating bacteria and viruses that cause chest infection in children. In this study we will be using your child's breathing tube secretions, swabs, blood and faeces to help us improve the way we diagnose these infections.
- All the children in the study will be tested for Coronavirus 2019 (COVID-19), we will let you know the results.
- One of the rapid bacteria and virus tests is something we're trying for the first time. We will be doing this test using what is left over from your child's breathing tube sample which is needed even if they are not in the study. We will share the results of this test with you, and your child's doctors. It is possible these test results could help your child's doctors make decisions regarding your child's antibiotic treatment. We will ask them via a survey if the test was helpful.
- If your child is strongly suspected to have COVID-19 we will ask for your help collecting weekly throat swabs and either a swab of the bottom or faecal sample. This will continue for 4 weeks and will conclude with a survey about your child's symptoms.
- If at any point you no longer wish for your child to take part let your nurses and doctors know. Your child will receive high quality care during their stay in the paediatric intensive care unit whether or not they take part.

Key points about your child's information

Clark JA, *et al. BMJ Open* 2021; 11:e056197. doi: 10.1136/bmjopen-2021-056197

- In this research study we will use information from your child's medical records. We will only use information that we need for the research study. We will let very few people know your name or contact details, and only if they really need it for this study.
- Everyone involved in this study will keep your data safe and secure. We will also follow all privacy rules. At the end of the study we will save some of the data in case we need to check it and for future research.
- We will make sure no-one can work out who you are from the reports we write. The following information pack tells you more about this.

Kind regards,
The Paediatric Intensive Care Unit Research Team

RASCALS researchers: Dr Nazima Pathan, Dr John Clark, Dr Iain Kean, Dr Estée Török, Prof Gordon Dougan, Prof Stephen Baker, Dr Vilas Navapurkar, Ms Esther Daubney & Ms Deborah White.
Phone: 01223 336883
Email: np409@medschl.cam.ac.uk

*This study has been co-sponsored by Cambridge University Hospitals NHS Foundation Trust and the University of Cambridge. Any reference to 'we' or 'us' in these documents refers to these entities.

Clark JA, *et al. BMJ Open* 2021; 11:e056197. doi: 10.1136/bmjopen-2021-056197

Carer Information: Rapid Assay for Sick Children with Acute Lung infection Study

What is the purpose of this study?
The Department of Paediatric Intensive Care at Addenbrooke's Hospital is actively involved in research in children who are critically ill. We want to do research that helps us develop better ways of diagnosing and treating children admitted to our unit. Our research nurses and doctors would like to talk to you about the work that we are doing.

In this specific study, we are interested in COVID-19 and in the diagnosis and treatment of lung infections. Currently, the best tests we have available for diagnosing lung infections are slow, and often can't detect the bugs that have caused infection. We are trying a new rapid infection test to see if it might be faster and able to detect these bugs more often. We will share the results of the test with you and your child's doctors. We will ask your doctors whether the test had any impact on their decisions on the type and duration of antibiotics they prescribe. Your child will still have all their routine tests done as well.

In addition to the rapid infection test, we will be using the samples we collect to test for new markers of infection and COVID-19.

Why has my child been chosen?
We are studying children that are unwell in the Paediatric Intensive Care Unit that have potential COVID-19 and/or lung infection requiring intubation and ventilation (a breathing tube down the windpipe and the support of a breathing machine). We we are aiming to improve tests and obtain more information about COVID-19.

Who is organising the study?
The study is being organised by the Department of Paediatric Intensive Care by Dr Nazima Pathan and Dr John Clark, here at Addenbrooke's Hospital. This work is supervised by the University of Cambridge. No specific payments will be made to any of the staff involved for including your child in this study other than their normal salary.

Does my child have to take part?
It is up to you whether or not your child should take part. If you chose for your child to participate you will be given this information sheet to keep and be asked to sign a consent form. If you decide to have your child take part, you are still free to withdraw this decision at any time and without giving a reason. A decision to withdraw or a decision not to take part will not affect the standard of care your child will receive.

What will happen to my child if they take part?
Your child will receive infection tests whilst we can care for them in intensive care, and the 4 weeks that follow if they are strongly suspected to have COVID-19. The first set of tests will be done by hospital staff. If your child is well enough to go home in the following 4 weeks we will ask for your help taking throat swabs and faecal samples or swabs of the bottom. We will provide directions on how to do this and how to post the samples back to us. A chart of the tests that will be done is on the following page.

When	What test?		What for?	Will I find out the result?
As soon as possible	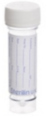	Breathing tube sample	Bacteria and viruses	Yes
			Coronavirus 2019	Yes
			Markers of infection and antibiotic resistance	No – this will be for research only
	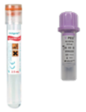	Blood	Markers of infection	No – this will be for research only
			Coronavirus 2019	Yes
	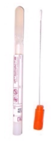	Nasopharyngeal swab (swab at the back of the nose) or nasopharyngeal aspirate (secretions from back of the nose)	Coronavirus 2019	Yes
	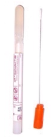	Throat swab	Coronavirus 2019	Yes
	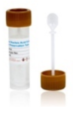	Faecal sample or rectal swab	Coronavirus 2019	Yes*
			Markers of infection and antibiotic resistance	No – this will be for research only
If there is strong clinical suspicion or tests confirm Coronavirus 2019, the following samples will be obtained.				
2-4 days later	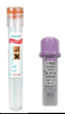	Blood	Markers of infection and antibiotic resistance	No – this will be for research only
			Coronavirus 2019	Yes*
5-7 days later	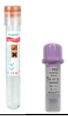	Blood	Markers of infection and antibiotic resistance	No – this will be for research only
			Coronavirus 2019	Yes*
Weekly for 4 weeks	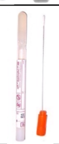	Throat swab	Coronavirus 2019	Yes*
	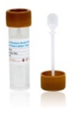	Faecal sample or rectal swab	Coronavirus 2019	Yes*
			Markers of infection and antibiotic resistance	No – this will be for research only

* The first set of nose and throat swabs we do will be processed quickly, however COVID-19 tests on other samples may take multiple weeks given resources available in the health service.

None of the samples we obtain will result in extra procedures for your child. The blood sample amount will be within safe limits according to the weight of your child, which we will take from their central line. We will not need to use any extra needles to take this blood.

The rapid bacteria and virus test we are trying has not been used before in sick children, therefore it will be in addition to our normal infection tests. We will let you and your child's doctors know the results of this new test. We will ask the doctors whether this test made any difference to the antibiotic treatment they prescribe. This will be via a short questionnaire we have developed.

All the samples we take will be frozen once we have finished our study and stored for up to 10 years. This means that with ethics committee approval they could be used for other related studies.

Why have you saved samples prior to asking me?
To best detect infection tests need to be done as early as possible. The tests don't work as well once more and more doses of antibiotics are given. We put some of the samples we need to take aside so this study would not mean we were doing extra procedures on your child. Whether we use these samples for our study is your decision.

What do I and my child have to do?
No specific restrictions need to be placed upon your child as a consequence of participating in the study. Your child would continue with any regular medication.

Aside from taking and posting the samples mentioned earlier, we will ask you to complete a short online survey. We will send you a link to this survey via your email address. It will contain questions about your child's symptoms and how long they lasted, in addition to whether your child was given any antibiotics once they left the intensive care unit. This data will go directly to a secure online database only accessible by the research team.

What are the possible risks / side effects of taking part?
Taking extra blood and secretion samples will not mean any significant extra risk or harm to your child. Taking the secretion samples from the breathing tube does not cause additional harm to your child's lungs.

What are the possible benefits of taking part?
The study is aimed at improving our ability to diagnose the cause of lung infections and identifying the best treatment early.

Being part of this study means additional information will be obtained regarding possible causes of infection in your child. It is not known whether the results will have any benefits / implications for your child directly. However, by taking part you may contribute to helping children and adults suffering from chest infections in the future.

The COVID-19 tests may help you decide how long you keep your child and your family isolated. We will discuss the results of the tests with your child's medical team to provide you with the best information about what you should do.

What if new information becomes available?
If, during the course of this study, new information becomes available suggesting that in order to give your child the best possible care for your child's condition and treatment should be changed, then we will make those changes. Changes such as these will not affect your child's participation in this study.

What happens at the end of the study?
Any samples that your child has provided up until the end of the study will be stored by the research team and pending ethical approval may be used in another project. If we do not make a subsequent application for ethical approval in the required timeframe or if such an application is refused, then your child's samples will be destroyed.

How will we use information about your child?
We will need to use information from your child's medical records for this research project. This information will include your child's name, medical record number and date of birth. People will use this information to do the research or to check your records to make sure that the research is being done properly. This information containing identifiers will be retained for up to 15 years.

People who do not need to know who your child is will not be able to see these personal details. Your child's data will have a code number instead. We will keep all information about your child safe and secure.

Once we have finished the study, we will keep some of the data so we can check the results. We will write our reports in a way that no-one can work out that you took part in the study.

At the end of the study the data will become "open data". This means that it will be stored in an online database so that it is publicly available. This data will be thoroughly anonymised by removing any personal information that could identify your child, such as names and addresses, before submission. This process is integral to the research process as it allows other researchers to verify results and avoid duplicating research. Data are made available on a website, free of charge, to anyone interested in the research, or who wishes to conduct their own analysis of the data. We would therefore have no control over how these data are used.

Will my child's GP be informed?
With your agreement, your child's GP will be informed should you consent to their participation in the study.

What are your choices about how your information is used?

- You can stop your child being part of the study at any time, without giving a reason, but we will keep information about your child that we already have.
- We need to manage your child's records in specific ways for the research to be reliable. This means that we won't be able to let you see or change the data we hold about your child.
- If you agree for your child to take part in this study, you will have the option to take part in future research using your data saved from this study. This data will be kept within the University of Cambridge Department of Paediatrics for up to 15 years following the conclusion of the study.

Where can you find out more about how your child's information is used?
You can find out more about how we use your information

- at www.hra.nhs.uk/information-about-patients/
- our leaflet available from **www.hra.nhs.uk/patientdataandresearch**
- by asking one of the research team

- by sending an email to np409@medschl.cam.ac.uk or
- by ringing us on 01223 336883

What if there is a problem?
If, when taking part in the project, your child is harmed as a consequence of either the management or conduct of the project then the NHS indemnity scheme will apply. If your child is harmed as a consequence of a problem with the design of the project, then this will be covered by insurance through the University of Cambridge's Clinical Trials policy. If your child is harmed due to someone's negligence, then you may have grounds for a legal action, but you may have to pay for it. Regardless of this, if you wish to complain, or have any concerns about any aspect of the way you have been approached or treated during the course of this study, you can contact the National Health Service Patient Advice and Liaison Service (PALS) or the Independent Complaints Advocacy Service (ICAS). Contact details are as follows:

Patient Advice and Liaison Service (PALS)
Box 53, Cambridge University Hospitals NHS Foundation Trust,
Addenbrooke's Hospital, Hills Road,
Cambridge, CB2 0QQ
Tel: 01223 216756, Fax: 01223 256170

Cambridgeshire, Norfolk and Suffolk ICAS
POhWER,
PO Box 14043
Birmingham
B6 9BL
Tel: 0300 456 2370, Fax: 0300 456 2365

What if we want to withdraw from the study?
You may withdraw your child from the study at any time without giving a reason. A decision to withdraw will not affect the standard of care you receive. In line with the guidelines from the UK Medical Research Council on the use of biological samples in scientific research, we will treat each of the blood samples which you provide us as a gift / donation. Consequently, even if you decide to withdraw from the study we may (with your permission) continue to retain any / all of the samples which you may have already provided but will discard them if you prefer.
If you wish to withdraw from the study, please let your bedside nurse know and they will get in touch with one of the research nurses or doctors.

Who has reviewed the study?
All research in the NHS is looked at by independent group of people, called a Research Ethics Committee, to protect your interests. This study has been reviewed by the research advisory committee of NHS Cambridge University Hospitals NHS Foundation Trust and the research ethics committee of Bradford-Leeds.

Thank you for considering your child's participation in this research project.

Clark JA, *et al. BMJ Open* 2021; 11:e056197. doi: 10.1136/bmjopen-2021-056197
