# Supplementary material for: Rapid Assay for Sick Children with Acute Lung infection Study (RASCALS): diagnostic cohort study protocol
Source: BMJ Open. 2021 Nov 29;11(11):e056197. doi: 10.1136/bmjopen-2021-056197 (PMC8634010; doi:10.1136/bmjopen-2021-056197)
Supplement: Supplementary data [file bmjopen-2021-056197supp003.pdf]

Department of Paediatric Intensive Care

Dr Roddy O'Donnell
Dr Rosalie Campbell
Dr David Inwald
Dr Shruti Agrawal
Dr Nazima Pathan
Dr Girish Neelegowda
Dr Riaz Kayani

Cambridge University Hospitals NHS
NHS Foundation Trust

Addenbrooke's Hospital
Hills Road
Cambridge CB2 0QQ

Switchboard: 01223 245151
www.addenbrookes.org.uk

Rapid Assay for Sick Children with Acute Lung infection Study
Consent Form – Parent/Carer of critically ill child

Thank you for taking the time to consider having your child participate in 'RASCALS' – the Rapid Assay for Sick Children with Acute Lung infection Study and reading the information sheets.

If you would like your child to take part in the study, please read and sign the following forms.

We will provide you with one copy of this form and retain a second copy securely in our research department at the hospital.

Kind regards,

The Paediatric Intensive Care Unit Research Team
RASCALS researchers: Dr Nazima Pathan, Dr John Clark, Dr Iain Kean, Dr Estée Török, Prof Gordon Dougan, Prof Stephen Baker, Dr Vilas Navapurkar, Ms Esther Daubney & Ms Deborah White.
Phone: 01223 336883
Email: np409@medschl.cam.ac.uk

Clark JA, *et al. BMJ Open* 2021; 11:e056197. doi: 10.1136/bmjopen-2021-056197

**Patient identification number**:		**Initials**
1.	I have read the information sheet on this project, dated ___/___/____ (Version________) and have been given a copy to keep. I have been able to ask questions about the project and I understand why the research is being done and any risks involved.	
2.	I give my permission for a breathing tube secretion sample, blood sample, swabs and faecal samples to be used for the purposes of this study. I understand that this blood sample did not result in any additional needle procedures for my child for the purposes of the study.	
3.	I understand that I may be asked to assist with obtaining swabs and stool specimens from my child if they are well enough to go home in the next 4 weeks if they have strongly suspected or confirmed Coronavirus disease 2019.	
4.	I understand how the samples will be collected and that giving samples for this research is voluntary. I understand I can withdraw consent for my child's participation in the study at any time, without giving a reason and without my child's medical treatment or legal rights being affected.	
5.	I understand that the samples taken could be used for other research studies, if approved by an ethics committee, for up to 10 years after the study.	
6.	I understand that the research team will email me a survey to complete via a secure online platform in 4 weeks to ask about my child's symptoms, and treatment.	
7.	I give permission for someone from the research team to look at my child's medical records to get information on my child's illness and related medical history where it is relevant to the research.	
8.	I know how to contact the research team if I need to, and how to get information about the results of the research.	
9.	I understand that any information collected about my child as part of this study will be stored securely in line with current NHS and University guidelines. I understand that only members of the clinical project team will have access to my child's identifiable information; all others analysing my data will only have access to anonymised data. I understand that my child's data will be retained for 15 years.	
10.	I understand that the information collected about my child will be used to support other research in the future, and may be shared anonymously with other academic and commercial researchers external to the project within the UK and beyond	
11.	I consent to my child taking part in this study	

Clark JA, *et al. BMJ Open* 2021; 11:e056197. doi: 10.1136/bmjopen-2021-056197

Written consent

Carer

Name (Capitals):____

Signature:____________________________ Date: dd/mm/yyyy

Relationship to patient: ____________________________

Person obtaining consent

Name (Capitals):____

Signature:____________________________ Date: dd/mm/yyyy

Role: ____________________________

Electronic consent

Carer name (Capitals):____

Person obtaining consent

Name (Capitals):____

Signature:____________________________ Date: dd/mm/yyyy

Role: ____________________________

Verbal consent

Carer name (Capitals):____

Person obtaining consent

Name (Capitals):____

Signature:____________________________ Date: dd/mm/yyyy

Role: ____________________________

Witness to verbal consent

Name (Capitals):____

Signature:____________________________ Date: dd/mm/yyyy

Role: ____________________________

Primary investigator countersign for electronic and unwitnessed verbal consent

Dr Nazima Pathan ____________________________ Date: dd/mm/yyyy

Clark JA, *et al. BMJ Open* 2021; 11:e056197. doi: 10.1136/bmjopen-2021-056197
