# Supplementary material for: Rapid Assay for Sick Children with Acute Lung infection Study (RASCALS): diagnostic cohort study protocol
Source: BMJ Open. 2021 Nov 29;11(11):e056197. doi: 10.1136/bmjopen-2021-056197 (PMC8634010; doi:10.1136/bmjopen-2021-056197)
Supplement: Supplementary data [file bmjopen-2021-056197supp001.pdf]

Appendices

Appendix A: Patient information sheet

Appendix B: Patient consent form

Appendix C: NB-BAL sampling procedure

Appendix D: NB-BAL sampling procedure modifications for COVID-19

Appendix E: Consultant survey

Appendix F: Staff focus group information sheet

Appendix G: Staff focus group consent form
