# Supplementary material for: Rapid Assay for Sick Children with Acute Lung infection Study (RASCALS): diagnostic cohort study protocol
Source: BMJ Open. 2021 Nov 29;11(11):e056197. doi: 10.1136/bmjopen-2021-056197 (PMC8634010; doi:10.1136/bmjopen-2021-056197)
Supplement: Supplementary data [file bmjopen-2021-056197supp004.pdf]

Paediatric intensive care unit (PICU)

Division E

Policy

Non-bronchoscopic bronchoalveolar lavage sampling in children

Key messages

- Non-bronchoscopic bronchoalveolar lavage (NB-BAL) can be used to obtain lower respiratory tract secretions to assist in the diagnosis and treatment of lower respiratory tract infection
- NB-BAL samples have a higher yield than endotracheal samples alone
- NB-BAL sampling should only be undertaken after discussion with the PICU consultant that the patient is safe for the procedure
- The procedure should only be undertaken when a senior PICU nurse (in addition to the bedside nurse) and PICU doctor are available on the unit to assist if required

1 Scope

Paediatric intensive care unit – Addenbrooke's Hospital

2 Purpose

This document is to assist staff in understanding the procedure of NB-BAL sampling, and standardise the way samples are being obtained via NB-BAL in the PICU

3 Definitions

ETT: Endotracheal tube
NB-BAL: Non-bronchoscopic bronchoalveolar lavage
PICU: Paediatric intensive care unit

4 Introduction

Non-bronchosopic bronchoalveolar lavage (NB-BAL) may be also referred to as 'blind' bronchoalveolar lavage. It is a method of sampling secretions found in the lower respiratory tract, for children that are requiring invasive ventilation in intensive care for suspected or confirmed infection. Infection may be suspected based on clinical findings or chest x-ray changes.

NB-BAL has a sensitivity of up to 50-84% and specificity up to 50-95%, for detecting microorganisms in ventilator associated pneumonia.[1,2,3] There is higher

Clark JA, *et al. BMJ Open* 2021; 11:e056197. doi: 10.1136/bmjopen-2021-056197

yield than endotracheal tube (ETT) sampling alone.[1] Advantages include reduced cost in comparison to bronchoscopic bronchoalveolar lavage, less expertise required and the ability to undertake the procedure on smaller children that may not have an airway large enough for bronchoscopy sampling to be undertaken.

Samples of lower respiratory tract secretions can assist in the management of children by way of identifying pathogens causing underlying lower respiratory tract infection. This may then allow antimicrobial therapy to be directed towards the causative bacteria, or ceased if the illness has no underlying bacterial aetiology.

Complications are uncommon in children,[1] and can be prevented or minimised with careful prior assessment of patients.

5 Responsibilities

- PICU consultant – advise whether patient is safe to undergo NB-BAL
- Senior PICU nurse and PICU doctor – maintain availability within the PICU whilst patient is undergoing NB-BAL, in case additional intervention is required
- NB-BAL proceduralist (physiotherapist/nurse/PICU doctor) – undergo procedure as per policy

6 Procedure

6.1 Indications

1. To obtain specimens for microbiology and virology that can assist in identifying pathogens causing lower respiratory tract infection

6.2 Contraindications

- FiO2 >0.85[4]
- PEEP >10cm H_2O[4]
- Pneumothorax
- Raised intracranial pressure
- Acute pulmonary oedema
- Active seizures
- Severe acute asthma
- Thrombocytopaenia[4] with platelets <50 x 10^9 L^{-1}

6.3 Precautions (discuss with PICU consultant)

- Cardiovascular instability

Clark JA, *et al. BMJ Open* 2021; 11:e056197. doi: 10.1136/bmjopen-2021-056197

- Deranged clotting
- Pulmonary hypertension
- Pulmonary haemorrhage
- High ventilation pressures required

6.4 Complications

- Hypoxia[145]
- Transient reduction in tidal volume[4]
- Vagal stimulation[5]
- Pneumothorax
- Mucosal trauma
- Increased intracranial pressure
- Haemodynamic instability[5]
- Arrhythmia[3]
- Bronchospasm[3]
- Haemoptysis[6]
- Haemorrhage

6.5 Pre-procedure preparation

This procedure should only be performed where senior nursing staff and a member of medical staff are present in the PICU. These staff should be notified prior to the procedure taking place.

Consider whether additional sedation will be required for the child to facilitate the procedure. This should be discussed with nursing and medical staff.

Review the most recent chest x-ray - note the position of the tip of the ETT and distance from the carina. The suction catheter will be advanced to the level of the carina i.e. depth of ETT + distance from tip of ETT to carina

6.6 Equipment required

- T-piece anaesthetic circuit
- Bag valve mask available
- Alternative suction device eg. Yankauer suction tip available
- End-tidal CO_2, oxygen saturation and heart rate monitoring, blood pressure monitoring
- Dressing pack with sterile gloves
- Sterile suction catheter (Bicakcilar, suction catheter ideal tip, REF 190 1085 1)
- 2x Tube suction connector with female Luer-lock port (Vycon REF 801.00)
- 1x Male:Male Luer-lock connector (Vycon REF 893.00)
- Sputum trap
- 10mL, 5mL or 2mL syringes (dependent on patient size)
- 3 way tap
- 0.9% sodium chloride at room temperature

Clark JA, *et al. BMJ Open* 2021; 11:e056197. doi: 10.1136/bmjopen-2021-056197

Paediatric intensive care unit (PICU)

Division E

6.7 NB-BAL procedure

6.7.1 Preparation

1. Discuss procedure with legal guardian/care giver where possible, and the child if requiring minimal sedation.
2. Inform nursing and medical staff plan to undertake the procedure and discuss whether additional sedation is required
3. Wash hands and put on face shield, gloves and apron, in addition to any patient specific infection control attire. Assemble equipment as shown in image 1.
4. Draw up 1ml/kg of 0.9% sodium chloride, to a maximum of 10mL in a syringe, leaving an additional 2mL air in the syringe. Connect to 3 way tap and prime suction catheter.
5. Ensure 3 way tap is open to saline flush but closed to suction system
6. Connect suction circuit, with appropriate suction pressure for age of child
7. Review observations and ventilation settings, and confirm with nursing staff safety to proceed with sedation (if indicated) and NB-BAL
8. Pre-oxygenate for 2 minutes[4] (unless there is a cardiac contraindication)
9. Auscultate lung fields

Image 1: Assembled equipment for non-bronchoscopic bronchoalveolar lavage

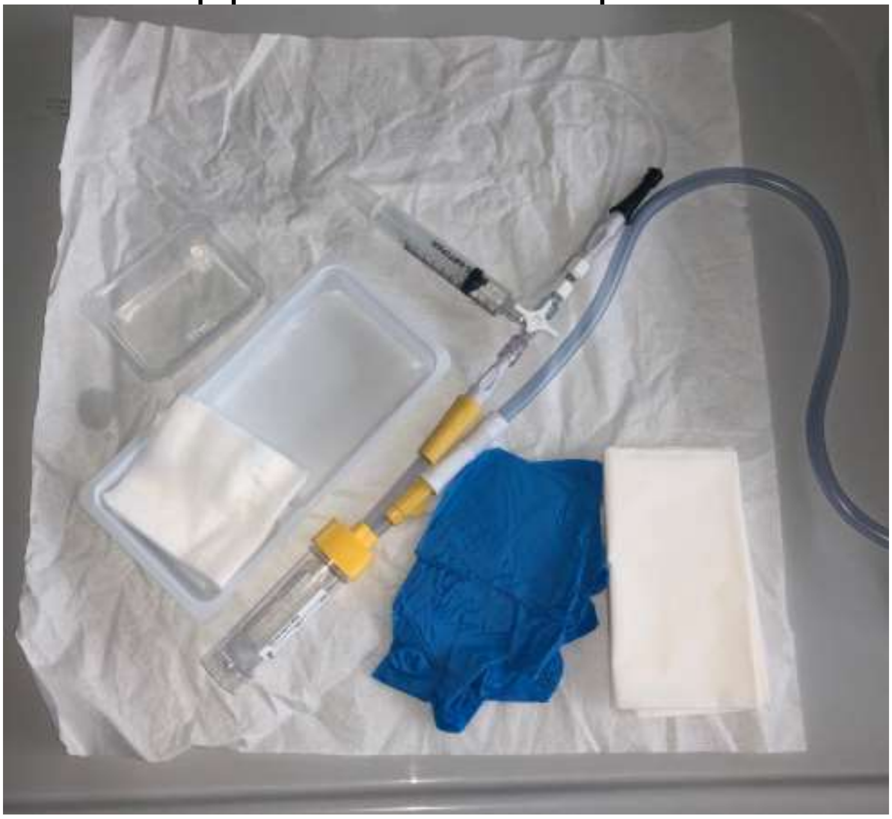

Clark JA, *et al. BMJ Open* 2021; 11:e056197. doi: 10.1136/bmjopen-2021-056197

6.7.2 Procedure

1. Request nursing team to administer sedation agent if this is required
2. Position child. If there is unilateral lung pathology, position the head to the contra-lateral side. i.e. If there is right side lung consolidation, position the head to the left.
3. Provide manual inflations via t-piece
4. Advance the suction catheter to the level of the carina
5. Instill saline and the 2mL of air contained in the syringe. Volume instilled is at the discretion of the proceduralist, as this may vary depending on quality of the secretions but should not exceed volume described in 'preparation'.
6. Open 3 way tap to suction, delivering suction for 2-4 seconds, withdrawing the catheter by maximum 2cm. Ensure sputum trap remains in the upright position.[7]
7. Close 3 way tap to suction and withdraw catheter

6.7.3 Post-procedure

1. Reconnect to mechanical ventilation
2. Label and send sputum sample for microbiology, cultures and sensitivities

7 Monitoring compliance with and the effectiveness of this document

Annual inspection of NB-BAL procedure whilst undertaken on PICU to review appropriateness of protocol for current equipment available on the unit and needs of staff. This will be the responsibility of PICU consultant medical staff and discussed at the following unit consultant meetings should modification of the protocol be required.

8 References

1. Khilnani, G. C. *et al.* Comparison of bronchoscopic and non-bronchoscopic techniques for diagnosis of ventilator associated pneumonia. *Indian J. Crit. Care Med.* **15**, 16–23 (2011).
2. Papazian, L. *et al.* Bronchoscopic or blind sampling techniques for the diagnosis of ventilator-associated pneumonia. *Am. J. Respir. Crit. Care Med.* **152**, 1982–1991 (1995).
3. Boots, R. J., Phillips, G. E., George, N. & Faoagali, J. L. Surveillance culture utility and safety using low-volume blind bronchoalveolar lavage in the diagnosis of ventilator-associated pneumonia. *Respirology* **13**, 87–96 (2008).
4. Bonvento, B. *et al.* Non-directed bronchial lavage is a safe method for sampling the respiratory tract in critically ill patient. *J. Intensive Care Soc.* **20**, 175114371881111 (2018).
5. Burmester, M. & Mok, Q. How safe is non-bronchoscopic bronchoalveolar lavage in critically ill mechanically ventilated children? *Intensive Care Med.* **27**, 716–721 (2001).

Paediatric intensive care unit (PICU)

Division E

6. Gauvin, F. *et al.* Reproducibility of Blind Protected Bronchoalveolar Lavage in Mechanically Ventilated Children. *Am. J. Respir. Crit. Care Med.* **165**, 1618–1623 (2002).
7. Greater Glasgow and Clyde NHS. Bronchoalveolar (blind) lavage (PICU). *GG&C Paediatric Guidelines* (2019). Available at: http://www.clinicalguidelines.scot.nhs.uk/ggc-paediatric-guidelines/ggc-guidelines/respiratory/bronchoalveolar-blind-lavage-picu/. (Accessed: 7th November 2019)

Equality and diversity statement

This document complies with the Cambridge University Hospitals NHS Foundation Trust service equality and diversity statement.

Disclaimer

It is **your** responsibility to check against the electronic library that this printed out copy is the most recent issue of this document.

Document management

Approval:	29 November 2019		
JDTC approval:	N/A		
Owning department:	Paediatric intensive care unit		
Author(s):	John Clark		
Pharmacist:	N/A		
File name:	NB-BAL Version1 November 2019.doc		
Supersedes:	n/a (new document)		
Version number:	1	Review date:	November 2022
Local reference:		Document ID:	101409

Clark JA, *et al. BMJ Open* 2021; 11:e056197. doi: 10.1136/bmjopen-2021-056197
