# Supplementary material for: Rapid Assay for Sick Children with Acute Lung infection Study (RASCALS): diagnostic cohort study protocol
Source: BMJ Open. 2021 Nov 29;11(11):e056197. doi: 10.1136/bmjopen-2021-056197 (PMC8634010; doi:10.1136/bmjopen-2021-056197)

Procedure modifications for lower respiratory lavage sampling on potential COVID-19 patients

Procedural equipment

- T-piece anaesthetic circuit
- Bag valve mask available
- Alternative suction device eg. Yankauer suction tip available
- End-tidal CO_2, oxygen saturation and heart rate monitoring, blood pressure monitoring
- Dressing pack
- In-line suction catheter (if not already connected to patient)
- 1x green tube connector for in-line suction catheter
- 1x Tube suction connector with female Luer-lock port (Vycon REF 801.00)
- 1x Male:Male Luer-lock connector (Vycon REF 893.00)
- Sputum trap
- 10mL or 20mL syringe (dependent on patient size)
- 3 way tap
- 0.9% sodium chloride at room temperature

COVID protective equipment

- Bag to dispose used equipment
- Personal protective equipment
- 2x bags for specimen

Syringe size

- If the patient is <=5kg, use a 10mL syringe
- If the patient is >5kg use a 20mL syringe

Draw up required amount of air and saline in syringe

- Saline: 1mL/kg to maximum 10mL saline
- Air:
    - If the volume of saline is <5mL: draw up 5mL air + (5 – volume of saline in mL)
    - If the volume of saline is >5mL: have 5mL air in the syringe

Procedure

- Assemble equipment, ensuring connections to sputum trap not too tight (2nd line on the connector)
- Close catheter to suction using 3-way tap
- Prime suction catheter with first 5mL of syringe contents (air/saline), holding down the button on the in-line suction catheter
- Advance suction catheter to below the tip of the ETT
- Deliver all of the remaining syringe contents (air + saline), holding down the button the in-line suction catheter
- Switch 3 way tap back to suction, hold down the button on the in-line suction catheter, ensuring the sputum trap remains upright

- Close the 3 way tap to suction and withdraw the suction catheter
- Turn off suction at the wall to reduce potential aerosolisation
- If patient remains stable, only 1 staff member should be in the room to complete final step if possible
- Disconnect the sputum trap and connect the in-line suction catheter back to wall suction. Place universal container lid on the specimen container.
- Dispose of used equipment in bag for highly infectious waste
- Label and package aspirated sample as required for clinical/research purposes as per current guidance

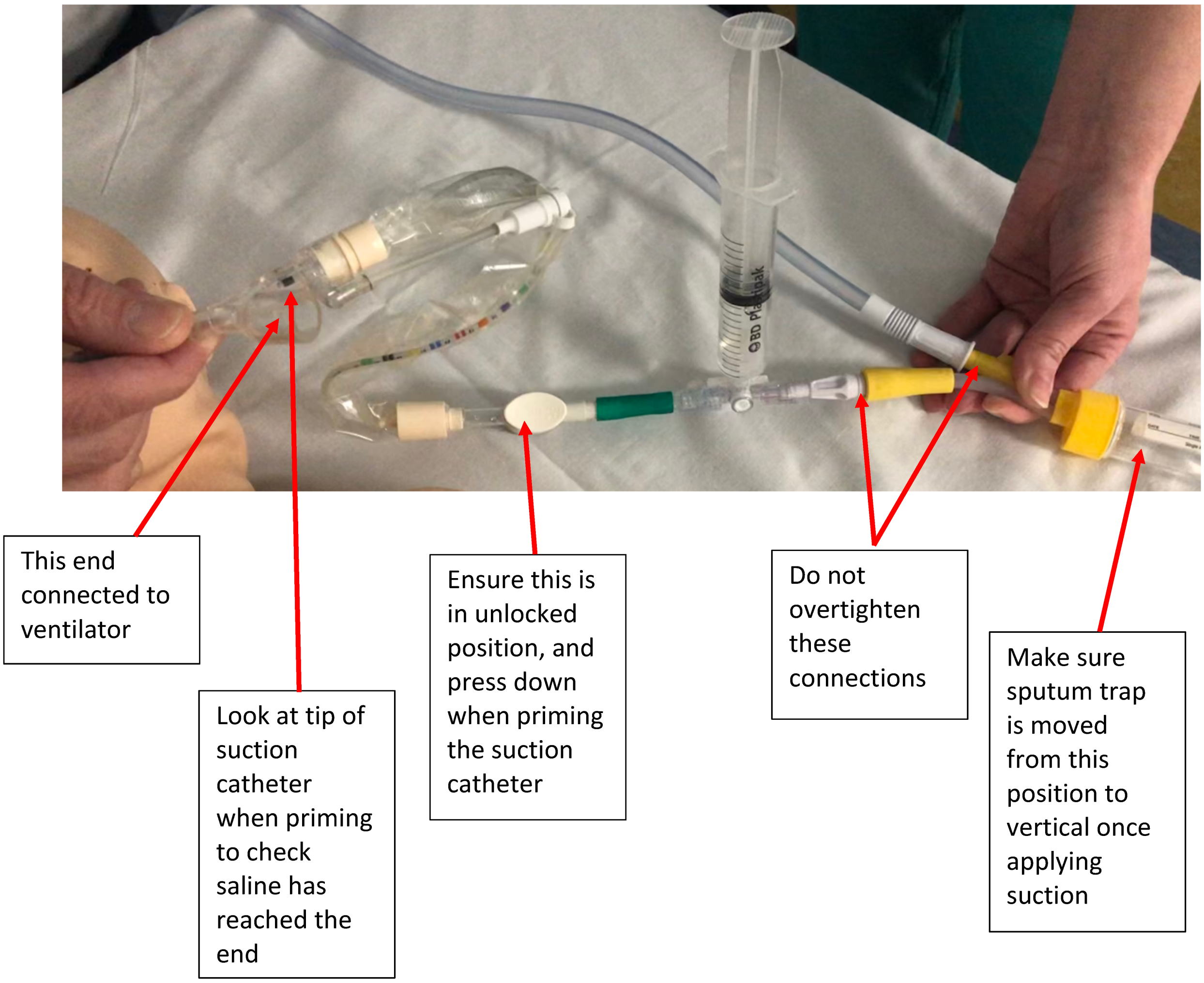

Supplement: Supplementary data [file bmjopen-2021-056197supp005.pdf]
