# Supplementary material for: Rapid Assay for Sick Children with Acute Lung infection Study (RASCALS): diagnostic cohort study protocol
Source: BMJ Open. 2021 Nov 29;11(11):e056197. doi: 10.1136/bmjopen-2021-056197 (PMC8634010; doi:10.1136/bmjopen-2021-056197)
Supplement: Supplementary data [file bmjopen-2021-056197supp006.pdf]

BMJ Publishing Group Limited (BMJ) disclaims all liability and responsibility arising from any reliance placed on this supplemental material which has been supplied by the author(s)

Confidential

Clinical team survey

Thank you for participating in RASCALS.

Lower respiratory tract infection is a leading cause of admission to paediatric intensive care, and indication for antimicrobial therapy. It is difficult to rationalise antimicrobial therapy given these patients are so unwell, and with current microbiology techniques taking several days to provide a result. Rapid diagnostic tests have the potential to reduce the exposure of our patients to antimicrobials and provide earlier rationalisation of therapy.

We are investigating the impact of a rapid diagnostic test, a Taq-Man array, on clinical practice for ventilated children who are receiving antimicrobial treatment for lower respiratory tract infection. The following survey is to determine whether changes are implemented following Taq-man array results and would appreciate your assistance with this.

We request that you complete this survey at the time the Taq-Man array result becomes available. This survey should take less than 5 minutes. We are incredibly grateful for your time.

Kind regards,

The Paediatric Intensive Care Unit Research Team

RASCALS researchers: Dr Nazima Pathan, Dr John Clark, Dr Iain Kean, Dr Estée Török, Prof Gordon Dougan, Prof Stephen Baker, Dr Vilas Navapurkar, Ms Esther Daubney & Ms Deborah White.

Phone: 01223 336883

Email: np409@medschl.cam.ac.uk

1)	Did the Taq-Man result impact on the ongoing antimicrobial type prescribed by the clinical team?	◯ No<br>◯ Yes antimicrobial therapy was de-escalated<br>◯ Yes, antimicrobial therapy was escalated
2)	Did the Taq-Man result impact on the clinical team's decision regarding planned duration of antimicrobial therapy?	◯ No<br>◯ Yes, there was a plan for longer duration of antimicrobial therapy<br>◯ Yes, there was a plan for shorter duration of antimicrobial therapy<br>◯ Yes, antimicrobial therapy was ceased
3)	Did the Taq-Man result contribute to a change in the patient's isolation status?	◯ No, the test had no impact on isolation status<br>◯ Yes, isolation was ceased due to the Taq-Man result<br>◯ Yes, isolation was continued due to the Taq-Man result
4)	If you have any other comments regarding the Taq-Man result, antimicrobial prescription and isolation status please write here	__________________________________

Clark JA, *et al. BMJ Open* 2021; 11:e056197. doi: 10.1136/bmjopen-2021-056197
