# Supplementary material for: Rapid Assay for Sick Children with Acute Lung infection Study (RASCALS): diagnostic cohort study protocol
Source: BMJ Open. 2021 Nov 29;11(11):e056197. doi: 10.1136/bmjopen-2021-056197 (PMC8634010; doi:10.1136/bmjopen-2021-056197)
Supplement: Supplementary data [file bmjopen-2021-056197supp007.pdf]

Department of Paediatric Intensive Care

Dr Roddy O'Donnell
Dr Shruti Agrawal
Dr David Inwald
Dr Riaz Kayani
Dr Girish Neelegowda
Dr Nazima Pathan
Dr Stewart Reid
Dr Francesc Torres

Cambridge University Hospitals NHS
NHS Foundation Trust

Addenbrooke's Hospital
Hills Road
Cambridge CB2 0QQ

Switchboard: 01223 245151
www.addenbrookes.org.uk

Rapid Assay for Sick Children with Acute Lung infection Study
Staff focus group – experiences of TaqMan diagnostic array

RASCALS

Rapid Assay for Sick Children with Acute Lung infection Study

Thank you for taking the time to consider participating in a focus group for 'RASCALS' – the Rapid Assay for Sick Children with Acute Lung infection Study.

Before you decide to participate it is important for you to understand why the research is being done and what it will involve. Please take time to read the following information carefully. Do ask us if there is anything that is not clear or if you would like more information. Take time to decide whether or not you wish to take part.

Kind regards,
The Paediatric Intensive Care Unit Research Team

RASCALS researchers: Dr Nazima Pathan, Dr John Clark, Dr Iain Kean, Dr Estée Török, Prof Gordon Dougan, Prof Stephen Baker, Dr Vilas Navapurkar, Ms Esther Daubney & Ms Deborah White.
Phone: 01223 336883
Email: np409@medschl.cam.ac.uk

*This study has been co-sponsored by Cambridge University Hospitals NHS Foundation Trust and the University of Cambridge. Any reference to 'we' or 'us' in these documents refers to these entities.

Clark JA, *et al. BMJ Open* 2021; 11:e056197. doi: 10.1136/bmjopen-2021-056197

Focus group participant information:
Rapid Assay for Sick Children with Acute Lung infection Study

What is the purpose of this study?
The Department of Paediatric Intensive Care at Addenbrooke's Hospital is actively involved in research in children who are critically ill. We want to do research that helps us develop better ways of diagnosing and treating children admitted to our unit. Our research team would like to talk to you about the work that we are doing.

In the RASCALS project we have been researching the lung microbiome in critical care, rapid diagnostic tools for pneumonia and biomarkers of COVID-19 in children. In this focus group however, we are specifically interested in staff experiences of the TaqMan respiratory diagnostic array, which has been trialled on the unit since April 2020.

Why have I been asked to participate?
We are interested in capturing the experiences of staff currently working on the PICU in relation to the TaqMan diagnostic array. We are the first team in the world to integrate this test into a paediatric clinical service – we would like to capture this experience to drive future research and implementation of this test.

Who is organising the study?
The study is being organised by the Department of Paediatric Intensive Care by Dr Nazima Pathan and Dr John Clark, here at Addenbrooke's Hospital. This work is supervised by the University of Cambridge. No specific payments will be made to any of the staff involved for including your child in this study other than their normal salary.

Does I have to take part?
Participation in this focus group is completely voluntary.

What will happen if I take part?
A member of the research team will conduct a focus group discussion with you within a small group. This discussion will take place over a maximum of 30 minutes. This will only take place where there is sufficient staffing and time, so as to not impact on the clinical care of our patients. The research team member will provide broad signposting for areas to discuss but the discussion itself is open to the group. We are keen to obtain both positive and negative aspects of the diagnostic array and its implementation. With your permission the conversation will be recorded for coding by the research team. Your identity will be anonymised, however we would like to know your clinical role in the team to assist interpretation of our research findings.

What happens at the end of the study?
The focus group discussion will be anonymised and coded by the research team. We will use the themes and some direct quotes discussed by the focus group and publish these findings alongside our scientific findings of the RASCALS project.

What if I want to withdraw from the study?
You may withdraw your consent to participate from the project at any time. Withdrawal of consent will be a confidential process and will not fed back to staff or management within the clinical service.

Clark JA, *et al. BMJ Open* 2021; 11:e056197. doi: 10.1136/bmjopen-2021-056197

Who has reviewed the study?
All research in the NHS is looked at by independent group of people, called a Research Ethics Committee, to protect your interests. This study has been reviewed by the research advisory committee of NHS Cambridge University Hospitals NHS Foundation Trust and the research ethics committee of Bradford-Leeds.

Thank you for considering your participation in this research project.

Clark JA, *et al. BMJ Open* 2021; 11:e056197. doi: 10.1136/bmjopen-2021-056197
