# Supplementary material for: Rapid Assay for Sick Children with Acute Lung infection Study (RASCALS): diagnostic cohort study protocol
Source: BMJ Open. 2021 Nov 29;11(11):e056197. doi: 10.1136/bmjopen-2021-056197 (PMC8634010; doi:10.1136/bmjopen-2021-056197)
Supplement: Supplementary data [file bmjopen-2021-056197supp008.pdf]

Department of Paediatric Intensive Care

Dr Roddy O'Donnell
Dr Shruti Agrawal
Dr David Inwald
Dr Riaz Kayani
Dr Girish Neelegowda
Dr Nazima Pathan
Dr Stewart Reid
Dr Francesc Torres

Cambridge University Hospitals NHS
NHS Foundation Trust

Addenbrooke's Hospital
Hills Road
Cambridge CB2 0QQ

Switchboard: 01223 245151
www.addenbrookes.org.uk

Rapid Assay for Sick Children with Acute Lung infection Study
Staff focus group – experiences of TaqMan diagnostic array

Rapid Assay for Sick Children with Acute Lung infection Study

Thank you for taking the time to consider participating in the focus group for 'RASCALS' – the Rapid Assay for Sick Children with Acute Lung infection Study and reading the information sheets.

If you would like to participate in the focus group, please read and sign the following forms.

We will provide you with one copy of this form and retain a second copy securely in our research department at the hospital.

Kind regards,

The Paediatric Intensive Care Unit Research Team
RASCALS researchers: Dr Nazima Pathan, Dr John Clark, Dr Iain Kean, Dr Estée Török, Prof Gordon Dougan, Prof Stephen Baker, Dr Vilas Navapurkar, Ms Esther Daubney & Ms Deborah White.
Phone: 01223 336883
Email: np409@medschl.cam.ac.uk

Clark JA, *et al. BMJ Open* 2021; 11:e056197. doi: 10.1136/bmjopen-2021-056197

**Participant identification number**:		**Initials**
1.	I have read the information sheet on this project, dated ___/___/____ (Version________) and have been given a copy to keep. I have been able to ask questions about the project and I understand why the research is being done and any risks involved.	
2.	I know how to contact the research team if I need to, and how to get information about the results of the research.	
3.	I understand that the focus group interview will be recorded, and this data will be transcribed with participant identity anonymised by the research team.	
4	I understand that direct quotes from the deidentified interview transcript may be included in published research findings.	
5.	I understand that any information collected as part of this study will be stored securely in line with current NHS and University guidelines. I understand that only members of the clinical project team will have access to my identifiable information; all others analysing my data will only have access to anonymised data. I understand that my data will be retained for 15 years.	
6.	I understand that the information collected may be used to support other research in the future, and may be shared anonymously with other academic and commercial researchers external to the project within the UK and beyond	
7.	I consent to taking part in this study	

Clark JA, *et al. BMJ Open* 2021; 11:e056197. doi: 10.1136/bmjopen-2021-056197

Written consent

Carer

Name (Capitals):____

Signature:________________________________ Date: dd/mm/yyyy

Relationship to patient: ___________________________________

Person obtaining consent

Name (Capitals):____

Signature:________________________________ Date: dd/mm/yyyy

Role: ______________________________

Electronic consent

Carer name (Capitals):____

Person obtaining consent

Name (Capitals):____

Signature:________________________________ Date: dd/mm/yyyy

Role: ______________________________

Verbal consent

Carer name (Capitals):____

Person obtaining consent

Name (Capitals):____

Signature:________________________________ Date: dd/mm/yyyy

Role: ______________________________

Witness to verbal consent

Name (Capitals):____

Signature:________________________________ Date: dd/mm/yyyy

Role: ______________________________

Primary investigator countersign for electronic and unwitnessed verbal consent

Dr Nazima Pathan ______________________________ Date: dd/mm/yyyy

Clark JA, *et al. BMJ Open* 2021; 11:e056197. doi: 10.1136/bmjopen-2021-056197
