# Supplementary material for: Rapid Assay for Sick Children with Acute Lung infection Study (RASCALS): diagnostic cohort study protocol
Source: BMJ Open. 2021 Nov 29;11(11):e056197. doi: 10.1136/bmjopen-2021-056197 (PMC8634010; doi:10.1136/bmjopen-2021-056197)
Supplement: Author's manuscript [file bmjopen-2021-056197.draft_revisions.pdf]

BMJ Open is committed to open peer review. As part of this commitment we make the peer review history of every article we publish publicly available.

When an article is published we post the peer reviewers' comments and the authors' responses online. We also post the versions of the paper that were used during peer review. These are the versions that the peer review comments apply to.

The versions of the paper that follow are the versions that were submitted during the peer review process. They are not the versions of record or the final published versions. They should not be cited or distributed as the published version of this manuscript.

BMJ Open is an open access journal and the full, final, typeset and author-corrected version of record of the manuscript is available on our site with no access controls, subscription charges or pay-per-view fees (http://bmjopen.bmj.com).

If you have any questions on BMJ Open's open peer review process please email info.bmjopen@bmj.com

BMJ Open

Rapid Assay for Sick Children with Acute Lung infection Study (RASCALS) – diagnostic cohort study protocol

Journal:	*BMJ Open*
Manuscript ID	bmjopen-2021-056197
Article Type:	Protocol
Date Submitted by the Author:	10-Aug-2021
Complete List of Authors:	Clark, John; University of Cambridge, Department of Paediatrics; Cambridge University Hospitals NHS Foundation Trust<br>Kean, Iain; University of Cambridge, Department of Paediatrics<br>Curran, Martin; Clinical Microbiology and Public Health Laboratory<br>Khokhar, Fahad; University of Cambridge, Cambridge Institute of Therapeutic Immunology and Infectious Disease<br>White, Deborah; Cambridge University Hospitals NHS Foundation Trust<br>Daubney, Esther; Cambridge University Hospitals NHS Foundation Trust<br>Conway-Morris, Andrew; Cambridge University Hospitals NHS Foundation Trust<br>Navapurkar, Vilas; Cambridge University Hospitals NHS Foundation Trust<br>Bartholdson-Scott, Josefin; University of Cambridge, Cambridge Institute of Therapeutic Immunology and Infectious Disease<br>Maes, Mailis; University of Cambridge, Cambridge Institute of Therapeutic Immunology and Infectious Disease<br>Bousfield, Rachel; Cambridge University Hospitals NHS Foundation Trust<br>Gouliouris, Theodore; University of Cambridge, Department of Medicine<br>Agrawal, Shruti; University of Cambridge, Department of Paediatrics; Cambridge University Hospitals NHS Foundation Trust<br>Inwald, David; Cambridge University Hospitals NHS Foundation Trust<br>Zhang, Zhenguang; University of Cambridge, Department of Paediatrics<br>Torok, Mili; University of Cambridge School of Clinical Medicine, Department of Medicine<br>Baker, Stephen; University of Cambridge, Cambridge Institute of Therapeutic Immunology and Infectious Disease<br>Pathan, Nazima; University of Cambridge, Department of Paediatrics; Cambridge University Hospitals NHS Foundation Trust
Keywords:	Molecular diagnostics < INFECTIOUS DISEASES, Respiratory infections < THORACIC MEDICINE, Paediatric intensive & critical care < ANAESTHETICS

SCHOLARONE™
Manuscripts

I, the Submitting Author has the right to grant and does grant on behalf of all authors of the Work (as defined in the below author licence), an exclusive licence and/or a non-exclusive licence for contributions from authors who are: i) UK Crown employees; ii) where BMJ has agreed a CC-BY licence shall apply, and/or iii) in accordance with the terms applicable for US Federal Government officers or employees acting as part of their official duties; on a worldwide, perpetual, irrevocable, royalty-free basis to BMJ Publishing Group Ltd ("BMJ") its licensees and where the relevant Journal is co-owned by BMJ to the co-owners of the Journal, to publish the Work in this journal and any other BMJ products and to exploit all rights, as set out in our licence.

The Submitting Author accepts and understands that any supply made under these terms is made by BMJ to the Submitting Author unless you are acting as an employee on behalf of your employer or a postgraduate student of an affiliated institution which is paying any applicable article publishing charge ("APC") for Open Access articles. Where the Submitting Author wishes to make the Work available on an Open Access basis (and intends to pay the relevant APC), the terms of reuse of such Open Access shall be governed by a Creative Commons licence – details of these licences and which Creative Commons licence will apply to this Work are set out in our licence referred to above.

Other than as permitted in any relevant BMJ Author's Self Archiving Policies, I confirm this Work has not been accepted for publication elsewhere, is not being considered for publication elsewhere and does not duplicate material already published. I confirm all authors consent to publication of this Work and authorise the granting of this licence.

For peer review only

Rapid Assay for Sick Children with Acute Lung infection Study (RASCALS) – diagnostic cohort study protocol

John A Clark MBBS [1, 2*], Iain Kean PhD [1], Martin D Curran PhD [3], Fahad Khokhar BSc [4], Deborah White MSc [2], Esther Daubney BSc [2], Andrew Conway Morris PhD [2], Vilas Navapurkar FFICM [2], Josefin Bartholdson-Scott PhD [4], Mailis Maes MPhil [4], Rachel Bousfield BMBS [2, 3], Theodore Gouliouris PhD FRCPath [2, 3], Shruti Agrawal MD FRCPCH [1, 2], David Inwald PhD FRCPCH [2], Zhenguang Zhang PhD [1], M. Estée Török PhD, FRCP, FRCPath [2, 3], Stephen Baker PhD [4] and Nazima Pathan PhD FRCPCH [1, 2*]

1. Department of Paediatrics, University of Cambridge
2. Cambridge University Hospitals NHS Foundation Trust
3. Public Health England, Clinical Microbiology and Public Health Laboratory, Cambridge
4. Cambridge Institute of Therapeutic Immunology and Infectious Disease, University of Cambridge

*Corresponding author: Dr John Clark, Department of Paediatrics, Level 8, Addenbrooke's Hospital, Cambridge Biomedical Campus, Cambridge CB2 0QQ. Email: jac302@cam.ac.uk

Acknowledgements: The authors would like to thank the staff at Addenbrooke's Hospital for supporting the development and implementation of this study; Helen Starace and Colin Hamilton (physiotherapists) for assisting with developing NB-BAL sampling procedure; Adam Palmer (PICU data manager) for providing admissions data; and Claire Jenkins (scientist) for assisting with sample workflow.

Trial sponsor contact: Stephen Kelleher, Cambridge University Hospitals NHS Foundation Trust, Hills Rd, Cambridge CB2 0QQ; research@addenbrookes.nhs.uk Ph: +441223217418

Word count: 3233

Key words: Pathology, Molecular; Pneumonia; Pneumonia, Ventilator-Associated; Intensive Care Units, Pediatric; Antimicrobial stewardship

For peer review only

Abstract

Introduction: Lower respiratory tract infection (LRTI) is the most commonly treated infection in critically ill children. Pathogens are infrequently identified on routine respiratory culture, and this is a time-consuming process. A syndromic approach to rapid molecular testing that includes a wide range of bacterial and fungal targets has the potential to aid clinical decision making and reduce unnecessary broad spectrum antimicrobial prescribing. Here we describe a single-centre prospective cohort study investigating the use of a 52-pathogen TaqMan array card (TAC) for LRTI in the Paediatric Intensive Care Unit (PICU).

Methods and analysis: Critically ill children with suspected LRTI will be enrolled to this 100 patient single centre prospective observational study. Samples will be obtained via routine non-bronchoscopic bronchoalveolar lavage (NB-BAL) which will be sent for standard microbiology culture in addition to TAC. A blood draw will be obtained via any existing vascular access device. The primary outcomes of the study will be i) concordance of TAC result with routine culture and 16S rRNA gene sequencing and ii) time of diagnostic result from TAC versus routine culture. Secondary outcomes will include impact of the test on total antimicrobial prescriptions, a description of the inflammatory profile of the lung and blood in response to pneumonia and a description of the clinical experience of medical and nursing staff using TAC.

Ethics and dissemination: This study has been approved by the Yorkshire and the Humber – Bradford Leeds Research Ethics Committee (REC reference 20/YH/0089). Results will be published in peer reviewed publications and international conferences.

Trial registration number: NCT04233268; Pre-results

Strengths and limitations of this study

- This study describes clinical application of a customisable rapid diagnostic respiratory microarray in critically ill children. This has not previously been described in scientific literature.
- The sampling technique for this project is NB-BAL, which is a reliable sampling method for deep respiratory samples. Most previous molecular diagnostic studies of

For peer review only

LRTI in children have utilised upper airway samples, or endotracheal tube (ETT) secretions which may be prone to contamination.

- This is a single centre study, and a multi-centre study will be required to determine feasibility and cost efficiency of this diagnostic method on a national scale.
- There is no universally agreed diagnostic criteria for pneumonia in children, therefore clinical suspicion of pneumonia by the treating team rather than a specific set of diagnostic criteria are being used for enrolment.

Introduction

Lower respiratory tract infection (LRTI) is a leading cause of admissions to paediatric intensive care units (PICUs) in the UK, and greatest worldwide cause of mortality in young children.[1,2] PICU physicians regularly prescribe antimicrobial therapy for LRTI, as it is difficult to ascertain whether children have an underlying primary or secondary bacterial infection and withholding or delaying treatment where indicated poses significant risk.[3] Clinical prediction scores for pneumonia have low specificity in children,[4,5] and infection on standard microbiological tests is rarely confirmed.[6]

Rapid diagnostic tests have the potential to reduce untargeted antimicrobial use.[7] Most rapid respiratory diagnostic panels in routine clinical use include common viruses and a limited number of atypical bacterial pathogens such as *Legionella pneumophila* and *Mycoplasma pneumoniae* which are difficult to grow on culture.[8,9] The TaqMan array card (TAC) (Thermo Fisher Scientific, California) is a probe-based qPCR assay undertaken in a 384 well plate format. Advantages of TAC are the ability to customise molecular targets – allowing new targets to be incorporated according to local epidemiology, and the ability to run multiple samples on a single card improving cost efficiency.[10] This may include up to eight clinical samples per card depending on the number of targets sought per sample. Other rapid molecular diagnostic tests, such as the FilmArray Pneumonia Panel (BioFire Diagnostics, Utah, USA) and Curetis Unyvero pneumonia cartridge (Holzgerlingen, Germany) whilst offering the advantage of being able to be undertaken as a point of care test with sufficient staffing and training, are more limited by the number of assays that can be tested on each platform and are constrained to testing manufacturer defined molecular targets. [11–13]

For peer review only - http://bmjopen.bmj.com/site/about/guidelines.xhtml

TAC performs reliably on deep respiratory samples obtained from adults with suspected ventilator associated pneumonia (VAP).[14] In children, studies of TAC as a respiratory infection diagnostic are limited. On extensive search of medical literature, we identified two key studies of TAC in children with suspected pneumonia. One utilised nasopharyngeal/oropharyngeal and sputum in hospitalised children.[15] The other obtained endotracheal aspirate samples in 25 children with suspected hospital acquired pneumonia with a limited TAC inclusive of 7 bacteria.[16] TAC improved diagnostic yield of samples in both studies,[16] but was not used for diagnostic purposes. Given the heavy use of systemic antimicrobial therapy in the care of critically ill children, evaluation of TAC to guide antimicrobial therapy requires further evaluation. We are therefore undertaking this study to understand the performance and impact of TAC in the PICU setting.

Hypothesis

TAC will provide greater sensitivity and a faster turnaround time than standard microbiology tests for the diagnosis of LRTI in critically unwell children.

Study objectives and measures

Objectives

Primary

1. Determine the sensitivity of TAC in the detection of lower respiratory bacterial pathogens.
2. Compare time to diagnosis of TAC versus standard microbiology culture in the diagnosis of LRTI.

Secondary

1. Describe the microorganisms detected on TAC that were not detected using standard diagnostic tests.
2. Describe the lung microbiome of critically ill children with LRTI using 16S rRNA gene sequencing.
3. Understand the impact of TAC on total antimicrobial prescriptions in PICU by total prescriptions and by antimicrobial class.
4. Describe the impact of TAC on antimicrobial decision making according to PICU consultants.
5. Describe the host inflammatory response to LRTI in the lung and blood according to pathogen present.

For peer review only

6. Describe the experience of a tertiary PICU in the implementation of TAC in the clinical setting.

Outcome measures

Primary

1. TAC result (cycle threshold value).
2. Respiratory culture result (colony forming units/ml).
3. Respiratory viral panel result (cycle threshold value).
4. Time to result (hours).

Secondary

1. TAC result (cycle threshold [Ct] value).
2. Blood culture result.
3. Total days of antimicrobial prescriptions in PICU – % total days of PICU admission and days free of antimicrobial therapy in PICU at 28 days.
4. Reported n (%) of change in antimicrobial prescription type and duration, and n (%) of children able to move out of side rooms due to exclusion of infectious pathogens.
5. Days free of mechanical ventilation at 28 days following PICU admission.
6. Days free of PICU at 28 days following PICU admission.
7. 16S rRNA gene sequencing result – n (%) of total reads, Shannon's diversity index
8. Cytokine concentration (pg/ml) in NB-BAL samples and blood.
9. Semi-structured interview thematic analysis - description of implementation and interpretation considerations of TAC in the PICU.

Methods and analysis

Setting

Patients will be enrolled to the study in a 13 bed PICU in the East of England. The PICU manages neurosurgical and trauma cases but does not support extracorporeal membrane oxygenation or cardiac surgical patients. There are twice weekly microbiology rounds on the PICU in which all patients are discussed by the clinical and microbiology teams. A microbiology specialty registrar or consultant is available for specialist advice in this centre 24 hours per day.

Eligibility criteria

The eligibility criteria are:

For peer review only

1. The child is aged less than 18 years old;
2. the child is receiving mechanical ventilation at the time of enrolment; and
3. the child is commencing or already receiving antimicrobial therapy to treat suspected or confirmed LRTI.

The exclusion criteria are:

1. The patient has a non-survivable illness and is no longer on an active treatment pathway and/or
2. the child is <37 weeks corrected gestation.

These criteria ensure the patient is able to have samples obtained via NB-BAL. Enrolment of children based on antimicrobial prescription for LRTI has been selected rather than the use of clinical features for pneumonia, given there is no consistent and reliable clinical scoring system for this condition.[17,18] Premature infants have been excluded from enrolment due to the sampling procedure not being tested, to our knowledge in this group of patients, and these infants having distinct pneumonia aetiology that would require separate evaluation.

Selection of participants

All children admitted to PICU at the study centre will be screened for enrolment into the study by nursing and medical staff working on the unit. A deferred consent process for up to 48 hours will allow carers to be approached sensitively by the research team, whilst ensuring samples are obtained within a reasonable timeframe to maximise yield. Consent will be obtained via written, electronic, and verbal formats via research nursing staff, and permission also obtained for COVID-19 related work (appendix A and B). Co-enrolment will be permitted in this study so long as there is no impact on the primary outcomes of either project.

Sample size measurement

This study aims to achieve an increase in the sensitivity of lower respiratory tract culture using TAC by 60%. Previous study on this unit has identified that 22% of cases of possible LRTI are confirmed on culture.[19] To detect an increase in bacterial confirmation by 60%, a total of 85 patients are required as per power calculation (figure 1).[20] The total has been rounded up to 100 to account for possible sampling related issues that may occur.

For peer review only - http://bmjopen.bmj.com/site/about/guidelines.xhtml

Intervention

Diagnostic TAC will be undertaken on NB-BAL sample obtained as soon as possible following the time of enrolment (Figure 2). The same sample will be used for standard microbiology culture in all patients; however, the clinical team will determine whether viral qPCR panel, extended fungal or mycobacterial cultures or investigations are also indicated. Samples will only be processed during business hours Monday-Friday due to availability of research and laboratory staff.

Samples will be obtained as part of routine practice by physiotherapy, nursing and medical staff that routinely perform the sampling procedure. The results of the test will be reported alongside routine tests, and impact of these results will be investigated.

Sampling and Laboratory Procedures

NB-BAL sampling

Deep respiratory samples will be obtained at the time of enrolment via non-bronchoscopic bronchoalveolar lavage, with some modifications made to the original procedure given SARS-CoV-2 (appendix C and D). Saline lavage volume instilled will be 1ml/kg of patient weight to a maximum of 10mL. Saline is delivered via the in-line suction catheter system and the sample collected in a universal container via a sputum trap. This approach minimises risk of aerosolisation of pathogens such as SARS-CoV-2. Samples will be immediately delivered to the microbiology containment level 3 laboratory and stored at 4°C until staff are available to process the samples, between 8AM-5PM weekdays. Samples will be split into aliquots for microbiology culture, nucleic acid extraction, and any additional clinical tests required.

Nucleic acid extraction

Up to 750 µl of sample will be added to a 2mL microtube containing a mixture of ceramic beads with 750 µl of L6 buffer (Qiagen). A minimum of 100uL will be required for extraction and brought up to 750 µl with nuclease free water if low volume. The sample will then be vortexed and incubated for 10 minutes. Samples will then be processed using an EZ1 virus mini kit (v2.0) using an EZ1 advanced XL (Qiagen) in up to 14 samples per run including an L6 buffer-RNase free water negative control.[21]

For peer review only - http://bmjopen.bmj.com/site/about/guidelines.xhtml

TaqMan array card

A custom screening panel was developed and validated via an adult intensive care study that took place in this institution.[22] Retrospective review of organisms identified on routine tests for severe LRTI in this PICU demonstrated good coverage by this panel.[19] The targets of the panel are as shown in table 1. Many pathogens are assigned two targets to reduce false positive test interpretations. A target for the MecA gene has been included as it is commonly present in methicillin-resistant *Staphylococcus aureus*. The TAC includes endogenous control RNase P, internal control MS2 and 18S rRNA gene. TAC configuration was altered on 05/02/2021, to include SARS-CoV-2 targets, and opportunistically incorporate *Leptospira* as this organism was of relevance to the adult ICU service also using the cards. Using the same card for the adult and paediatric service allows samples to be processed in batches of up to four, reducing waste of empty lanes in the card, and minimises laboratory handling time.

Table 1: Molecular targets of the TaqMan diagnostic card for lower respiratory tract infection

Type	Target	Number of targets
Bacterial	16S rRNA* gene	2
	Acinetobacter baumannii	3
	Bacteroides fragilis	1
	Bordetella pertussis	2
	Chlamydia pneumoniae	1
	Chlamydia psittaci	1
	Coxiella burnetti	1
	Elizabethkingia meningoseptica	2
	Enterobacter cloacae	2
	Enterobacteriaceae	1
	Enterobacteriaceae Proteus	1
	*Enterococcus faecalis**	1
	*Enterococcus faecium**	2
	Escherichia coli	2
	Haemophilus influenzae	2
	Klebsiella pneumoniae	2
	Legionella pneumophilia	1
	Legionella spp	1
	Moraxella catarrhalis	1
	Morganella morganii	1
	Mycobacterium spp	1

For peer review only - http://bmjopen.bmj.com/site/about/guidelines.xhtml

	Mycoplasma pneumoniae	2
	Mycobacterium tuberculosis	2
	Neisseria meningitidis	1
	Pseudomonas aeruginosa	2
	Serratia marcescens	2
	Staphylococcus aureus	2
	Staphylococcus – coagulase negative	1
	Staphylococcus epidermidis	1
	Staphylococcus - PVL toxin	1
	Stenotrophomonas maltophilia	1
	Streptococcus pneumoniae	2
	Streptococcus pyogenes	2
	Streptococcus spp	2
Viral	Adenovirus	2
	Bocavirus	1
	Cytomegalovirus	1
	Epstein Barr Virus	1
	Enterovirus	2
	Herpes simplex virus	2
	Human coronavirus NL63	1
	Human coronavirus OC43/HKU1	1
	Human coronavirus OC43	1
	Human coronavirus 229E	1
	Human metapneumovirus	1
	Human parainfluenza virus 1	2
	Human parainfluenza virus 2	1
	Human parainfluenza virus 3	2
	Human parainfluenza virus 4	1
	Influenza A	2
	Influenza A – H1 (2009)	1
	Influenza A – H3	1
	Influenza B	2
	Parechovirus	1
	Respiratory syncytial virus (any)	1
	Respiratory syncytial virus A	1
	Respiratory syncytial virus B	1
	Rhinovirus	2
Fungal	*Aspergillus* 28S rRNA gene	2

For peer review only

	Aspergillus fumigatus	1
	Candida albicans	1
	Candida species	1
	Fungal 18S rRNA gene	1
	Pneumocystis jirovecii	1
AMR gene	*mecA*	1
Controls	18S rRNA gene	1
	MS2 internal control	2
	RNase P internal control	1
**Total wells**		**96**

*In the four enrolments occurring from 5 February 2021 onwards, these targets were replaced to include 3 targets for SARS-CoV-2, 1 target for *Leptospira* and an additional target for legionella species.

For each sample, 50 µl of total nucleic acid is added to 50 µl of TaqMan Fast Virus 1-step mastermix (Thermo Fisher) and 100 µl of RNase free water. A total of 98 µl is then added to 2 lanes of the array. Each lane comprises 48 molecular targets, with the array configured to 96 targets of interest. Therefore, 4 patient samples are loaded into 2 lanes each for a total of 8 lanes per plate.

The RT-PCR will be undertaken on a QuantStudio 7 Flex (Thermo Fisher) according to the following validated protocol: 50°C for 5 minutes, 95°C for 20 seconds, 45 cycles of 95°C for 1 second, 60°C for 20 seconds.[14] qPCR Ct values with clear amplification curves will be reported and documented on the electronic medical record.

Conventional pathogen testing

Samples will be processed according to Public Health England laboratory standards. For patients that are not immunocompromised, standard media will be used. The sample will be inoculated on chocolate agar and incubated at 35-37°C supplemented with 5-10% CO_2.[23] If the patient is immunocompromised, supplementary media will be used, and MacConkey agar and Mannitol salt/Chromogenic agar will be used with the sample incubated in air.[23] Significant growth constitutes >10^4 cfu/mL on NB-BAL samples, or >10^5 cfu/mL on endotracheal tube (ETT) aspirate. Bacterial organisms will be identified to species or genus level using MALDI-TOF mass spectrometry (Bruker Daltonics, Coventry, UK).

For peer review only - http://bmjopen.bmj.com/site/about/guidelines.xhtml

Antimicrobial susceptibility testing will be performed using disc diffusion following EUCAST guidelines.[24]

An in-house multiplex RT-PCR assay will be used for testing NPA and NB-BAL samples for common respiratory viruses: adenovirus, enterovirus, human metapneumovirus, influenza A and B viruses, parainfluenza virus, rhinovirus, and respiratory syncytial virus. This assay is only requested when determined to be of relevance by the clinical team.

If clinically suspected, presence of *Aspergillus* spp will be tested by culture on Sabouraud dextrose agar with chloramphenicol. Serum will also be tested for the presence of galactomannan antigen. If clinically suspected PCR assay will be used to detect presence of *Pneumocystis jirovecii*.

16S rRNA gene sequencing

Nucleic acid samples will be quantified using a Qubit 4 fluorometer and Qubit dsDNA HS assay kit (Q32854) (ThermoFisher Scientific, Waltham, MA, USA). DNA fragment size and quality will be assessed using an Agilent TapeStation 4200 (Agilent, Santa Clara, CA, USA).

After quantification, a sequencing library will be prepared using a 16S rRNA gene Barcoding Kit (SQK – 16S024), then loaded onto a FLO-MIN106D R9.4.1 flow cell. Sequencing will be undertaken using a MinION nanopore sequencing device (Oxford Nanopore Technologies, UK). Base calling will be undertaken using the EPI2ME platform and data will be stored as FASTQ files. De-multiplexing will be completed with guppy_barcoder v5.0.11, adapters will be trimmed with porechop 0.2.4, and reads filtered with NanoFilt v2.8.0. Filtered reads will be classified using Kraken2.[25] Results will be for research purposes only and not distributed to the clinical team.

Blood sampling

Blood will be obtained for research if the child is undergoing venepuncture for clinical purposes or if there is an existing vascular access device. The maximum volume obtained will be 1ml/kg to a maximum 10ml in keeping with World Health Organisation Guidelines.[26] Collected blood will be stored in a 4°C refrigerator on the ward until the research team are able to process samples. EDTA tubes will be spun at 1300g for 10 minutes at 4°C, and aliquots stored at -20°C until batch processing.

For peer review only - http://bmjopen.bmj.com/site/about/guidelines.xhtml

Cytokine assay

Cytokines will be quantified from NB-BAL and plasma samples using a Bio-Plex Pro Human Cytokine Screening 48-plex kit (Bio-Rad) using standard methods.[27] This assay will be undertaken on aliquots of NB-BAL stored at -80°C until batch processing. NB-BAL will be un-diluted whilst plasma will be diluted with standard diluent HB as per manufacturer recommendations dependent on limits of detection according to the generated standard curves.

Survey and Semi-structured interviews

PICU Consultants will be surveyed regarding their antimicrobial decision making after TAC result becomes available (Appendix E). Survey invitation will be sent via RedCAP to the hospital email address of the duty PICU consultant when the TAC result becomes available.

Semi-structured interviews will be undertaken with PICU nursing and medical staff at the end of the study. They will be advertised using posters and via the internal newsletter to staff. Voluntary consent will be obtained (Appendix F and G). Sessions will be facilitated by a member of the research team and recorded. Themes and descriptions will be analysed using NVIVO 11.[28] These interviews will capture the experience of staff in implementing TAC into clinical practice, the benefits and downsides to the test, and interpretation of the test.

Patient and public involvement

Carers of children admitted to PICU identified rapid molecular diagnostic tests for early rationalisation of antimicrobial therapy to be a high priority area for research. They ranked this 4th of 73 potential national priority areas for PICU research in a 2019 multi-centre survey.[29] As this study is investigating acute clinical decision making of critical care clinicians, patients were not involved in the design of scientific study methodology. The research team will be engaging interested families who have had children admitted to this PICU for their input in the dissemination of research findings and for additional feedback on the design of patient information and consent materials.

Study status

Enrolment commenced on 10/04/2020 and enrolment is expected to be completed in June 2022.

For peer review only - http://bmjopen.bmj.com/site/about/guidelines.xhtml

Data management

Database

Data will be obtained from the electronic medical record and recorded on REDCap, a secure data management system which will be hosted by the University of Cambridge.[30] Data collected will include demographics, physiological parameters, paediatric index of mortality 3 score,[31] treatment received and investigation results from the time of presentation to hospital for the acute illness to discharge.

Statistical analysis plan

Data will be analysed in R.[32] Demographic data will be reported using simple descriptive statistics including mean and standard deviation. For comparisons of demographic data between groups, skewed data using non-parametric tests including Mann-Whitney U test and Kruskal-Wallis test. Normally distributed data will be compared with Student t-tests. Group correlations of quantitative data will be tested with Spearman correlation test.

All TAC detections from NB-BAL samples will be reported using the mean and range of Ct values for each molecular target. This will be compared to growth on culture in cfu/ml or Ct value for routine qPCR testing. A positive TAC result will be considered a Ct of 32 or below, which was found to correlate with microbiology culture thresholds in a previous investigation.[22]

Time to reportable results will be recorded as the time taken from collection time of the sample, which will be entered by nursing staff into the electronic medical record. The comparison of time to result of TAC versus culture will be assessed with Wilcoxon signed ranks test.

Cytokine results will be analysed using a support-vector machine approach to explore profiles of different infections.

Ethics and dissemination

Ethics and registration

This study is registered with clinicaltrials.gov (NCT04233268 – first posted 18/01/2020) and registered via the Integrated Research Application System (IRAS 277039). The study is

For peer review only

jointly sponsored by Cambridge University Hospitals NHS Foundation Trust and the University of Cambridge. The study was approved by the Yorkshire and the Humber – Bradford Leeds Research Ethics Committee on 26/03/2020 (REC reference 20/YH/0089). The amended, current version of the protocol (8.0) was approved by the REC on 02/07/2021. Amendments to this study have primarily been a result of the SARS-CoV-2 pandemic. Additional work packages investigating SARS-CoV-2 infection in children under the overarching RASCALS project are subject to separate protocol and analysis that are not outlined in this paper.

Protocol amendments will be communicated to participants when changes are made that result in a change in procedures for the individual. Participants will be pseudonymised via creation of a sequential study ID.

STARD checklist

A Standards of Reporting of Diagnostic Accuracy Studies (STARD) checklist has been completed (Appendix H).

Appendices

Appendix A: Patient information sheet

Appendix B: Patient consent form

Appendix C: NB-BAL sampling procedure

Appendix D: NB-BAL sampling procedure modifications for COVID-19

Appendix E: Consultant survey

Appendix F: Staff focus group information sheet

Appendix G: Staff focus group consent form

Appendix H: STARD checklist of recommendations for interventional trials

Contributorship statement:

JC, MDC, ACM, VN, MET, SB, and NP conceived the study design; JC, MC, ACM, VN, MET, SB and NP designed the study protocol; JC, IK, MDC, FK, JB-S, MM, and SB developed laboratory methods for the study. JC, DW, ED, SA, DI developed sampling procedure protocol; JC, IK, FK, ACM, RB, TG, ZZ, SB and NP developed the statistical analysis plan; JC, IK, MDC, FK, DW, ED, ACM, VN, RB, TG, SA, ZZ, SB, NP drafted this manuscript.

For peer review only

Competing interests: MDC is the inventor on a patent held by the Secretary of State for Health (UK government) EP2788503, which covers some of the genetic sequences used in this study. VN is a founder, director, and shareholder in Cambridge Infection Diagnostics Ltd (CID Ltd) which is a commercial company aimed at developing molecular diagnostics in infection and antimicrobial and AMR stewardship. ACM, SB, and GD are members of the Scientific Advisory Board of Cambridge Infection Diagnostics Ltd (CID Ltd). TG has received a research grant from Shionogi. All other authors declare no conflict of interest.

Funding: This project is funded by the Addenbrooke's Charitable Trust, Cambridge University Hospitals [900240] (JC, NP, MET, SB) which will provide funding for consumables, 16S rRNA gene sequencing of samples and inflammatory profiling; in addition to the NIHR Cambridge Biomedical Research Centre. The authors also receive support from the Gates Cambridge Trust [OPP1144] (JC); the Academy of Medical Sciences (MET); Wellcome Trust [215515] (SB); Wellcome Trust Clinical Research Career Development Fellowship [WT 2055214/Z/16/Z] (ACM) MRC Clinician Scientist Fellowship [MR/V006118/1] (ACM); and Action Medical Research (NP, SB, MET).

This work was supported, in whole or in part, by the Bill & Melinda Gates Foundation [OPP1144]. Under the grant conditions of the Foundation, a Creative Commons Attribution 4.0 Generic License has already been assigned to the Author Accepted Manuscript version that might arise from this submission.

Data sharing plan: The research team will have access to the final study database. On completion of the project this data will be made available via a data sharing platform (Open Science Framework) and 16S rRNA gene sequencing data will be shared via the European Nucleotide Archive.[33] The project will be reported in peer-reviewed journals and international conferences.

References

1. Global Burden of Disease Collaborators. Global, regional, and national age-sex specific mortality for 264 causes of death, 1980-2016: a systematic analysis for the Global Burden of Disease Study 2016. *Lancet (London, England)* **390**, 1151–1210 (2017).

For peer review only

2. Paediatric Intensive Care Audit Network. *Paediatric Intensive Care Audit Network Annual Report 2019*. https://www.picanet.org.uk/wp-content/uploads/sites/25/2019/12/PICANet_2019_Annual_Report_Tables_and_Figures_v1.0.pdf (2019).
3. Kumar, A. *et al.* Duration of Hypotension before Initiation of Effective Antimicrobial Therapy Is the Critical Determinant of Survival in Human Septic Shock. *Crit. Care Med.* **34**, 1589–1596 (2006).
4. da Silva, P. S. L., de Aguiar, V. E., de Carvalho, W. B. & Machado Fonseca, M. C. Value of clinical pulmonary infection score in critically ill children as a surrogate for diagnosis of ventilator-associated pneumonia. *J. Crit. Care* **29**, 545–550 (2014).
5. Sachdev, A. *et al.* Clinical Pulmonary Infection Score to diagnose ventilator-associated pneumonia in children. *Indian Pediatr.* **48**, 949–954 (2011).
6. Stocker, M. *et al.* Antibiotic surveillance on a paediatric intensive care unit: easy attainable strategy at low costs and resources. *BMC Pediatr.* **12**, 196 (2012).
7. Byington, C. L. *et al.* The Effect of Rapid Respiratory Viral Diagnostic Testing on Antibiotic Use in a Children's Hospital. *JAMA Pediatr.* **156**, 1230–1234 (2002).
8. Whiley, H. & Taylor, M. Legionella detection by culture and qPCR: Comparing apples and oranges. *Crit. Rev. Microbiol.* **42**, 65–74 (2016).
9. Daxboeck, F., Krause, R. & Wenisch, C. Laboratory diagnosis of Mycoplasma pneumoniae infection. *Clin. Microbiol. Infect.* **9**, 263–273 (2003).
10. Maja, K. *et al.* Application of TaqMan Low-Density Arrays for Simultaneous Detection of Multiple Respiratory Pathogens . *J. Clin. Microbiol.* **49**, 2175–2182 (2011).
11. Papan, C. *et al.* Assessment of the multiplex PCR-based assay Unyvero pneumonia application for detection of bacterial pathogens and antibiotic resistance genes in children and neonates. *Infection* **46**, 189–196 (2018).
12. Li, J. *et al.* Rapid detection of respiratory organisms with the FilmArray respiratory panel in a large children's hospital in China. *BMC Infect. Dis.* **18**, 510 (2018).
13. Leber, A. L. *et al.* Multicenter Evaluation of BioFire FilmArray Respiratory Panel 2 for Detection of Viruses and Bacteria in Nasopharyngeal Swab Samples. *J. Clin. Microbiol.* **56**, e01945-17 (2018).
14. Jones, N. K. *et al.* Evaluating the use of a 22-pathogen TaqMan array card for rapid diagnosis of respiratory pathogens in intensive care. *J. Med. Microbiol.* **69**, 971–978 (2020).

For peer review only - http://bmjopen.bmj.com/site/about/guidelines.xhtml

15. Wolff, B. J. *et al.* Improved Detection of Respiratory Pathogens by Use of High-Quality Sputum with TaqMan Array Card Technology. *J. Clin. Microbiol.* **55**, 110 LP – 121 (2017).
16. Mansour, M. G. E. & Bendary, S. Hospital-acquired pneumonia in critically ill children: Incidence, risk factors, outcome and diagnosis with insight on the novel diagnostic technique of multiplex polymerase chain reaction. *Egypt. J. Med. Hum. Genet.* **13**, 99–105 (2012).
17. Goodman, D. *et al.* Challenges in the diagnosis of paediatric pneumonia in intervention field trials: recommendations from a pneumonia field trial working group. *Lancet Respir. Med.* **7**, 1068–1083 (2019).
18. Foglia, E., Meier, M. D. & Elward, A. Ventilator-associated pneumonia in neonatal and pediatric intensive care unit patients. *Clin. Microbiol. Rev.* **20**, 409–425 (2007).
19. Clark, J. *et al.* Low diagnostic yield and time to diagnostic confirmation results in prolonged use of antimicrobials in critically ill children. *Wellcome Open Res.* **6**, 119 (2021).
20. Kane, S. Sample size calculator. *ClinCalc* (2021).
21. Qiagen. *EZ1 Virus Handbook.* https://www.qiagen.com/us/resources/download.aspx?id=ca13c92a-4b1b-4ced-9796-b0414a166803&lang=en (2010).
22. Navapurkar, V. *et al.* Development and implementation of a customised rapid syndromic diagnostic test for severe pneumonia. *medRxiv* 2020.06.02.20118489 (2020) doi:10.1101/2020.06.02.20118489.
23. Public Health England. *UK Standards for Microbiology Investigations: Investigation of bronchoalveolar lavage, sputum and associated specimens*. (2019).
24. EUCAST. *EUCAST disk diffusion method*. (2021).
25. Wood, D. E., Lu, J. & Langmead, B. Improved metagenomic analysis with Kraken 2. *Genome Biol.* **20**, 257 (2019).
26. Howie, S. R. C. Blood sample volumes in child health research: review of safe limits. *Bull. World Health Organ.* **89**, 46–53 (2011).
27. Bio-Rad. *Bio-Plex Pro Human Cytokine Assays - Instruction Manual*. (2021).
28. QRS International Pty Ltd. Nvivo qualitvative data analysis software. (2018).
29. Tume, L. *et al.* Research Priorities for U.K. Pediatric Critical Care in 2019: Healthcare Professionals' and Parents' Perspectives. *Pediatr Crit Care Med* **22**, e294–e301 (2021).

For peer review only

30. Harris, P. A. *et al.* Research electronic data capture (REDCap)—A metadata-driven methodology and workflow process for providing translational research informatics support. *J. Biomed. Inform.* **42**, 377–381 (2009).

31. Straney, L. *et al.* Paediatric index of mortality 3: an updated model for predicting mortality in pediatric intensive care*. *Pediatr. Crit. Care Med.* **14**, 673–681 (2013).

32. RStudio Team. RStudio: Integrated Development for R. (2019).

33. European Bioinformatics Institute - European Molecular Biology Laboratory. European Nucleotide Archive. https://www.ebi.ac.uk (2021).

Figure 1: Power calculation

Calculation generated with ClinCalc[20]

Figure 2: Workflow

LRTI: Lower respiratory tract infection; qPCR: quantitative polymerase chain reaction; TAC: Taqman Array Card

For peer review only

For peer review only - http://bmjopen.bmj.com/site/about/guidelines.xhtml

$$N = \frac{p_0 q_0 \left\{ z_{1-\alpha/2} + z_{1-\beta} \sqrt{\frac{p_1 q_1}{p_0 q_0}} \right\}^2}{(p_1 - p_0)^2}$$

$$q_0 = 1 - p_0$$

$$q_1 = 1 - p_1$$

$$N = \frac{0.22 * 0.78 \left\{ 1.96 + 0.84 \sqrt{\frac{0.352*0.648}{0.22*0.78}} \right\}^2}{(0.352 - 0.22)^2}$$

$$N = 85$$

p_0 = proportion (incidence) of population
p_1 = proportion (incidence) of study group
N = sample size for study group
α = probability of type I error (usually 0.05)
β = probability of type II error (usually 0.2)
z = critical Z value for a given α or β

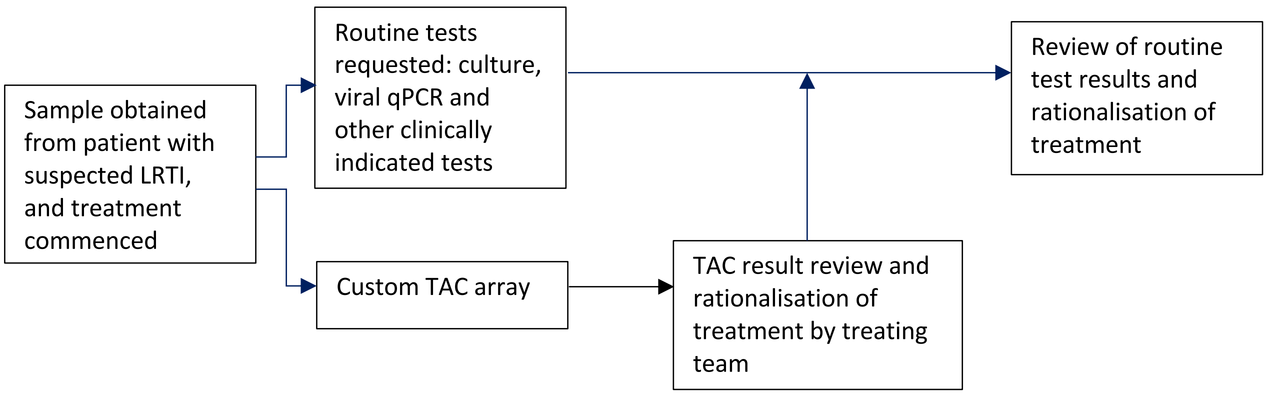

For peer review only

Department of Paediatric Intensive Care

Dr Roddy O'Donnell
Dr Rosalie Campbell
Dr David Inwald
Dr Shruti Agrawal
Dr Nazima Pathan
Dr Girish Neelegowda
Dr Riaz Kayani

Cambridge University Hospitals NHS
NHS Foundation Trust

Addenbrooke's Hospital
Hills Road
Cambridge CB2 0QQ

Switchboard: 01223 245151
www.addenbrookes.org.uk

Rapid Assay for Sick Children with Acute Lung infection Study
Invitation and Information Sheet – Parent/Carer of critically ill child

RASCALS

Rapid Assay for Sick Children with Acute Lung infection Study

Thank you for taking the time to consider having your child participate in 'RASCALS' – the Rapid Assay for Sick Children with Acute Lung infection Study.

Before you decide whether to have your child participate it is important for you to understand why the research is being done and what it will involve. Please take time to read the following information carefully and discuss it with others if you wish. Do ask us if there is anything that is not clear or if you would like more information. Take time to decide whether or not you wish to take part.

Key points about the study

- We are investigating bacteria and viruses that cause chest infection in children. In this study we will be using your child's breathing tube secretions, swabs, blood and faeces to help us improve the way we diagnose these infections.
- All the children in the study will be tested for Coronavirus 2019 (COVID-19), we will let you know the results.
- One of the rapid bacteria and virus tests is something we're trying for the first time. We will be doing this test using what is left over from your child's breathing tube sample which is needed even if they are not in the study. We will share the results of this test with you, and your child's doctors. It is possible these test results could help your child's doctors make decisions regarding your child's antibiotic treatment. We will ask them via a survey if the test was helpful.
- If your child is strongly suspected to have COVID-19 we will ask for your help collecting weekly throat swabs and either a swab of the bottom or faecal sample. This will continue for 4 weeks and will conclude with a survey about your child's symptoms.
- If at any point you no longer wish for your child to take part let your nurses and doctors know. Your child will receive high quality care during their stay in the paediatric intensive care unit whether or not they take part.

Key points about your child's information

For peer review only - http://bmjopen.bmj.com/site/about/guidelines.xhtml

- In this research study we will use information from your child's medical records. We will only use information that we need for the research study. We will let very few people know your name or contact details, and only if they really need it for this study.
- Everyone involved in this study will keep your data safe and secure. We will also follow all privacy rules. At the end of the study we will save some of the data in case we need to check it and for future research.
- We will make sure no-one can work out who you are from the reports we write. The following information pack tells you more about this.

Kind regards,
The Paediatric Intensive Care Unit Research Team

RASCALS researchers: Dr Nazima Pathan, Dr John Clark, Dr Iain Kean, Dr Estée Török, Prof Gordon Dougan, Prof Stephen Baker, Dr Vilas Navapurkar, Ms Esther Daubney & Ms Deborah White.
Phone: 01223 336883
Email: np409@medschl.cam.ac.uk

*This study has been co-sponsored by Cambridge University Hospitals NHS Foundation Trust and the University of Cambridge. Any reference to 'we' or 'us' in these documents refers to these entities.

For peer review only - http://bmjopen.bmj.com/site/about/guidelines.xhtml

Carer Information: Rapid Assay for Sick Children with Acute Lung infection Study

What is the purpose of this study?

The Department of Paediatric Intensive Care at Addenbrooke's Hospital is actively involved in research in children who are critically ill. We want to do research that helps us develop better ways of diagnosing and treating children admitted to our unit. Our research nurses and doctors would like to talk to you about the work that we are doing.

In this specific study, we are interested in COVID-19 and in the diagnosis and treatment of lung infections. Currently, the best tests we have available for diagnosing lung infections are slow, and often can't detect the bugs that have caused infection. We are trying a new rapid infection test to see if it might be faster and able to detect these bugs more often. We will share the results of the test with you and your child's doctors. We will ask your doctors whether the test had any impact on their decisions on the type and duration of antibiotics they prescribe. Your child will still have all their routine tests done as well.

In addition to the rapid infection test, we will be using the samples we collect to test for new markers of infection and COVID-19.

Why has my child been chosen?

We are studying children that are unwell in the Paediatric Intensive Care Unit that have potential COVID-19 and/or lung infection requiring intubation and ventilation (a breathing tube down the windpipe and the support of a breathing machine). We we are aiming to improve tests and obtain more information about COVID-19.

Who is organising the study?

The study is being organised by the Department of Paediatric Intensive Care by Dr Nazima Pathan and Dr John Clark, here at Addenbrooke's Hospital. This work is supervised by the University of Cambridge. No specific payments will be made to any of the staff involved for including your child in this study other than their normal salary.

Does my child have to take part?

It is up to you whether or not your child should take part. If you chose for your child to participate you will be given this information sheet to keep and be asked to sign a consent form. If you decide to have your child take part, you are still free to withdraw this decision at any time and without giving a reason. A decision to withdraw or a decision not to take part will not affect the standard of care your child will receive.

What will happen to my child if they take part?

Your child will receive infection tests whilst we can care for them in intensive care, and the 4 weeks that follow if they are strongly suspected to have COVID-19. The first set of tests will be done by hospital staff. If your child is well enough to go home in the following 4 weeks we will ask for your help taking throat swabs and faecal samples or swabs of the bottom. We will provide directions on how to do this and how to post the samples back to us. A chart of the tests that will be done is on the following page.

For peer review only - http://bmjopen.bmj.com/site/about/guidelines.xhtml

When	What test?		What for?	Will I find out the result?
As soon as possible	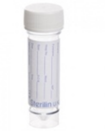	Breathing tube sample	Bacteria and viruses	Yes
			Coronavirus 2019	Yes
			Markers of infection and antibiotic resistance	No – this will be for research only
	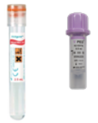	Blood	Markers of infection	No – this will be for research only
			Coronavirus 2019	Yes
	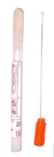	Nasopharyngeal swab (swab at the back of the nose) or nasopharyngeal aspirate (secretions from back of the nose)	Coronavirus 2019	Yes
	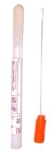	Throat swab	Coronavirus 2019	Yes
	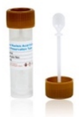	Faecal sample or rectal swab	Coronavirus 2019	Yes*
			Markers of infection and antibiotic resistance	No – this will be for research only
If there is strong clinical suspicion or tests confirm Coronavirus 2019, the following samples will be obtained.				
2-4 days later	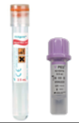	Blood	Markers of infection and antibiotic resistance	No – this will be for research only
			Coronavirus 2019	Yes*
5-7 days later	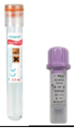	Blood	Markers of infection and antibiotic resistance	No – this will be for research only
			Coronavirus 2019	Yes*
Weekly for 4 weeks	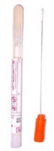	Throat swab	Coronavirus 2019	Yes*
	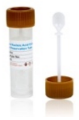	Faecal sample or rectal swab	Coronavirus 2019	Yes*
			Markers of infection and antibiotic resistance	No – this will be for research only

* The first set of nose and throat swabs we do will be processed quickly, however COVID-19 tests on other samples may take multiple weeks given resources available in the health service.

None of the samples we obtain will result in extra procedures for your child. The blood sample amount will be within safe limits according to the weight of your child, which we will take from their central line. We will not need to use any extra needles to take this blood.

For peer review only
For peer review only - http://bmjopen.bmj.com/site/about/guidelines.xhtml

The rapid bacteria and virus test we are trying has not been used before in sick children, therefore it will be in addition to our normal infection tests. We will let you and your child's doctors know the results of this new test. We will ask the doctors whether this test made any difference to the antibiotic treatment they prescribe. This will be via a short questionnaire we have developed.

All the samples we take will be frozen once we have finished our study and stored for up to 10 years. This means that with ethics committee approval they could be used for other related studies.

Why have you saved samples prior to asking me?
To best detect infection tests need to be done as early as possible. The tests don't work as well once more and more doses of antibiotics are given. We put some of the samples we need to take aside so this study would not mean we were doing extra procedures on your child. Whether we use these samples for our study is your decision.

What do I and my child have to do?
No specific restrictions need to be placed upon your child as a consequence of participating in the study. Your child would continue with any regular medication.

Aside from taking and posting the samples mentioned earlier, we will ask you to complete a short online survey. We will send you a link to this survey via your email address. It will contain questions about your child's symptoms and how long they lasted, in addition to whether your child was given any antibiotics once they left the intensive care unit. This data will go directly to a secure online database only accessible by the research team.

What are the possible risks / side effects of taking part?
Taking extra blood and secretion samples will not mean any significant extra risk or harm to your child. Taking the secretion samples from the breathing tube does not cause additional harm to your child's lungs.

What are the possible benefits of taking part?
The study is aimed at improving our ability to diagnose the cause of lung infections and identifying the best treatment early.

Being part of this study means additional information will be obtained regarding possible causes of infection in your child. It is not known whether the results will have any benefits / implications for your child directly. However, by taking part you may contribute to helping children and adults suffering from chest infections in the future.

The COVID-19 tests may help you decide how long you keep your child and your family isolated. We will discuss the results of the tests with your child's medical team to provide you with the best information about what you should do.

What if new information becomes available?
If, during the course of this study, new information becomes available suggesting that in order to give your child the best possible care for your child's condition and treatment should be changed, then we will make those changes. Changes such as these will not affect your child's participation in this study.

For peer review only - http://bmjopen.bmj.com/site/about/guidelines.xhtml

What happens at the end of the study?
Any samples that your child has provided up until the end of the study will be stored by the research team and pending ethical approval may be used in another project. If we do not make a subsequent application for ethical approval in the required timeframe or if such an application is refused, then your child's samples will be destroyed.

How will we use information about your child?
We will need to use information from your child's medical records for this research project. This information will include your child's name, medical record number and date of birth. People will use this information to do the research or to check your records to make sure that the research is being done properly. This information containing identifiers will be retained for up to 15 years.

People who do not need to know who your child is will not be able to see these personal details. Your child's data will have a code number instead. We will keep all information about your child safe and secure.

Once we have finished the study, we will keep some of the data so we can check the results. We will write our reports in a way that no-one can work out that you took part in the study.

At the end of the study the data will become "open data". This means that it will be stored in an online database so that it is publicly available. This data will be thoroughly anonymised by removing any personal information that could identify your child, such as names and addresses, before submission. This process is integral to the research process as it allows other researchers to verify results and avoid duplicating research. Data are made available on a website, free of charge, to anyone interested in the research, or who wishes to conduct their own analysis of the data. We would therefore have no control over how these data are used.

Will my child's GP be informed?
With your agreement, your child's GP will be informed should you consent to their participation in the study.

What are your choices about how your information is used?
- You can stop your child being part of the study at any time, without giving a reason, but we will keep information about your child that we already have.
- We need to manage your child's records in specific ways for the research to be reliable. This means that we won't be able to let you see or change the data we hold about your child.
- If you agree for your child to take part in this study, you will have the option to take part in future research using your data saved from this study. This data will be kept within the University of Cambridge Department of Paediatrics for up to 15 years following the conclusion of the study.

Where can you find out more about how your child's information is used?
You can find out more about how we use your information
- at www.hra.nhs.uk/information-about-patients/
- our leaflet available from **www.hra.nhs.uk/patientdataandresearch**
- by asking one of the research team

For peer review only - http://bmjopen.bmj.com/site/about/guidelines.xhtml

- by sending an email to np409@medschl.cam.ac.uk or
- by ringing us on 01223 336883

What if there is a problem?
If, when taking part in the project, your child is harmed as a consequence of either the management or conduct of the project then the NHS indemnity scheme will apply. If your child is harmed as a consequence of a problem with the design of the project, then this will be covered by insurance through the University of Cambridge's Clinical Trials policy. If your child is harmed due to someone's negligence, then you may have grounds for a legal action, but you may have to pay for it. Regardless of this, if you wish to complain, or have any concerns about any aspect of the way you have been approached or treated during the course of this study, you can contact the National Health Service Patient Advice and Liaison Service (PALS) or the Independent Complaints Advocacy Service (ICAS). Contact details are as follows:

Patient Advice and Liaison Service (PALS)
Box 53, Cambridge University Hospitals NHS Foundation Trust,
Addenbrooke's Hospital, Hills Road,
Cambridge, CB2 0QQ
Tel: 01223 216756, Fax: 01223 256170

Cambridgeshire, Norfolk and Suffolk ICAS
POhWER,
PO Box 14043
Birmingham
B6 9BL
Tel: 0300 456 2370, Fax: 0300 456 2365

What if we want to withdraw from the study?
You may withdraw your child from the study at any time without giving a reason. A decision to withdraw will not affect the standard of care you receive. In line with the guidelines from the UK Medical Research Council on the use of biological samples in scientific research, we will treat each of the blood samples which you provide us as a gift / donation. Consequently, even if you decide to withdraw from the study we may (with your permission) continue to retain any / all of the samples which you may have already provided but will discard them if you prefer.
If you wish to withdraw from the study, please let your bedside nurse know and they will get in touch with one of the research nurses or doctors.

Who has reviewed the study?
All research in the NHS is looked at by independent group of people, called a Research Ethics Committee, to protect your interests. This study has been reviewed by the research advisory committee of NHS Cambridge University Hospitals NHS Foundation Trust and the research ethics committee of Bradford-Leeds.

Thank you for considering your child's participation in this research project.

For peer review only - http://bmjopen.bmj.com/site/about/guidelines.xhtml

Department of Paediatric Intensive Care

Dr Roddy O'Donnell
Dr Rosalie Campbell
Dr David Inwald
Dr Shruti Agrawal
Dr Nazima Pathan
Dr Girish Neelegowda
Dr Riaz Kayani

Cambridge University Hospitals NHS
NHS Foundation Trust

Addenbrooke's Hospital
Hills Road
Cambridge CB2 0QQ

Switchboard: 01223 245151
www.addenbrookes.org.uk

Rapid Assay for Sick Children with Acute Lung infection Study
Consent Form – Parent/Carer of critically ill child

Thank you for taking the time to consider having your child participate in 'RASCALS' – the Rapid Assay for Sick Children with Acute Lung infection Study and reading the information sheets.

If you would like your child to take part in the study, please read and sign the following forms.

We will provide you with one copy of this form and retain a second copy securely in our research department at the hospital.

Kind regards,

The Paediatric Intensive Care Unit Research Team
RASCALS researchers: Dr Nazima Pathan, Dr John Clark, Dr Iain Kean, Dr Estée Török, Prof Gordon Dougan, Prof Stephen Baker, Dr Vilas Navapurkar, Ms Esther Daubney & Ms Deborah White.
Phone: 01223 336883
Email: np409@medschl.cam.ac.uk

For peer review only - http://bmjopen.bmj.com/site/about/guidelines.xhtml

**Patient identification number**:		**Initials**
1.	I have read the information sheet on this project, dated ___/___/____ (Version________) and have been given a copy to keep. I have been able to ask questions about the project and I understand why the research is being done and any risks involved.	
2.	I give my permission for a breathing tube secretion sample, blood sample, swabs and faecal samples to be used for the purposes of this study. I understand that this blood sample did not result in any additional needle procedures for my child for the purposes of the study.	
3.	I understand that I may be asked to assist with obtaining swabs and stool specimens from my child if they are well enough to go home in the next 4 weeks if they have strongly suspected or confirmed Coronavirus disease 2019.	
4.	I understand how the samples will be collected and that giving samples for this research is voluntary. I understand I can withdraw consent for my child's participation in the study at any time, without giving a reason and without my child's medical treatment or legal rights being affected.	
5.	I understand that the samples taken could be used for other research studies, if approved by an ethics committee, for up to 10 years after the study.	
6.	I understand that the research team will email me a survey to complete via a secure online platform in 4 weeks to ask about my child's symptoms, and treatment.	
7.	I give permission for someone from the research team to look at my child's medical records to get information on my child's illness and related medical history where it is relevant to the research.	
8.	I know how to contact the research team if I need to, and how to get information about the results of the research.	
9.	I understand that any information collected about my child as part of this study will be stored securely in line with current NHS and University guidelines. I understand that only members of the clinical project team will have access to my child's identifiable information; all others analysing my data will only have access to anonymised data. I understand that my child's data will be retained for 15 years.	
10.	I understand that the information collected about my child will be used to support other research in the future, and may be shared anonymously with other academic and commercial researchers external to the project within the UK and beyond	
11.	I consent to my child taking part in this study	

For peer review only - http://bmjopen.bmj.com/site/about/guidelines.xhtml

Written consent

Carer

Name (Capitals):______________________________

Signature:______________________________ Date: dd/mm/yyyy

Relationship to patient: ______________________________

Person obtaining consent

Name (Capitals):______________________________

Signature:______________________________ Date: dd/mm/yyyy

Role: ______________________________

Electronic consent

Carer name (Capitals):______________________________

Person obtaining consent

Name (Capitals):______________________________

Signature:______________________________ Date: dd/mm/yyyy

Role: ______________________________

Verbal consent

Carer name (Capitals):______________________________

Person obtaining consent

Name (Capitals):______________________________

Signature:______________________________ Date: dd/mm/yyyy

Role: ______________________________

Witness to verbal consent

Name (Capitals):______________________________

Signature:______________________________ Date: dd/mm/yyyy

Role: ______________________________

Primary investigator countersign for electronic and unwitnessed verbal consent

Dr Nazima Pathan ______________________________ Date: dd/mm/yyyy

For peer review only - http://bmjopen.bmj.com/site/about/guidelines.xhtml

For peer review only

Paediatric intensive care unit (PICU)

Division E

Policy

Non-bronchoscopic bronchoalveolar lavage sampling in children

Key messages

- Non-bronchoscopic bronchoalveolar lavage (NB-BAL) can be used to obtain lower respiratory tract secretions to assist in the diagnosis and treatment of lower respiratory tract infection
- NB-BAL samples have a higher yield than endotracheal samples alone
- NB-BAL sampling should only be undertaken after discussion with the PICU consultant that the patient is safe for the procedure
- The procedure should only be undertaken when a senior PICU nurse (in addition to the bedside nurse) and PICU doctor are available on the unit to assist if required

For peer review only

1 Scope

Paediatric intensive care unit – Addenbrooke's Hospital

2 Purpose

This document is to assist staff in understanding the procedure of NB-BAL sampling, and standardise the way samples are being obtained via NB-BAL in the PICU

3 Definitions

ETT: Endotracheal tube
NB-BAL: Non-bronchoscopic bronchoalveolar lavage
PICU: Paediatric intensive care unit

4 Introduction

Non-bronchosopic bronchoalveolar lavage (NB-BAL) may be also referred to as 'blind' bronchoalveolar lavage. It is a method of sampling secretions found in the lower respiratory tract, for children that are requiring invasive ventilation in intensive care for suspected or confirmed infection. Infection may be suspected based on clinical findings or chest x-ray changes.

NB-BAL has a sensitivity of up to 50-84% and specificity up to 50-95%, for detecting microorganisms in ventilator associated pneumonia.[123] There is higher

For peer review only - http://bmjopen.bmj.com/site/about/guidelines.xhtml

yield than endotracheal tube (ETT) sampling alone.[1] Advantages include reduced cost in comparison to bronchoscopic bronchoalveolar lavage, less expertise required and the ability to undertake the procedure on smaller children that may not have an airway large enough for bronchoscopy sampling to be undertaken.

Samples of lower respiratory tract secretions can assist in the management of children by way of identifying pathogens causing underlying lower respiratory tract infection. This may then allow antimicrobial therapy to be directed towards the causative bacteria, or ceased if the illness has no underlying bacterial aetiology.

Complications are uncommon in children,[1] and can be prevented or minimised with careful prior assessment of patients.

5 Responsibilities

- PICU consultant – advise whether patient is safe to undergo NB-BAL
- Senior PICU nurse and PICU doctor – maintain availability within the PICU whilst patient is undergoing NB-BAL, in case additional intervention is required
- NB-BAL proceduralist (physiotherapist/nurse/PICU doctor) – undergo procedure as per policy

6 Procedure

6.1 Indications

1. To obtain specimens for microbiology and virology that can assist in identifying pathogens causing lower respiratory tract infection

6.2 Contraindications

- FiO2 >0.85[4]
- PEEP >10cm H_2O[4]
- Pneumothorax
- Raised intracranial pressure
- Acute pulmonary oedema
- Active seizures
- Severe acute asthma
- Thrombocytopaenia[4] with platelets $<50 \times 10^9$ L^{-1}

6.3 Precautions (discuss with PICU consultant)

- Cardiovascular instability

For peer review only - http://bmjopen.bmj.com/site/about/guidelines.xhtml

For peer review only

- Deranged clotting
- Pulmonary hypertension
- Pulmonary haemorrhage
- High ventilation pressures required

6.4 Complications

- Hypoxia[145]
- Transient reduction in tidal volume[4]
- Vagal stimulation[5]
- Pneumothorax
- Mucosal trauma
- Increased intracranial pressure
- Haemodynamic instability[5]
- Arrhythmia[3]
- Bronchospasm[3]
- Haemoptysis[6]
- Haemorrhage

6.5 Pre-procedure preparation

This procedure should only be performed where senior nursing staff and a member of medical staff are present in the PICU. These staff should be notified prior to the procedure taking place.

Consider whether additional sedation will be required for the child to facilitate the procedure. This should be discussed with nursing and medical staff.

Review the most recent chest x-ray - note the position of the tip of the ETT and distance from the carina. The suction catheter will be advanced to the level of the carina i.e. depth of ETT + distance from tip of ETT to carina

6.6 Equipment required

- T-piece anaesthetic circuit
- Bag valve mask available
- Alternative suction device eg. Yankauer suction tip available
- End-tidal CO_2, oxygen saturation and heart rate monitoring, blood pressure monitoring
- Dressing pack with sterile gloves
- Sterile suction catheter (Bicakcilar, suction catheter ideal tip, REF 190 1085 1)
- 2x Tube suction connector with female Luer-lock port (Vycon REF 801.00)
- 1x Male:Male Luer-lock connector (Vycon REF 893.00)
- Sputum trap
- 10mL, 5mL or 2mL syringes (dependent on patient size)
- 3 way tap
- 0.9% sodium chloride at room temperature

For peer review only - http://bmjopen.bmj.com/site/about/guidelines.xhtml

Paediatric intensive care unit (PICU)

Division E

6.7 NB-BAL procedure

6.7.1 Preparation

1. Discuss procedure with legal guardian/care giver where possible, and the child if requiring minimal sedation.
2. Inform nursing and medical staff plan to undertake the procedure and discuss whether additional sedation is required
3. Wash hands and put on face shield, gloves and apron, in addition to any patient specific infection control attire. Assemble equipment as shown in image 1.
4. Draw up 1ml/kg of 0.9% sodium chloride, to a maximum of 10mL in a syringe, leaving an additional 2mL air in the syringe. Connect to 3 way tap and prime suction catheter.
5. Ensure 3 way tap is open to saline flush but closed to suction system
6. Connect suction circuit, with appropriate suction pressure for age of child
7. Review observations and ventilation settings, and confirm with nursing staff safety to proceed with sedation (if indicated) and NB-BAL
8. Pre-oxygenate for 2 minutes[4] (unless there is a cardiac contraindication)
9. Auscultate lung fields

Image 1: Assembled equipment for non-bronchoscopic bronchoalveolar lavage

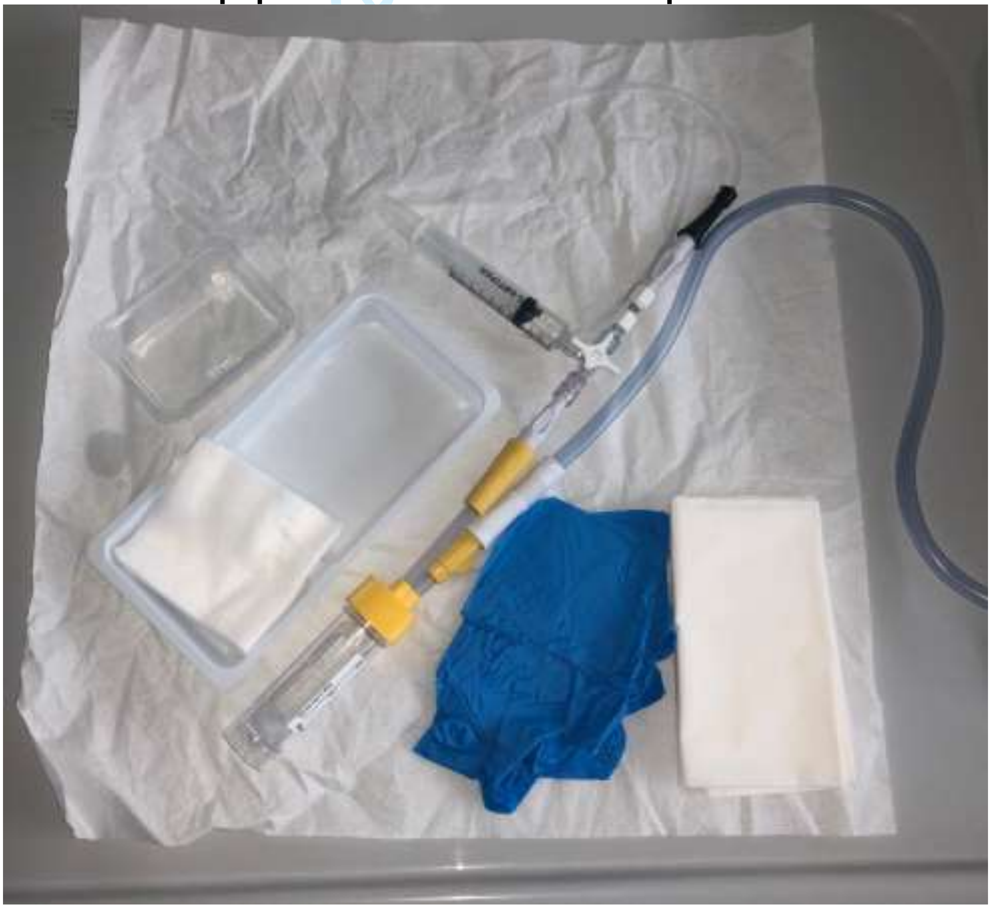

For peer review only - http://bmjopen.bmj.com/site/about/guidelines.xhtml

Paediatric intensive care unit (PICU)

Division E

6.7.2 Procedure

1. Request nursing team to administer sedation agent if this is required
2. Position child. If there is unilateral lung pathology, position the head to the contra-lateral side. i.e. If there is right side lung consolidation, position the head to the left.
3. Provide manual inflations via t-piece
4. Advance the suction catheter to the level of the carina
5. Instill saline and the 2mL of air contained in the syringe. Volume instilled is at the discretion of the proceduralist, as this may vary depending on quality of the secretions but should not exceed volume described in 'preparation'.
6. Open 3 way tap to suction, delivering suction for 2-4 seconds, withdrawing the catheter by maximum 2cm. Ensure sputum trap remains in the upright position.[7]
7. Close 3 way tap to suction and withdraw catheter

6.7.3 Post-procedure

1. Reconnect to mechanical ventilation
2. Label and send sputum sample for microbiology, cultures and sensitivities

7 Monitoring compliance with and the effectiveness of this document

Annual inspection of NB-BAL procedure whilst undertaken on PICU to review appropriateness of protocol for current equipment available on the unit and needs of staff. This will be the responsibility of PICU consultant medical staff and discussed at the following unit consultant meetings should modification of the protocol be required.

8 References

1. Khilnani, G. C. *et al.* Comparison of bronchoscopic and non-bronchoscopic techniques for diagnosis of ventilator associated pneumonia. *Indian J. Crit. Care Med.* **15**, 16–23 (2011).
2. Papazian, L. *et al.* Bronchoscopic or blind sampling techniques for the diagnosis of ventilator-associated pneumonia. *Am. J. Respir. Crit. Care Med.* **152**, 1982–1991 (1995).
3. Boots, R. J., Phillips, G. E., George, N. & Faoagali, J. L. Surveillance culture utility and safety using low-volume blind bronchoalveolar lavage in the diagnosis of ventilator-associated pneumonia. *Respirology* **13**, 87–96 (2008).
4. Bonvento, B. *et al.* Non-directed bronchial lavage is a safe method for sampling the respiratory tract in critically ill patient. *J. Intensive Care Soc.* **20**, 175114371881111 (2018).
5. Burmester, M. & Mok, Q. How safe is non-bronchoscopic bronchoalveolar lavage in critically ill mechanically ventilated children? *Intensive Care Med.* **27**, 716–721 (2001).

For peer review only - http://bmjopen.bmj.com/site/about/guidelines.xhtml

6. Gauvin, F. *et al.* Reproducibility of Blind Protected Bronchoalveolar Lavage in Mechanically Ventilated Children. *Am. J. Respir. Crit. Care Med.* **165**, 1618–1623 (2002).
7. Greater Glasgow and Clyde NHS. Bronchoalveolar (blind) lavage (PICU). *GG&C Paediatric Guidelines* (2019). Available at: http://www.clinicalguidelines.scot.nhs.uk/ggc-paediatric-guidelines/ggc-guidelines/respiratory/bronchoalveolar-blind-lavage-picu/. (Accessed: 7th November 2019)

Equality and diversity statement

This document complies with the Cambridge University Hospitals NHS Foundation Trust service equality and diversity statement.

Disclaimer

It is **your** responsibility to check against the electronic library that this printed out copy is the most recent issue of this document.

Document management

Approval:	29 November 2019		
JDTC approval:	N/A		
Owning department:	Paediatric intensive care unit		
Author(s):	John Clark		
Pharmacist:	N/A		
File name:	NB-BAL Version1 November 2019.doc		
Supersedes:	n/a (new document)		
Version number:	1	Review date:	November 2022
Local reference:		Document ID:	101409

For peer review only - http://bmjopen.bmj.com/site/about/guidelines.xhtml

Procedure modifications for lower respiratory lavage sampling on potential COVID-19 patients

Procedural equipment

- T-piece anaesthetic circuit
- Bag valve mask available
- Alternative suction device eg. Yankauer suction tip available
- End-tidal CO_2, oxygen saturation and heart rate monitoring, blood pressure monitoring
- Dressing pack
- In-line suction catheter (if not already connected to patient)
- 1x green tube connector for in-line suction catheter
- 1x Tube suction connector with female Luer-lock port (Vycon REF 801.00)
- 1x Male:Male Luer-lock connector (Vycon REF 893.00)
- Sputum trap
- 10mL or 20mL syringe (dependent on patient size)
- 3 way tap
- 0.9% sodium chloride at room temperature

COVID protective equipment

- Bag to dispose used equipment
- Personal protective equipment
- 2x bags for specimen

Syringe size

- If the patient is <=5kg, use a 10mL syringe
- If the patient is >5kg use a 20mL syringe

Draw up required amount of air and saline in syringe

- Saline: 1mL/kg to maximum 10mL saline
- Air:
  - If the volume of saline is <5mL: draw up 5mL air + (5 – volume of saline in mL)
  - If the volume of saline is >5mL: have 5mL air in the syringe

Procedure

- Assemble equipment, ensuring connections to sputum trap not too tight (2nd line on the connector)
- Close catheter to suction using 3-way tap
- Prime suction catheter with first 5mL of syringe contents (air/saline), holding down the button on the in-line suction catheter
- Advance suction catheter to below the tip of the ETT
- Deliver all of the remaining syringe contents (air + saline), holding down the button the in-line suction catheter
- Switch 3 way tap back to suction, hold down the button on the in-line suction catheter, ensuring the sputum trap remains upright

For peer review only

- Close the 3 way tap to suction and withdraw the suction catheter
- Turn off suction at the wall to reduce potential aerosolisation
- If patient remains stable, only 1 staff member should be in the room to complete final step if possible
- Disconnect the sputum trap and connect the in-line suction catheter back to wall suction. Place universal container lid on the specimen container.
- Dispose of used equipment in bag for highly infectious waste
- Label and package aspirated sample as required for clinical/research purposes as per current guidance

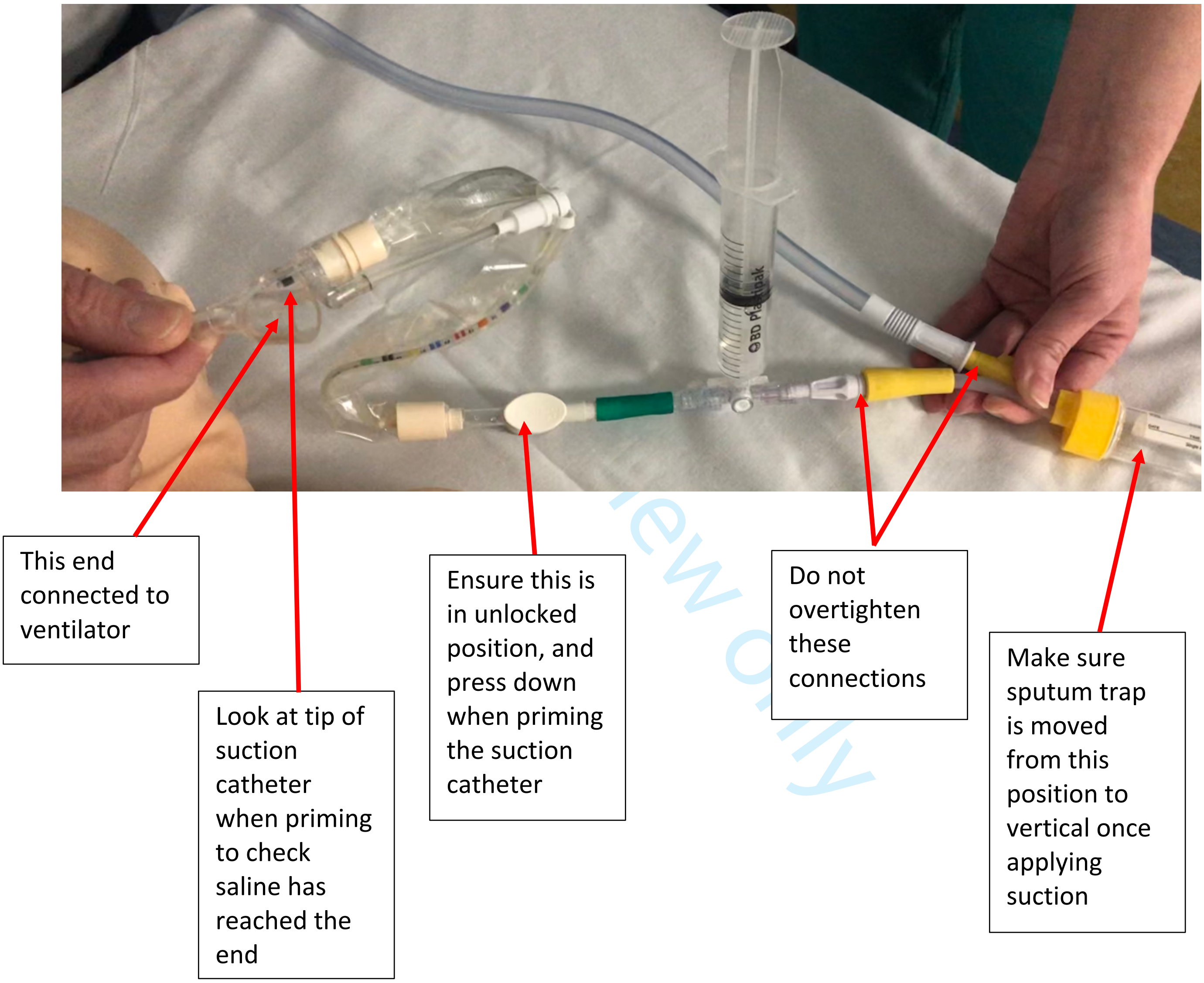

Clinical team survey

Thank you for participating in RASCALS.

Lower respiratory tract infection is a leading cause of admission to paediatric intensive care, and indication for antimicrobial therapy. It is difficult to rationalise antimicrobial therapy given these patients are so unwell, and with current microbiology techniques taking several days to provide a result. Rapid diagnostic tests have the potential to reduce the exposure of our patients to antimicrobials and provide earlier rationalisation of therapy.

We are investigating the impact of a rapid diagnostic test, a Taq-Man array, on clinical practice for ventilated children who are receiving antimicrobial treatment for lower respiratory tract infection. The following survey is to determine whether changes are implemented following Taq-man array results and would appreciate your assistance with this.

We request that you complete this survey at the time the Taq-Man array result becomes available. This survey should take less than 5 minutes. We are incredibly grateful for your time.

Kind regards,

The Paediatric Intensive Care Unit Research Team

RASCALS researchers: Dr Nazima Pathan, Dr John Clark, Dr Iain Kean, Dr Estée Török, Prof Gordon Dougan, Prof Stephen Baker, Dr Vilas Navapurkar, Ms Esther Daubney & Ms Deborah White.

Phone: 01223 336883

Email: np409@medschl.cam.ac.uk

1) Did the Taq-Man result impact on the ongoing antimicrobial type prescribed by the clinical team?	◯ No<br>◯ Yes antimicrobial therapy was de-escalated<br>◯ Yes, antimicrobial therapy was escalated
2) Did the Taq-Man result impact on the clinical team's decision regarding planned duration of antimicrobial therapy?	◯ No<br>◯ Yes, there was a plan for longer duration of antimicrobial therapy<br>◯ Yes, there was a plan for shorter duration of antimicrobial therapy<br>◯ Yes, antimicrobial therapy was ceased
3) Did the Taq-Man result contribute to a change in the patient's isolation status?	◯ No, the test had no impact on isolation status<br>◯ Yes, isolation was ceased due to the Taq-Man result<br>◯ Yes, isolation was continued due to the Taq-Man result
4) If you have any other comments regarding the Taq-Man result, antimicrobial prescription and isolation status please write here	__________________________________

For peer review only - http://bmjopen.bmj.com/site/about/guidelines.xhtml

Department of Paediatric Intensive Care

Dr Roddy O'Donnell
Dr Shruti Agrawal
Dr David Inwald
Dr Riaz Kayani
Dr Girish Neelegowda
Dr Nazima Pathan
Dr Stewart Reid
Dr Francesc Torres

Cambridge University Hospitals NHS
NHS Foundation Trust

Addenbrooke's Hospital
Hills Road
Cambridge CB2 0QQ

Switchboard: 01223 245151
www.addenbrookes.org.uk

Rapid Assay for Sick Children with Acute Lung infection Study
Staff focus group – experiences of TaqMan diagnostic array

RASCALS

Rapid Assay for Sick Children with Acute Lung infection Study

Thank you for taking the time to consider participating in a focus group for 'RASCALS' – the Rapid Assay for Sick Children with Acute Lung infection Study.

Before you decide to participate it is important for you to understand why the research is being done and what it will involve. Please take time to read the following information carefully. Do ask us if there is anything that is not clear or if you would like more information. Take time to decide whether or not you wish to take part.

Kind regards,
The Paediatric Intensive Care Unit Research Team

RASCALS researchers: Dr Nazima Pathan, Dr John Clark, Dr Iain Kean, Dr Estée Török, Prof Gordon Dougan, Prof Stephen Baker, Dr Vilas Navapurkar, Ms Esther Daubney & Ms Deborah White.
Phone: 01223 336883
Email: np409@medschl.cam.ac.uk

*This study has been co-sponsored by Cambridge University Hospitals NHS Foundation Trust and the University of Cambridge. Any reference to 'we' or 'us' in these documents refers to these entities.

For peer review only - http://bmjopen.bmj.com/site/about/guidelines.xhtml

Focus group participant information:
Rapid Assay for Sick Children with Acute Lung infection Study

What is the purpose of this study?
The Department of Paediatric Intensive Care at Addenbrooke's Hospital is actively involved in research in children who are critically ill. We want to do research that helps us develop better ways of diagnosing and treating children admitted to our unit. Our research team would like to talk to you about the work that we are doing.

In the RASCALS project we have been researching the lung microbiome in critical care, rapid diagnostic tools for pneumonia and biomarkers of COVID-19 in children. In this focus group however, we are specifically interested in staff experiences of the TaqMan respiratory diagnostic array, which has been trialled on the unit since April 2020.

Why have I been asked to participate?
We are interested in capturing the experiences of staff currently working on the PICU in relation to the TaqMan diagnostic array. We are the first team in the world to integrate this test into a paediatric clinical service – we would like to capture this experience to drive future research and implementation of this test.

Who is organising the study?
The study is being organised by the Department of Paediatric Intensive Care by Dr Nazima Pathan and Dr John Clark, here at Addenbrooke's Hospital. This work is supervised by the University of Cambridge. No specific payments will be made to any of the staff involved for including your child in this study other than their normal salary.

Does I have to take part?
Participation in this focus group is completely voluntary.

What will happen if I take part?
A member of the research team will conduct a focus group discussion with you within a small group. This discussion will take place over a maximum of 30 minutes. This will only take place where there is sufficient staffing and time, so as to not impact on the clinical care of our patients. The research team member will provide broad signposting for areas to discuss but the discussion itself is open to the group. We are keen to obtain both positive and negative aspects of the diagnostic array and its implementation. With your permission the conversation will be recorded for coding by the research team. Your identity will be anonymised, however we would like to know your clinical role in the team to assist interpretation of our research findings.

What happens at the end of the study?
The focus group discussion will be anonymised and coded by the research team. We will use the themes and some direct quotes discussed by the focus group and publish these findings alongside our scientific findings of the RASCALS project.

What if I want to withdraw from the study?
You may withdraw your consent to participate from the project at any time. Withdrawal of consent will be a confidential process and will not fed back to staff or management within the clinical service.

For peer review only - http://bmjopen.bmj.com/site/about/guidelines.xhtml

Who has reviewed the study?
All research in the NHS is looked at by independent group of people, called a Research Ethics Committee, to protect your interests. This study has been reviewed by the research advisory committee of NHS Cambridge University Hospitals NHS Foundation Trust and the research ethics committee of Bradford-Leeds.

Thank you for considering your participation in this research project.

For peer review only

For peer review only - http://bmjopen.bmj.com/site/about/guidelines.xhtml

Department of Paediatric Intensive Care

Cambridge University Hospitals NHS
NHS Foundation Trust

Dr Roddy O'Donnell
Dr Shruti Agrawal
Dr David Inwald
Dr Riaz Kayani
Dr Girish Neelegowda
Dr Nazima Pathan
Dr Stewart Reid
Dr Francesc Torres

Addenbrooke's Hospital
Hills Road
Cambridge CB2 0QQ

Switchboard: 01223 245151
www.addenbrookes.org.uk

Rapid Assay for Sick Children with Acute Lung infection Study
Staff focus group – experiences of TaqMan diagnostic array

RASCALS

Rapid Assay for Sick Children with Acute Lung infection Study

Thank you for taking the time to consider participating in the focus group for 'RASCALS' – the Rapid Assay for Sick Children with Acute Lung infection Study and reading the information sheets.

If you would like to participate in the focus group, please read and sign the following forms.

We will provide you with one copy of this form and retain a second copy securely in our research department at the hospital.

Kind regards,

The Paediatric Intensive Care Unit Research Team
RASCALS researchers: Dr Nazima Pathan, Dr John Clark, Dr Iain Kean, Dr Estée Török, Prof Gordon Dougan, Prof Stephen Baker, Dr Vilas Navapurkar, Ms Esther Daubney & Ms Deborah White.
Phone: 01223 336883
Email: np409@medschl.cam.ac.uk

For peer review only - http://bmjopen.bmj.com/site/about/guidelines.xhtml

**Participant identification number**:	**Initials**
1. I have read the information sheet on this project, dated ___/___/____ (Version________) and have been given a copy to keep. I have been able to ask questions about the project and I understand why the research is being done and any risks involved.	
2. I know how to contact the research team if I need to, and how to get information about the results of the research.	
3. I understand that the focus group interview will be recorded, and this data will be transcribed with participant identity anonymised by the research team.	
4 I understand that direct quotes from the deidentified interview transcript may be included in published research findings.	
5. I understand that any information collected as part of this study will be stored securely in line with current NHS and University guidelines. I understand that only members of the clinical project team will have access to my identifiable information; all others analysing my data will only have access to anonymised data. I understand that my data will be retained for 15 years.	
6. I understand that the information collected may be used to support other research in the future, and may be shared anonymously with other academic and commercial researchers external to the project within the UK and beyond	
7. I consent to taking part in this study	

For peer review only

For peer review only - http://bmjopen.bmj.com/site/about/guidelines.xhtml

Written consent

Carer

Name (Capitals):____

Signature:______________________________ Date: dd/mm/yyyy

Relationship to patient: ________________________________

Person obtaining consent

Name (Capitals):____

Signature:______________________________ Date: dd/mm/yyyy

Role: _____________________________

Electronic consent

Carer name (Capitals):____

Person obtaining consent

Name (Capitals):____

Signature:______________________________ Date: dd/mm/yyyy

Role: _____________________________

Verbal consent

Carer name (Capitals):____

Person obtaining consent

Name (Capitals):____

Signature:______________________________ Date: dd/mm/yyyy

Role: _____________________________

Witness to verbal consent

Name (Capitals):____

Signature:______________________________ Date: dd/mm/yyyy

Role: _____________________________

Primary investigator countersign for electronic and unwitnessed verbal consent

Dr Nazima Pathan _____________________________ Date: dd/mm/yyyy

For peer review only

For peer review only - http://bmjopen.bmj.com/site/about/guidelines.xhtml

Reporting checklist for diagnostic test accuracy study.

Based on the STARD guidelines.

Instructions to authors

Complete this checklist by entering the page numbers from your manuscript where readers will find each of the items listed below.

Your article may not currently address all the items on the checklist. Please modify your text to include the missing information. If you are certain that an item does not apply, please write "n/a" and provide a short explanation.

Upload your completed checklist as an extra file when you submit to a journal.

In your methods section, say that you used the STARDreporting guidelines, and cite them as:

Bossuyt PM, Reitsma JB, Bruns DE, Gatsonis CA, Glasziou PP, Irwig L, LijmerJG Moher D, Rennie D, de Vet HCW, Kressel HY, Rifai N, Golub RM, Altman DG, Hooft L, Korevaar DA, Cohen JF, For the STARD Group. STARD 2015: An Updated List of Essential Items for Reporting Diagnostic Accuracy Studies.

		Reporting Item	Page Number
**Title or abstract**			
None	#1	Identification as a study of diagnostic accuracy using at least one measure of accuracy (such as sensitivity, specificity, predictive values, or AUC)	
5			
**Abstract**			
None	#2	Structured summary of study design, methods, results, and conclusions (for specific guidance, see STARD for Abstracts https://www.equator-network.org/reporting-guidelines/stard-abstracts/)	

For peer review only

3

Introduction

None	#3	Scientific and clinical background, including the intended use and clinical role of the index test	4
None	#4	Study objectives and hypotheses	5

Methods

Study design	#5	Whether data collection was planned before the index test and reference standard were performed (prospective study) or after (retrospective study)	

7

Participants	#6	Eligibility criteria	6
Participants	#7	On what basis potentially eligible participants were identified (such as symptoms, results from previous tests, inclusion in registry)	

7

Participants	#8	Where and when potentially eligible participants were identified (setting, location and dates)	7
Participants	#9	Whether participants formed a consecutive, random or convenience series	7
Test methods	#10	Index and reference tests in sufficient detail to allow replication	8
Test methods	#11	Rationale for choosing the reference standard (if alternatives exist)	11
Test methods	#12	Definition of and rationale for test positivity cut-offs or result categories of the index and reference tests, distinguishing pre-specified from exploratory	

11

Test methods	#13	Whether clinical information and reference standard results were available to the performers / readers of the index test; Whether	

For peer review only

		clinical information and index test results were available to the assessors of the reference standard	
8			
Analysis	#14	Methods for estimating or comparing measures of diagnostic accuracy	14
Analysis	#15	How indeterminate index test or reference standard results were handled	N/A
Analysis	#16	How missing data on the index test and reference standard were handled	N/A
Analysis	#17	Any analyses of variability in diagnostic accuracy, distinguishing pre-specified from exploratory	N/A
Analysis	#18	Intended sample size and how it was determined	7
**Results**			
Participants	#19	Flow of participants, using a diagram	Figure 2
Participants	#20	Baseline demographic and clinical characteristics of participants	Protocol paper N/A
Participants	#21	Distribution of severity of disease in those with the target condition, and distribution of alternative diagnoses in those without the target condition	Protocol paper N/A
Participants	#22	Time interval and any clinical interventions between index test and reference standard	Protocol paper N/A
Test results	#23	Cross tabulation of the index test results (or their distribution) by the results of the reference standard	
Protocol paper N/A			
Test results	#24	Estimates of diagnostic accuracy and their precision (such as 95% confidence intervals)	Protocol paper N/A
Test results	#25	Any adverse events from performing the index test or the reference standard	Protocol paper N/A

For peer review only

Discussion

None	#26	Study limitations, including sources of potential bias, statistical uncertainty, and generalisability	3
None	#27	Implications for practice, including the intended use and clinical role of the index test	Protocol paper N/A

Other information

None	#28	Registration number and name of registry	14
None	#29	Where the full study protocol can be accessed	N/A
None	#30	Sources of funding and other support; role of funders	2

Notes:

- 20: Protocol paper N/A
- 21: Protocol paper N/A
- 22: Protocol paper N/A
- 23: Protocol paper N/A
- 24: Protocol paper N/A
- 25: Protocol paper N/A
- 27: Protocol paper N/A The STARD checklist is distributed under the terms of the Creative Commons Attribution License CC-BY. This checklist was completed on 05. August 2021 using https://www.goodreports.org/, a tool made by the EQUATOR Network in collaboration with Penelope.ai

For peer review only

BMJ Open

Rapid Assay for Sick Children with Acute Lung infection Study (RASCALS) – diagnostic cohort study protocol

Journal:	*BMJ Open*
Manuscript ID	bmjopen-2021-056197.R1
Article Type:	Protocol
Date Submitted by the Author:	20-Oct-2021
Complete List of Authors:	Clark, John; University of Cambridge, Department of Paediatrics; Cambridge University Hospitals NHS Foundation Trust<br>Kean, Iain; University of Cambridge, Department of Paediatrics<br>Curran, Martin; Clinical Microbiology and Public Health Laboratory<br>Khokhar, Fahad; University of Cambridge, Cambridge Institute of Therapeutic Immunology and Infectious Disease<br>White, Deborah; Cambridge University Hospitals NHS Foundation Trust<br>Daubney, Esther; Cambridge University Hospitals NHS Foundation Trust<br>Conway-Morris, Andrew; Cambridge University Hospitals NHS Foundation Trust<br>Navapurkar, Vilas; Cambridge University Hospitals NHS Foundation Trust<br>Bartholdson-Scott, Josefin; University of Cambridge, Cambridge Institute of Therapeutic Immunology and Infectious Disease<br>Maes, Mailis; University of Cambridge, Cambridge Institute of Therapeutic Immunology and Infectious Disease<br>Bousfield, Rachel; Cambridge University Hospitals NHS Foundation Trust<br>Gouliouris, Theodore; University of Cambridge, Department of Medicine<br>Agrawal, Shruti; University of Cambridge, Department of Paediatrics; Cambridge University Hospitals NHS Foundation Trust<br>Inwald, David; Cambridge University Hospitals NHS Foundation Trust<br>Zhang, Zhenguang; University of Cambridge, Department of Paediatrics<br>Torok, Mili; University of Cambridge School of Clinical Medicine, Department of Medicine<br>Baker, Stephen; University of Cambridge, Cambridge Institute of Therapeutic Immunology and Infectious Disease<br>Pathan, Nazima; University of Cambridge, Department of Paediatrics; Cambridge University Hospitals NHS Foundation Trust
**Primary Subject Heading**:	Paediatrics
Secondary Subject Heading:	Intensive care, Infectious diseases, Respiratory medicine
Keywords:	Molecular diagnostics < INFECTIOUS DISEASES, Respiratory infections < THORACIC MEDICINE, Paediatric intensive & critical care < ANAESTHETICS

SCHOLARONE™
Manuscripts

I, the Submitting Author has the right to grant and does grant on behalf of all authors of the Work (as defined in the below author licence), an exclusive licence and/or a non-exclusive licence for contributions from authors who are: i) UK Crown employees; ii) where BMJ has agreed a CC-BY licence shall apply, and/or iii) in accordance with the terms applicable for US Federal Government officers or employees acting as part of their official duties; on a worldwide, perpetual, irrevocable, royalty-free basis to BMJ Publishing Group Ltd ("BMJ") its licensees and where the relevant Journal is co-owned by BMJ to the co-owners of the Journal, to publish the Work in this journal and any other BMJ products and to exploit all rights, as set out in our licence.

The Submitting Author accepts and understands that any supply made under these terms is made by BMJ to the Submitting Author unless you are acting as an employee on behalf of your employer or a postgraduate student of an affiliated institution which is paying any applicable article publishing charge ("APC") for Open Access articles. Where the Submitting Author wishes to make the Work available on an Open Access basis (and intends to pay the relevant APC), the terms of reuse of such Open Access shall be governed by a Creative Commons licence – details of these licences and which Creative Commons licence will apply to this Work are set out in our licence referred to above.

Other than as permitted in any relevant BMJ Author's Self Archiving Policies, I confirm this Work has not been accepted for publication elsewhere, is not being considered for publication elsewhere and does not duplicate material already published. I confirm all authors consent to publication of this Work and authorise the granting of this licence.

For peer review only

Rapid Assay for Sick Children with Acute Lung infection Study (RASCALS) – diagnostic cohort study protocol

John A Clark MBBS [1, 2*], Iain Kean PhD [1], Martin D Curran PhD [3], Fahad Khokhar BSc [4], Deborah White MSc [2], Esther Daubney BSc [2], Andrew Conway Morris PhD [2], Vilas Navapurkar FFICM [2], Josefin Bartholdson Scott PhD [4], Mailis Maes MPhil [4], Rachel Bousfield BMBS [2, 3], Theodore Gouliouris PhD FRCPath [2, 3], Shruti Agrawal MD FRCPCH [1, 2], David Inwald PhD FRCPCH [2], Zhenguang Zhang PhD [1], M. Estée Török PhD FRCP FRCPath [2, 3], Stephen Baker PhD [4] and Nazima Pathan PhD FRCPCH [1, 2*]

1. Department of Paediatrics, University of Cambridge
2. Cambridge University Hospitals NHS Foundation Trust
3. Public Health England, Clinical Microbiology and Public Health Laboratory, Cambridge
4. Cambridge Institute of Therapeutic Immunology and Infectious Disease, University of Cambridge

*Corresponding author: Dr John Clark, Department of Paediatrics, Level 8, Addenbrooke's Hospital, Cambridge Biomedical Campus, Cambridge CB2 0QQ. Email: jac302@cam.ac.uk

For peer review only

Acknowledgements: The authors would like to thank the staff at Addenbrooke's Hospital for supporting the development and implementation of this study; Helen Starace and Colin Hamilton (physiotherapists) for assisting with developing NB-BAL sampling procedure; Adam Palmer (PICU data manager) for providing admissions data; and Claire Jenkins (scientist) for assisting with sample workflow.

Trial sponsor contact: Stephen Kelleher, Cambridge University Hospitals NHS Foundation Trust, Hills Rd, Cambridge CB2 0QQ; research@addenbrookes.nhs.uk Ph: +441223217418

Word count: 3860

Key words: Pathology, Molecular; Pneumonia; Pneumonia, Ventilator-Associated; Intensive Care Units, Pediatric; Antimicrobial stewardship

Abstract

Introduction: Lower respiratory tract infection (LRTI) is the most commonly treated infection in critically ill children. Pathogens are infrequently identified on routine respiratory culture, and this is a time-consuming process. A syndromic approach to rapid molecular testing that includes a wide range of bacterial and fungal targets has the potential to aid clinical decision making and reduce unnecessary broad spectrum antimicrobial prescribing. Here we describe a single-centre prospective cohort study investigating the use of a 52-pathogen TaqMan array card (TAC) for LRTI in the Paediatric Intensive Care Unit (PICU).

Methods and analysis: Critically ill children with suspected LRTI will be enrolled to this 100 patient single-centre prospective observational study in a PICU in the East of England. Samples will be obtained via routine non-bronchoscopic bronchoalveolar lavage (NB-BAL) which will be sent for standard microbiology culture in addition to TAC. A blood draw will be obtained via any existing vascular access device. The primary outcomes of the study will be i) concordance of TAC result with routine culture and 16S rRNA gene sequencing and ii) time of diagnostic result from TAC versus routine culture. Secondary outcomes will include impact of the test on total antimicrobial prescriptions, a description of the inflammatory profile of the lung and blood in response to pneumonia and a description of the clinical experience of medical and nursing staff using TAC.

Ethics and dissemination: This study has been approved by the Yorkshire and the Humber – Bradford Leeds Research Ethics Committee (REC reference 20/YH/0089). Informed consent will be obtained from all participants. Results will be published in peer reviewed publications and international conferences.

Trial registration number: NCT04233268; Pre-results

Strengths and limitations of this study

- This study describes clinical application of a customisable rapid diagnostic respiratory microarray in critically ill children.
- The sampling technique for this project is NB-BAL, which is a reliable sampling method for deep respiratory samples, whilst previous studies have utilised upper

For peer review only

airway samples, or endotracheal tube (ETT) secretions which may be prone to contamination.

- Following this single-centre study, a wider multi-centre evaluation will be required to determine feasibility and cost efficiency of this diagnostic method.
- There is no universally agreed diagnostic criteria for pneumonia in children, therefore clinical suspicion of pneumonia by the treating team rather than a specific set of diagnostic criteria are being used for enrolment.

Introduction

Lower respiratory tract infection (LRTI) is a leading cause of admissions to paediatric intensive care units (PICUs) in the UK, and greatest worldwide cause of mortality in young children.[1,2] PICU physicians regularly prescribe antimicrobial therapy for LRTI, as it is difficult to ascertain whether children have an underlying primary or secondary bacterial infection and withholding or delaying treatment where indicated poses significant risk.[3] Clinical prediction scores for pneumonia have low specificity in children,[4,5] and infection on standard microbiological tests is confirmed in as little as 22% of treated LRTI.[6,7]

Rapid diagnostic tests have the potential to reduce untargeted antimicrobial use.[8] Most currently available molecular respiratory diagnostic panels include common viruses and a limited number of atypical bacterial pathogens such as *Legionella pneumophila* and *Mycoplasma pneumoniae* which are difficult to grow on culture.[9,10] Some, such as the FilmArray Pneumonia Panel (BioFire Diagnostics, Utah, USA) and Curetis Unyvero pneumonia cartridge (Holzgerlingen, Germany) offer the advantage of being able to be undertaken as a point of care test with sufficient staffing and training. However they are constrained to testing manufacturer defined molecular targets.[11–13]

The TaqMan array card (TAC) (Thermo Fisher Scientific, California) is a probe-based qPCR assay undertaken in a 384 well plate format. Advantages of TAC are the ability to customise molecular targets – allowing new targets to be incorporated according to local epidemiology, and the ability to run multiple samples on a single card improving cost efficiency.[14] This may include up to eight clinical samples per card depending on the number of targets sought per sample.

For peer review only - http://bmjopen.bmj.com/site/about/guidelines.xhtml

TAC performs reliably on deep respiratory samples obtained from adults with suspected ventilator associated pneumonia (VAP).[15] In children, studies of TAC as a LRTI diagnostic are limited. On extensive search of medical literature, we identified three key studies of TAC in children with suspected pneumonia. One utilised nasopharyngeal/oropharyngeal and sputum in hospitalised children.[16] The second study obtained endotracheal tube aspirate samples in 25 children with suspected hospital acquired pneumonia with a limited TAC inclusive of seven bacteria.[17] The third study tested expectorated sputum samples with an eight bacterial pathogen TAC.[18] TAC improved diagnostic yield of samples in all studies,[16–18] but was not used for diagnostic purposes. Obtaining true deep respiratory samples is invasive and technically difficult in children, requiring advanced airway management. These studies used less invasively collected samples as a proxy for bronchoalveolar sampling of the lower respiratory tract. Given the heavy use of systemic antimicrobial therapy in the care of critically ill children, evaluation of TAC to guide antimicrobial therapy requires further evaluation. We are therefore undertaking this study to understand the performance and impact of TAC in the PICU setting.

TAC has been evaluated for a number of other paediatric applications, including bloodstream, central nervous system, and upper respiratory tract infection, [19–24] but these studies did not evaluate the utility of TAC in clinical diagnoses. These studies demonstrated high sensitivity and specificity of TAC, but it is difficult to extrapolate this into the clinical setting due to variation in molecular targets selected; regional microbiology; and key differences in clinical practice such as timing of antimicrobial administration.

TAC interpretation requires an approach that is distinct from routinely performed investigations such as culture on which only 1-2 predominant species are generally reported. The TAC array may identify several potential pathogens. Identifying multiple micro-organisms in the lungs may be helpful; however, the interpretation can be challenging for clinicians to determine antimicrobial prescriptions. In the case of hospital-acquired LRTI, this may be due to infiltration of the respiratory tract by bacteria from the dysbiotic intestinal microbiome. Community-acquired LRTI may also have several pathogens, with co-infection by bacterial and viral pathogens a recognised problem in critically ill children.[25] The precise identification of multiple organisms in the lungs in parallel should in theory help to guide the use of antimicrobial therapy, but at a clinical level it demands an understanding of how to interpret the data from molecular pathogen assays. To robustly evaluate TAC, comparison of

For peer review only - http://bmjopen.bmj.com/site/about/guidelines.xhtml

the assay to a culture-independent technique such as metagenomics can identify microorganisms that may have been eliminated due to prior antimicrobial therapy.

Whilst large-scale studies may assess individual molecular targets included on TAC for their performance, a more holistic approach of assessing an entire multi-pathogen array, including targets at genus and species level, will give an indication of its clinical application.

Hypothesis

TAC will provide greater sensitivity and a faster turnaround time than standard microbiology tests for the diagnosis of LRTI in critically unwell children.

Methods and analysis

Setting

Patients will be enrolled to the study in a 13 bedded general PICU at Addenbrooke's Hospital, Cambridge, England. The PICU manages neurosurgical and trauma cases but does not support extracorporeal membrane oxygenation or cardiac surgical patients.

Eligibility criteria

The eligibility criteria are:

1. The child is aged less than 18 years old;
2. the child is receiving mechanical ventilation at the time of enrolment; and
3. the child is commencing or already receiving antimicrobial therapy to treat suspected or confirmed LRTI.

The exclusion criteria are:

1. The patient has a non-survivable illness and is no longer on an active treatment pathway and/or
2. the child is <37 weeks corrected gestation.

These criteria ensure the patient is able to have samples obtained via NB-BAL. Enrolment of children based on antimicrobial prescription for LRTI has been selected rather than the use of clinical features for pneumonia, given there is no consistent and reliable clinical scoring

For peer review only

system for this condition.[26,27] Premature infants have been excluded from enrolment due to the sampling procedure not being tested, to our knowledge in this group of patients, and these infants having distinct pneumonia aetiology that would require separate evaluation.

Selection of participants

All children admitted to PICU at the study centre will be screened for enrolment into the study by nursing and medical staff working on the unit. A deferred consent process for up to 48 hours will allow carers to be approached sensitively by the research team, whilst ensuring samples are obtained within a reasonable timeframe to maximise yield. Consent will be obtained via written, electronic, and verbal formats via research nursing staff, and permission also obtained for COVID-19 related work (appendix A and B). Co-enrolment will be permitted in this study so long as there is no impact on the primary outcomes of either project.

Sample size measurement

This study aims to achieve a relative increase in the sensitivity of lower respiratory tract culture using TAC by 60%. This is a conservative target, given previous paediatric evaluations of TAC have had a relative increase in detection by >100% in comparison to routine investigations.[16,17] However, the present study is distinct in its sampling approach, patient population and TAC configuration, hence outcomes are difficult to estimate based on available literature.

Previous study on this unit has identified that 22% of cases of possible LRTI are confirmed on culture.[7] To increase bacterial confirmation by 60%, a total of 85 patients are required as per power calculation (figure 1).[28] The total has been rounded up to 100 to account for possible sampling related issues that may occur.

Intervention

Diagnostic TAC will be undertaken on NB-BAL sample obtained as soon as possible following the time of enrolment (Figure 2). The same sample will be used for standard microbiology culture in all patients; however, the clinical team will determine whether viral qPCR panel, extended fungal or mycobacterial cultures or investigations are also indicated. Samples will only be processed during business hours Monday-Friday due to availability of research and laboratory staff.

For peer review only - http://bmjopen.bmj.com/site/about/guidelines.xhtml

Samples will be obtained as part of standard practice by physiotherapy, nursing and medical staff that routinely perform the sampling procedure. The results of the test will be reported alongside routine tests, and impact of these results will be investigated. Positive results will be reported with their corresponding Cycle threshold (Ct) values, to assist interpretation where a detection may either be a true pathogen, or pathobiont. Standard practice in this PICU is for all patients to be reviewed in twice weekly microbiology rounds. In these meetings the patient's clinical presentation, progress, biochemical and microbiology results, and treatment is reviewed. The clinical team have a low threshold in seeking input from the microbiology team, which provide a 24 hour on-call service. This multi-disciplinary approach will assist the clinical team in situations where there are detections on TAC of unclear significance.

Study objectives

Primary

1. Determine the sensitivity and specificity of TAC in the detection of lower respiratory bacterial pathogens.
2. Compare time to diagnosis of TAC versus standard microbiology culture in the diagnosis of LRTI.

Secondary

1. Describe the microorganisms detected on TAC that were not detected using standard diagnostic tests.
2. Describe the lung microbiome of critically ill children with LRTI using 16S rRNA gene sequencing.
3. Understand the impact of TAC on total antimicrobial prescriptions in PICU by total prescriptions and by antimicrobial class.
4. Describe the impact of TAC on antimicrobial decision making according to PICU consultants.
5. Describe the host inflammatory response to LRTI in the lung and blood according to pathogen present.
6. Describe the experience of a tertiary PICU in the implementation of TAC in the clinical setting.

For peer review only - http://bmjopen.bmj.com/site/about/guidelines.xhtml

Outcome measures

Primary

1. TAC result (cycle threshold value).
2. Respiratory culture result (colony forming units/ml).
3. Respiratory viral panel result (cycle threshold value).
4. Time to result (hours).

Secondary

1. TAC result (cycle threshold [Ct] value).
2. Blood culture result.
3. Total days of antimicrobial prescriptions in PICU – % total days of PICU admission and days free of antimicrobial therapy in PICU at 28 days.
4. Reported n (%) of change in antimicrobial prescription type and duration, and n (%) of children able to move out of side rooms due to exclusion of infectious pathogens.
5. Days free of mechanical ventilation at 28 days following PICU admission.
6. Days free of PICU at 28 days following PICU admission.
7. 16S rRNA gene sequencing result – n (%) of total reads, Shannon's diversity index
8. Cytokine concentration (pg/ml) in NB-BAL samples and blood.
9. Semi-structured interview thematic analysis - description of implementation and interpretation considerations of TAC in the PICU.

Sampling and Laboratory Procedures

NB-BAL sampling

Deep respiratory samples will be obtained at the time of enrolment via non-bronchoscopic bronchoalveolar lavage, with some modifications made to the original procedure given SARS-CoV-2 (appendix C and D). Saline lavage volume instilled will be 1ml/kg of patient weight to a maximum of 10mL. Saline is delivered via the in-line suction catheter system and the sample collected in a universal container via a sputum trap. This approach minimises risk of aerosolisation of pathogens such as SARS-CoV-2. Samples will be immediately delivered to the microbiology containment level 3 laboratory and stored at 4°C until staff are available to process the samples, between 8AM-5PM weekdays. Samples will be split into aliquots for microbiology culture, nucleic acid extraction, and any additional clinical tests required.

Nucleic acid extraction

For peer review only - http://bmjopen.bmj.com/site/about/guidelines.xhtml

Up to 750 µl of sample will be added to a 2mL microtube containing a mixture of ceramic beads with 750 µl of L6 buffer (Qiagen). A minimum of 100uL will be required for extraction and brought up to 750 µl with nuclease free water if low volume. The sample will then be vortexed and incubated for 10 minutes. Samples will then be processed using an EZ1 virus mini kit (v2.0) using an EZ1 advanced XL (Qiagen) in up to 14 samples per run including an L6 buffer-RNase free water negative control.[29]

TaqMan array card

A custom screening panel was developed and validated via an adult intensive care study that took place in this institution.[30] Retrospective review of organisms identified on routine tests for severe LRTI in this PICU demonstrated good coverage by this panel.[7] The targets of the panel are as shown in table 1. Many pathogens are assigned two targets to reduce false positive test interpretations. A target for the MecA gene has been included as it is commonly present in methicillin-resistant *Staphylococcus aureus*. The TAC includes endogenous control RNase P, internal control MS2 and 18S rRNA gene. TAC configuration was altered on 05/02/2021, to include SARS-CoV-2 targets, and opportunistically incorporate *Leptospira* as this organism was of relevance to the adult ICU service also using the cards. Using the same card for the adult and paediatric service allows samples to be processed in batches of up to four, reducing waste of empty lanes in the card, and minimises laboratory handling time.

For peer review only

Table 1: Molecular targets of the TaqMan diagnostic card for lower respiratory tract infection

Type	Target	Number of targets
Bacterial	16S rRNA gene*	2
	Acinetobacter baumannii	3
	Bacteroides fragilis	1
	Bordetella pertussis	2
	Chlamydia pneumoniae	1
	Chlamydia psittaci	1
	Coxiella burnetti	1
	Elizabethkingia meningoseptica	2
	Enterobacter cloacae	2
	Enterobacteriaceae	1
	Enterobacteriaceae Proteus	1
	*Enterococcus faecalis**	1
	*Enterococcus faecium**	2
	Escherichia coli	2
	Haemophilus influenzae	2
	Klebsiella pneumoniae	2
	Legionella pneumophilia	1
	Legionella spp	1
	Moraxella catarrhalis	1
	Morganella morganii	1
	Mycobacterium spp	1
	Mycoplasma pneumoniae	2
	Mycobacterium tuberculosis	2
	Neisseria meningitidis	1
	Pseudomonas aeruginosa	2
	Serratia marcescens	2
	Staphylococcus aureus	2
	Staphylococcus – coagulase negative	1
	Staphylococcus epidermidis	1
	Staphylococcus - PVL toxin	1
	Stenotrophomonas maltophilia	1
	Streptococcus pneumoniae	2
	Streptococcus pyogenes	2
	Streptococcus spp	2
Viral	Adenovirus	2
	Bocavirus	1

For peer review only - http://bmjopen.bmj.com/site/about/guidelines.xhtml

	Cytomegalovirus	1
	Epstein Barr Virus	1
	Enterovirus	2
	Herpes simplex virus	2
	Human coronavirus NL63	1
	Human coronavirus OC43/HKU1	1
	Human coronavirus OC43	1
	Human coronavirus 229E	1
	Human metapneumovirus	1
	Human parainfluenza virus 1	2
	Human parainfluenza virus 2	1
	Human parainfluenza virus 3	2
	Human parainfluenza virus 4	1
	Influenza A	2
	Influenza A – H1 (2009)	1
	Influenza A – H3	1
	Influenza B	2
	Parechovirus	1
	Respiratory syncytial virus (any)	1
	Respiratory syncytial virus A	1
	Respiratory syncytial virus B	1
	Rhinovirus	2
Fungal	*Aspergillus* 28S rRNA gene	2
	Aspergillus fumigatus	1
	Candida albicans	1
	Candida species	1
	Fungal 18S rRNA gene	1
	Pneumocystis jirovecii	1
AMR gene	*mecA*	1
Controls	18S rRNA gene	1
	MS2 internal control	2
	RNase P internal control	1
**Total wells**		**96**

*In the four enrolments occurring from 5 February 2021 onwards, these targets were replaced to include 3 targets for SARS-CoV-2, 1 target for *Leptospira* and an additional target for legionella species.

For peer review only - http://bmjopen.bmj.com/site/about/guidelines.xhtml

For each sample, 50 µl of total nucleic acid is added to 50 µl of TaqMan Fast Virus 1-step mastermix (Thermo Fisher) and 100 µl of RNase free water. A total of 98 µl is then added to 2 lanes of the array. Each lane comprises 48 molecular targets, with the array configured to 96 targets of interest. Therefore, 4 patient samples are loaded into 2 lanes each for a total of 8 lanes per plate.

The RT-PCR will be undertaken on a QuantStudio 7 Flex (Thermo Fisher) according to the following validated protocol: 50°C for 5 minutes, 95°C for 20 seconds, 45 cycles of 95°C for 1 second, 60°C for 20 seconds.[15] qPCR Ct values with clear amplification curves will be reported and documented on the electronic medical record.

Conventional pathogen testing

Samples will be processed according to Public Health England laboratory standards. For patients that are not immunocompromised, standard media will be used. The sample will be inoculated on chocolate agar and incubated at 35-37°C supplemented with 5-10% CO_2.[31] If the patient is immunocompromised, supplementary media will be used, and MacConkey agar and Mannitol salt/Chromogenic agar will be used with the sample incubated in air.[31] Significant growth constitutes $>10^4$ cfu/mL on NB-BAL samples, or $>10^5$ cfu/mL on endotracheal tube (ETT) aspirate. Bacterial organisms will be identified to species or genus level using MALDI-TOF mass spectrometry (Bruker Daltonics, Coventry, UK). Antimicrobial susceptibility testing will be performed using disc diffusion following EUCAST guidelines.[32]

An in-house multiplex RT-PCR assay will be used for testing NPA and NB-BAL samples for common respiratory viruses: adenovirus, enterovirus, human metapneumovirus, influenza A and B viruses, parainfluenza virus, rhinovirus, and respiratory syncytial virus. This assay is only requested when determined to be of relevance by the clinical team.

If clinically suspected, presence of *Aspergillus* spp will be tested by culture on Sabouraud dextrose agar with chloramphenicol. Serum will also be tested for the presence of galactomannan antigen. If clinically suspected PCR assay will be used to detect presence of *Pneumocystis jirovecii*.

For peer review only - http://bmjopen.bmj.com/site/about/guidelines.xhtml

16S rRNA gene sequencing

Nucleic acid samples will be quantified using a Qubit 4 fluorometer and Qubit dsDNA HS assay kit (Q32854) (ThermoFisher Scientific, Waltham, MA, USA). DNA fragment size and quality will be assessed using an Agilent TapeStation 4200 (Agilent, Santa Clara, CA, USA).

After quantification, a sequencing library will be prepared using a 16S rRNA gene Barcoding Kit (SQK – 16S024), then loaded onto a FLO-MIN106D R9.4.1 flow cell. Sequencing will be undertaken using a MinION nanopore sequencing device (Oxford Nanopore Technologies, UK). Base calling will be undertaken using the EPI2ME platform and data will be stored as FASTQ files. De-multiplexing will be completed with guppy_barcoder v5.0.11, adapters will be trimmed with porechop 0.2.4, and reads filtered with NanoFilt v2.8.0. Filtered reads will be classified using Kraken2.[33] Results will be for research purposes only and not distributed to the clinical team.

Blood sampling

Blood will be obtained for research if the child is undergoing venepuncture for clinical purposes or if there is an existing vascular access device. The maximum volume obtained will be 1ml/kg to a maximum 10ml in keeping with World Health Organisation Guidelines.[34] Collected blood will be stored in a 4°C refrigerator on the ward until the research team are able to process samples. EDTA tubes will be spun at 1300g for 10 minutes at 4°C, and aliquots stored at -20°C until batch processing.

Cytokine assay

Cytokines will be quantified from NB-BAL and plasma samples using a Bio-Plex Pro Human Cytokine Screening 48-plex kit (Bio-Rad) using standard methods.[35] This assay will be undertaken on aliquots of NB-BAL stored at -80°C until batch processing. NB-BAL will be un-diluted whilst plasma will be diluted with standard diluent HB as per manufacturer recommendations dependent on limits of detection according to the generated standard curves.

Survey and Semi-structured interviews

Senior medical staff on the PICU will be surveyed regarding their antimicrobial decision making after TAC result becomes available (Appendix E). Survey invitation will be sent via

For peer review only - http://bmjopen.bmj.com/site/about/guidelines.xhtml

RedCAP to the hospital email address of the duty PICU consultant when the TAC result becomes available.

Semi-structured interviews will be undertaken with PICU nursing and medical staff at the end of the study. They will be advertised using posters and via the internal newsletter to staff. Voluntary consent will be obtained (Appendix F and G). Sessions will be facilitated by a member of the research team and recorded. Themes and descriptions will be analysed using NVIVO 11.[36] These interviews will capture the experience of staff in implementing TAC into clinical practice, the benefits and downsides to the test, and interpretation of the test.

Patient and public involvement

Carers of children admitted to PICU identified rapid molecular diagnostic tests for early rationalisation of antimicrobial therapy to be a high priority area for research. They ranked this 4th of 73 potential national priority areas for PICU research in a 2019 multi-centre survey.[37] As this study is investigating acute clinical decision making of critical care clinicians, patients were not involved in the design of scientific study methodology. The research team will be engaging interested families who have had children admitted to this PICU for their input in the dissemination of research findings and for additional feedback on the design of patient information and consent materials.

Study status

Enrolment commenced on 10/04/2020 and enrolment is expected to be completed prior to March 2023.

Data management

Database

Data will be obtained from the electronic medical record and recorded on REDCap, a secure data management system which will be hosted by the University of Cambridge.[38] Data collected will include demographics, physiological parameters, paediatric index of mortality 3 score,[39] treatment received and investigation results from the time of presentation to hospital for the acute illness to discharge.

Statistical analysis plan

For peer review only - http://bmjopen.bmj.com/site/about/guidelines.xhtml

Data will be analysed in R.[40] Demographic data will be reported using simple descriptive statistics including mean and standard deviation. For comparisons of demographic data between groups, skewed data using non-parametric tests including Mann-Whitney U test and Kruskal-Wallis test. Normally distributed data will be compared with Student t-tests. Group correlations of quantitative data will be tested with Spearman correlation test.

All TAC detections from NB-BAL samples will be reported using the mean and range of Ct values for each molecular target. This will be compared to growth on culture in cfu/ml or Ct value for routine qPCR testing. A positive TAC result will be considered a Ct of 32 or below, which was found to correlate with microbiology culture thresholds in a previous investigation.[30]

Time to reportable results will be recorded as the time taken from collection time of the sample, which will be entered by nursing staff into the electronic medical record. The comparison of time to result of TAC versus culture will be assessed with Wilcoxon signed ranks test.

Cytokine results will be analysed using a support-vector machine approach to explore profiles of different infections.

Ethics and dissemination

Ethics and registration

This study is registered with clinicaltrials.gov (NCT04233268 – first posted 18/01/2020) and registered via the Integrated Research Application System (IRAS 277039). The study is jointly sponsored by Cambridge University Hospitals NHS Foundation Trust and the University of Cambridge. The study was approved by the Yorkshire and the Humber – Bradford Leeds Research Ethics Committee on 26/03/2020 (REC reference 20/YH/0089). The amended, current version of the protocol (8.0) was approved by the REC on 02/07/2021. Amendments to this study have primarily been a result of the SARS-CoV-2 pandemic. Additional work packages investigating SARS-CoV-2 infection in children under the overarching RASCALS project are subject to separate protocol and analysis that are not outlined in this paper.

For peer review only - http://bmjopen.bmj.com/site/about/guidelines.xhtml

For peer review only

Protocol amendments will be communicated to participants when changes are made that result in a change in procedures for the individual. Participants will be pseudonymised via creation of a sequential study ID.

Informed consent procedures

Consent will be obtained in written, electronic, or verbal format as approved by the ethics committee. Whilst written consent is preferred, alternative consent procedures were introduced due to the COVID-19 pandemic. Deferred consent for up to 48 hours will be permitted to allow time critical samples to be obtained, whilst also ensuring families are approached sensitively in the PICU.

Dissemination

Findings from this project will be reported in peer-reviewed journals and international conferences.

Appendices

Appendix A: Patient information sheet

Appendix B: Patient consent form

Appendix C: NB-BAL sampling procedure

Appendix D: NB-BAL sampling procedure modifications for COVID-19

Appendix E: Consultant survey

Appendix F: Staff focus group information sheet

Appendix G: Staff focus group consent form

Contributorship statement:

JC, MDC, ACM, VN, MET, SB, and NP conceived the study design; JC, MDC, ACM, VN, MET, SB and NP designed the study protocol; JC, IK, MDC, FK, JB-S, MM, and SB developed laboratory methods for the study. JC, DW, ED, SA, DI developed sampling procedure protocol; JC, IK, FK, ACM, RB, TG, ZZ, SB and NP developed the statistical analysis plan; JC, IK, MDC, FK, DW, ED, ACM, VN, RB, TG, SA, ZZ, SB, NP drafted this manuscript.

For peer review only - http://bmjopen.bmj.com/site/about/guidelines.xhtml

For peer review only

Competing interests: MDC is the inventor on a patent held by the Secretary of State for Health (UK government) EP2788503, which covers some of the genetic sequences used in this study. VN is a founder, director, and shareholder in Cambridge Infection Diagnostics Ltd (CID Ltd) which is a commercial company aimed at developing molecular diagnostics in infection and antimicrobial and AMR stewardship. ACM, SB, and GD are members of the Scientific Advisory Board of Cambridge Infection Diagnostics Ltd (CID Ltd). TG has received a research grant from Shionogi. All other authors declare no conflict of interest.

Funding: This project is funded by the Addenbrooke's Charitable Trust, Cambridge University Hospitals [900240] (JC, NP, MET, SB) which will provide funding for consumables, 16S rRNA gene sequencing of samples and inflammatory profiling; in addition to the NIHR Cambridge Biomedical Research Centre. The authors also receive support from the Gates Cambridge Trust [OPP1144] (JC); the Academy of Medical Sciences (MET); Wellcome Trust [215515] (SB); Wellcome Trust Clinical Research Career Development Fellowship [WT 2055214/Z/16/Z] (ACM) MRC Clinician Scientist Fellowship [MR/V006118/1] (ACM); and Action Medical Research (NP, SB, MET) [GN2751, GN2903].

This work was supported, in whole or in part, by the Bill & Melinda Gates Foundation [OPP1144]. Under the grant conditions of the Foundation, a Creative Commons Attribution 4.0 Generic License has already been assigned to the Author Accepted Manuscript version that might arise from this submission.

Data sharing plan: The research team will have access to the final study database. On completion of the project this data will be made available via a data sharing platform (Open Science Framework) and 16S rRNA gene sequencing data will be shared via the European Nucleotide Archive.

References

1. Global Burden of Disease Collaborators. Global, regional, and national age-sex specific mortality for 264 causes of death, 1980-2016: a systematic analysis for the Global Burden of Disease Study 2016. *Lancet (London, England)* **390**, 1151–1210 (2017).
2. Paediatric Intensive Care Audit Network. *Paediatric Intensive Care Audit Network*

For peer review only

Annual Report 2019. (2019).

3. Kumar, A. *et al.* Duration of Hypotension before Initiation of Effective Antimicrobial Therapy Is the Critical Determinant of Survival in Human Septic Shock. *Crit. Care Med.* **34**, 1589–1596 (2006).
4. da Silva, P. S. L., de Aguiar, V. E., de Carvalho, W. B. & Machado Fonseca, M. C. Value of clinical pulmonary infection score in critically ill children as a surrogate for diagnosis of ventilator-associated pneumonia. *J. Crit. Care* **29**, 545–550 (2014).
5. Sachdev, A. *et al.* Clinical Pulmonary Infection Score to diagnose ventilator-associated pneumonia in children. *Indian Pediatr.* **48**, 949–954 (2011).
6. Stocker, M. *et al.* Antibiotic surveillance on a paediatric intensive care unit: easy attainable strategy at low costs and resources. *BMC Pediatr.* **12**, 196 (2012).
7. Clark, J. *et al.* Low diagnostic yield and time to diagnostic confirmation results in prolonged use of antimicrobials in critically ill children. *Wellcome Open Res.* **6**, 119 (2021).
8. Byington, C. L. *et al.* The Effect of Rapid Respiratory Viral Diagnostic Testing on Antibiotic Use in a Children's Hospital. *JAMA Pediatr.* **156**, 1230–1234 (2002).
9. Whiley, H. & Taylor, M. Legionella detection by culture and qPCR: Comparing apples and oranges. *Crit. Rev. Microbiol.* **42**, 65–74 (2016).
10. Daxboeck, F., Krause, R. & Wenisch, C. Laboratory diagnosis of Mycoplasma pneumoniae infection. *Clin. Microbiol. Infect.* **9**, 263–273 (2003).
11. Papan, C. *et al.* Assessment of the multiplex PCR-based assay Unyvero pneumonia application for detection of bacterial pathogens and antibiotic resistance genes in children and neonates. *Infection* **46**, 189–196 (2018).
12. Li, J. *et al.* Rapid detection of respiratory organisms with the FilmArray respiratory panel in a large children's hospital in China. *BMC Infect. Dis.* **18**, 510 (2018).
13. Leber, A. L. *et al.* Multicenter Evaluation of BioFire FilmArray Respiratory Panel 2 for Detection of Viruses and Bacteria in Nasopharyngeal Swab Samples. *J. Clin. Microbiol.* **56**, e01945-17 (2018).
14. Maja, K. *et al.* Application of TaqMan Low-Density Arrays for Simultaneous Detection of Multiple Respiratory Pathogens . *J. Clin. Microbiol.* **49**, 2175–2182 (2011).
15. Jones, N. K. *et al.* Evaluating the use of a 22-pathogen TaqMan array card for rapid diagnosis of respiratory pathogens in intensive care. *J. Med. Microbiol.* **69**, 971–978 (2020).

For peer review only

16. Wolff, B. J. *et al.* Improved Detection of Respiratory Pathogens by Use of High-Quality Sputum with TaqMan Array Card Technology. *J. Clin. Microbiol.* **55**, 110 LP – 121 (2017).
17. Mansour, M. G. E. & Bendary, S. Hospital-acquired pneumonia in critically ill children: Incidence, risk factors, outcome and diagnosis with insight on the novel diagnostic technique of multiplex polymerase chain reaction. *Egypt. J. Med. Hum. Genet.* **13**, 99–105 (2012).
18. Hu, L., Han, B., Tong, Q., xiao, H. & Cao, D. Detection of Eight Respiratory Bacterial Pathogens Based on Multiplex Real-Time PCR with Fluorescence Melting Curve Analysis. *Can. J. Infect. Dis. Med. Microbiol.* **2020**, 2697230 (2020).
19. Diaz, M. H. *et al.* Development and Implementation of Multiplex TaqMan Array Cards for Specimen Testing at Child Health and Mortality Prevention Surveillance Site Laboratories. *Clin. Infect. Dis.* **69**, S311–S321 (2019).
20. Zhao, C. *et al.* Development of a TaqMan Array card to target 21 purulent meningitis-related pathogens. *BMC Infect. Dis.* **19**, 289 (2019).
21. Onyango, C. *et al.* Evaluation of TaqMan Array Card (TAC) for the Detection of Central Nervous System Infections in Kenya. *J. Clin. Microbiol.* **55**, (2017).
22. Zhang, C. *et al.* Detection of pathogenic microorganisms from bloodstream infection specimens using TaqMan array card technology. *Sci. Rep.* **8**, 12828 (2018).
23. Liu, K. *et al.* Evaluation of TaqMan Array card (TAC) for the detection of 28 respiratory pathogens. *BMC Infect. Dis.* **20**, 820 (2020).
24. Caserta, M. T., Yang, H., Gill, S. R., Holden-Wiltse, J. & Pryhuber, G. Viral Respiratory Infections in Preterm Infants during and after Hospitalization. *J. Pediatr.* **182**, 53-58.e3 (2017).
25. Diaz, M. H. *et al.* Identification of Bacterial and Viral Codetections With Mycoplasma pneumoniae Using the TaqMan Array Card in Patients Hospitalized With Community-Acquired Pneumonia. *Open Forum Infect. Dis.* **3**, (2016).
26. Goodman, D. *et al.* Challenges in the diagnosis of paediatric pneumonia in intervention field trials: recommendations from a pneumonia field trial working group. *Lancet Respir. Med.* **7**, 1068–1083 (2019).
27. Foglia, E., Meier, M. D. & Elward, A. Ventilator-associated pneumonia in neonatal and pediatric intensive care unit patients. *Clin. Microbiol. Rev.* **20**, 409–425 (2007).
28. Kane, S. Sample size calculator. *ClinCalc* (2021).
29. Qiagen. *EZ1 Virus Handbook.* (2010).

For Peer review only

30. Navapurkar, V. *et al.* Development and implementation of a customised rapid syndromic diagnostic test for severe pneumonia. *medRxiv* 2020.06.02.20118489 (2020). doi:10.1101/2020.06.02.20118489
31. Public Health England. *UK Standards for Microbiology Investigations: Investigation of bronchoalveolar lavage, sputum and associated specimens*. (2019).
32. EUCAST. *EUCAST disk diffusion method*. (2021).
33. Wood, D. E., Lu, J. & Langmead, B. Improved metagenomic analysis with Kraken 2. *Genome Biol.* **20**, 257 (2019).
34. Howie, S. R. C. Blood sample volumes in child health research: review of safe limits. *Bull. World Health Organ.* **89**, 46–53 (2011).
35. Bio-Rad. *Bio-Plex Pro Human Cytokine Assays - Instruction Manual*. (2021).
36. QRS International Pty Ltd. Nvivo qualitvative data analysis software. (2018).
37. Tume, L. *et al.* Research Priorities for U.K. Pediatric Critical Care in 2019: Healthcare Professionals' and Parents' Perspectives. *Pediatr Crit Care Med* **22**, e294–e301 (2021).
38. Harris, P. A. *et al.* Research electronic data capture (REDCap)—A metadata-driven methodology and workflow process for providing translational research informatics support. *J. Biomed. Inform.* **42**, 377–381 (2009).
39. Straney, L. *et al.* Paediatric index of mortality 3: an updated model for predicting mortality in pediatric intensive care*. *Pediatr. Crit. Care Med.* **14**, 673–681 (2013).
40. RStudio Team. RStudio: Integrated Development for R. (2019).

For peer review only

Figure 1: Power calculation

Calculation generated with ClinCalc[28]

Figure 2: Workflow

LRTI: Lower respiratory tract infection; qPCR: quantitative polymerase chain reaction; TAC: Taqman Array Card

For peer review only - http://bmjopen.bmj.com/site/about/guidelines.xhtml

$$N = \frac{p_0 q_0 \left\{ z_{1-\alpha/2} + z_{1-\beta} \sqrt{\frac{p_1 q_1}{p_0 q_0}} \right\}^2}{(p_1 - p_0)^2}$$

$$q_0 = 1 - p_0$$

$$q_1 = 1 - p_1$$

$$N = \frac{0.22 * 0.78 \left\{ 1.96 + 0.84 \sqrt{\frac{0.352*0.648}{0.22*0.78}} \right\}^2}{(0.352 - 0.22)^2}$$

$$N = 85$$

p_0 = proportion (incidence) of population
p_1 = proportion (incidence) of study group
N = sample size for study group
α = probability of type I error (usually 0.05)
β = probability of type II error (usually 0.2)
z = critical Z value for a given α or β

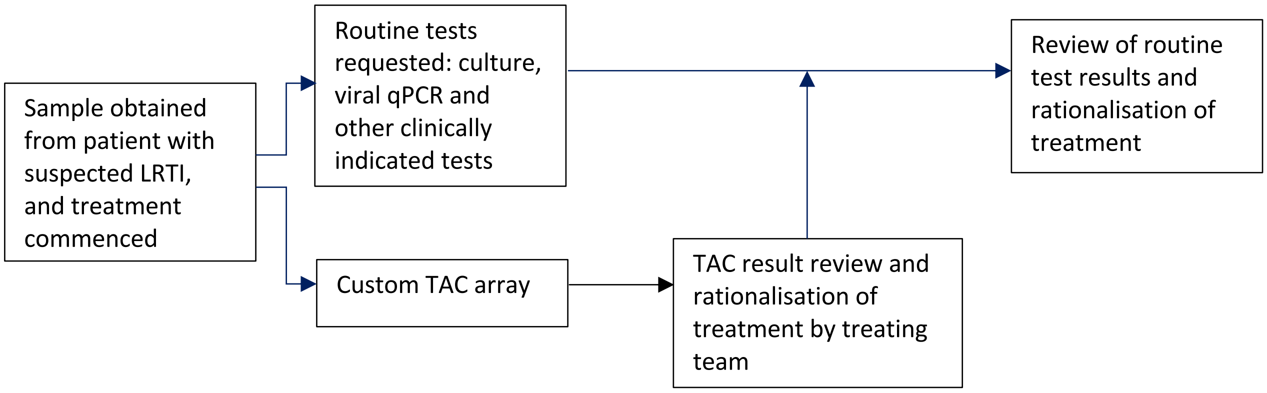

For peer review only

Department of Paediatric Intensive Care

Dr Roddy O'Donnell
Dr Rosalie Campbell
Dr David Inwald
Dr Shruti Agrawal
Dr Nazima Pathan
Dr Girish Neelegowda
Dr Riaz Kayani

Cambridge University Hospitals NHS
NHS Foundation Trust

Addenbrooke's Hospital
Hills Road
Cambridge CB2 0QQ

Switchboard: 01223 245151
www.addenbrookes.org.uk

Rapid Assay for Sick Children with Acute Lung infection Study
Invitation and Information Sheet – Parent/Carer of critically ill child

RASCALS

Rapid Assay for Sick Children with Acute Lung infection Study

Thank you for taking the time to consider having your child participate in 'RASCALS' – the Rapid Assay for Sick Children with Acute Lung infection Study.

Before you decide whether to have your child participate it is important for you to understand why the research is being done and what it will involve. Please take time to read the following information carefully and discuss it with others if you wish. Do ask us if there is anything that is not clear or if you would like more information. Take time to decide whether or not you wish to take part.

Key points about the study

- We are investigating bacteria and viruses that cause chest infection in children. In this study we will be using your child's breathing tube secretions, swabs, blood and faeces to help us improve the way we diagnose these infections.
- All the children in the study will be tested for Coronavirus 2019 (COVID-19), we will let you know the results.
- One of the rapid bacteria and virus tests is something we're trying for the first time. We will be doing this test using what is left over from your child's breathing tube sample which is needed even if they are not in the study. We will share the results of this test with you, and your child's doctors. It is possible these test results could help your child's doctors make decisions regarding your child's antibiotic treatment. We will ask them via a survey if the test was helpful.
- If your child is strongly suspected to have COVID-19 we will ask for your help collecting weekly throat swabs and either a swab of the bottom or faecal sample. This will continue for 4 weeks and will conclude with a survey about your child's symptoms.
- If at any point you no longer wish for your child to take part let your nurses and doctors know. Your child will receive high quality care during their stay in the paediatric intensive care unit whether or not they take part.

Key points about your child's information

For peer review only - http://bmjopen.bmj.com/site/about/guidelines.xhtml

- In this research study we will use information from your child's medical records. We will only use information that we need for the research study. We will let very few people know your name or contact details, and only if they really need it for this study.
- Everyone involved in this study will keep your data safe and secure. We will also follow all privacy rules. At the end of the study we will save some of the data in case we need to check it and for future research.
- We will make sure no-one can work out who you are from the reports we write. The following information pack tells you more about this.

Kind regards,
The Paediatric Intensive Care Unit Research Team

RASCALS researchers: Dr Nazima Pathan, Dr John Clark, Dr Iain Kean, Dr Estée Török, Prof Gordon Dougan, Prof Stephen Baker, Dr Vilas Navapurkar, Ms Esther Daubney & Ms Deborah White.
Phone: 01223 336883
Email: np409@medschl.cam.ac.uk

*This study has been co-sponsored by Cambridge University Hospitals NHS Foundation Trust and the University of Cambridge. Any reference to 'we' or 'us' in these documents refers to these entities.

For peer review only - http://bmjopen.bmj.com/site/about/guidelines.xhtml

Carer Information: Rapid Assay for Sick Children with Acute Lung infection Study

What is the purpose of this study?

The Department of Paediatric Intensive Care at Addenbrooke's Hospital is actively involved in research in children who are critically ill. We want to do research that helps us develop better ways of diagnosing and treating children admitted to our unit. Our research nurses and doctors would like to talk to you about the work that we are doing.

In this specific study, we are interested in COVID-19 and in the diagnosis and treatment of lung infections. Currently, the best tests we have available for diagnosing lung infections are slow, and often can't detect the bugs that have caused infection. We are trying a new rapid infection test to see if it might be faster and able to detect these bugs more often. We will share the results of the test with you and your child's doctors. We will ask your doctors whether the test had any impact on their decisions on the type and duration of antibiotics they prescribe. Your child will still have all their routine tests done as well.

In addition to the rapid infection test, we will be using the samples we collect to test for new markers of infection and COVID-19.

Why has my child been chosen?

We are studying children that are unwell in the Paediatric Intensive Care Unit that have potential COVID-19 and/or lung infection requiring intubation and ventilation (a breathing tube down the windpipe and the support of a breathing machine). We we are aiming to improve tests and obtain more information about COVID-19.

Who is organising the study?

The study is being organised by the Department of Paediatric Intensive Care by Dr Nazima Pathan and Dr John Clark, here at Addenbrooke's Hospital. This work is supervised by the University of Cambridge. No specific payments will be made to any of the staff involved for including your child in this study other than their normal salary.

Does my child have to take part?

It is up to you whether or not your child should take part. If you chose for your child to participate you will be given this information sheet to keep and be asked to sign a consent form. If you decide to have your child take part, you are still free to withdraw this decision at any time and without giving a reason. A decision to withdraw or a decision not to take part will not affect the standard of care your child will receive.

What will happen to my child if they take part?

Your child will receive infection tests whilst we can care for them in intensive care, and the 4 weeks that follow if they are strongly suspected to have COVID-19. The first set of tests will be done by hospital staff. If your child is well enough to go home in the following 4 weeks we will ask for your help taking throat swabs and faecal samples or swabs of the bottom. We will provide directions on how to do this and how to post the samples back to us. A chart of the tests that will be done is on the following page.

For peer review only - http://bmjopen.bmj.com/site/about/guidelines.xhtml

When	What test?		What for?	Will I find out the result?
As soon as possible	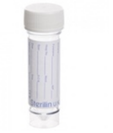	Breathing tube sample	Bacteria and viruses	Yes
			Coronavirus 2019	Yes
			Markers of infection and antibiotic resistance	No – this will be for research only
	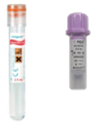	Blood	Markers of infection	No – this will be for research only
			Coronavirus 2019	Yes
	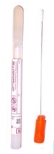	Nasopharyngeal swab (swab at the back of the nose) or nasopharyngeal aspirate (secretions from back of the nose)	Coronavirus 2019	Yes
	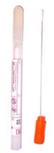	Throat swab	Coronavirus 2019	Yes
	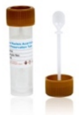	Faecal sample or rectal swab	Coronavirus 2019	Yes*
			Markers of infection and antibiotic resistance	No – this will be for research only
If there is strong clinical suspicion or tests confirm Coronavirus 2019, the following samples will be obtained.				
2-4 days later	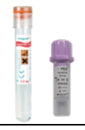	Blood	Markers of infection and antibiotic resistance	No – this will be for research only
			Coronavirus 2019	Yes*
5-7 days later	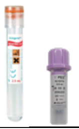	Blood	Markers of infection and antibiotic resistance	No – this will be for research only
			Coronavirus 2019	Yes*
Weekly for 4 weeks	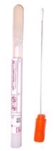	Throat swab	Coronavirus 2019	Yes*
	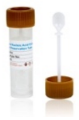	Faecal sample or rectal swab	Coronavirus 2019	Yes*
			Markers of infection and antibiotic resistance	No – this will be for research only

* The first set of nose and throat swabs we do will be processed quickly, however COVID-19 tests on other samples may take multiple weeks given resources available in the health service.

None of the samples we obtain will result in extra procedures for your child. The blood sample amount will be within safe limits according to the weight of your child, which we will take from their central line. We will not need to use any extra needles to take this blood.

For peer review only - http://bmjopen.bmj.com/site/about/guidelines.xhtml

For peer review only

The rapid bacteria and virus test we are trying has not been used before in sick children, therefore it will be in addition to our normal infection tests. We will let you and your child's doctors know the results of this new test. We will ask the doctors whether this test made any difference to the antibiotic treatment they prescribe. This will be via a short questionnaire we have developed.

All the samples we take will be frozen once we have finished our study and stored for up to 10 years. This means that with ethics committee approval they could be used for other related studies.

Why have you saved samples prior to asking me?
To best detect infection tests need to be done as early as possible. The tests don't work as well once more and more doses of antibiotics are given. We put some of the samples we need to take aside so this study would not mean we were doing extra procedures on your child. Whether we use these samples for our study is your decision.

What do I and my child have to do?
No specific restrictions need to be placed upon your child as a consequence of participating in the study. Your child would continue with any regular medication.

Aside from taking and posting the samples mentioned earlier, we will ask you to complete a short online survey. We will send you a link to this survey via your email address. It will contain questions about your child's symptoms and how long they lasted, in addition to whether your child was given any antibiotics once they left the intensive care unit. This data will go directly to a secure online database only accessible by the research team.

What are the possible risks / side effects of taking part?
Taking extra blood and secretion samples will not mean any significant extra risk or harm to your child. Taking the secretion samples from the breathing tube does not cause additional harm to your child's lungs.

What are the possible benefits of taking part?
The study is aimed at improving our ability to diagnose the cause of lung infections and identifying the best treatment early.

Being part of this study means additional information will be obtained regarding possible causes of infection in your child. It is not known whether the results will have any benefits / implications for your child directly. However, by taking part you may contribute to helping children and adults suffering from chest infections in the future.

The COVID-19 tests may help you decide how long you keep your child and your family isolated. We will discuss the results of the tests with your child's medical team to provide you with the best information about what you should do.

What if new information becomes available?
If, during the course of this study, new information becomes available suggesting that in order to give your child the best possible care for your child's condition and treatment should be changed, then we will make those changes. Changes such as these will not affect your child's participation in this study.

For peer review only - http://bmjopen.bmj.com/site/about/guidelines.xhtml

What happens at the end of the study?
Any samples that your child has provided up until the end of the study will be stored by the research team and pending ethical approval may be used in another project. If we do not make a subsequent application for ethical approval in the required timeframe or if such an application is refused, then your child's samples will be destroyed.

How will we use information about your child?
We will need to use information from your child's medical records for this research project. This information will include your child's name, medical record number and date of birth. People will use this information to do the research or to check your records to make sure that the research is being done properly. This information containing identifiers will be retained for up to 15 years.

People who do not need to know who your child is will not be able to see these personal details. Your child's data will have a code number instead. We will keep all information about your child safe and secure.

Once we have finished the study, we will keep some of the data so we can check the results. We will write our reports in a way that no-one can work out that you took part in the study.

At the end of the study the data will become "open data". This means that it will be stored in an online database so that it is publicly available. This data will be thoroughly anonymised by removing any personal information that could identify your child, such as names and addresses, before submission. This process is integral to the research process as it allows other researchers to verify results and avoid duplicating research. Data are made available on a website, free of charge, to anyone interested in the research, or who wishes to conduct their own analysis of the data. We would therefore have no control over how these data are used.

Will my child's GP be informed?
With your agreement, your child's GP will be informed should you consent to their participation in the study.

What are your choices about how your information is used?
- You can stop your child being part of the study at any time, without giving a reason, but we will keep information about your child that we already have.
- We need to manage your child's records in specific ways for the research to be reliable. This means that we won't be able to let you see or change the data we hold about your child.
- If you agree for your child to take part in this study, you will have the option to take part in future research using your data saved from this study. This data will be kept within the University of Cambridge Department of Paediatrics for up to 15 years following the conclusion of the study.

Where can you find out more about how your child's information is used?
You can find out more about how we use your information
- at www.hra.nhs.uk/information-about-patients/
- our leaflet available from **www.hra.nhs.uk/patientdataandresearch**
- by asking one of the research team

For peer review only - http://bmjopen.bmj.com/site/about/guidelines.xhtml

- by sending an email to np409@medschl.cam.ac.uk or
- by ringing us on 01223 336883

What if there is a problem?
If, when taking part in the project, your child is harmed as a consequence of either the management or conduct of the project then the NHS indemnity scheme will apply. If your child is harmed as a consequence of a problem with the design of the project, then this will be covered by insurance through the University of Cambridge's Clinical Trials policy. If your child is harmed due to someone's negligence, then you may have grounds for a legal action, but you may have to pay for it. Regardless of this, if you wish to complain, or have any concerns about any aspect of the way you have been approached or treated during the course of this study, you can contact the National Health Service Patient Advice and Liaison Service (PALS) or the Independent Complaints Advocacy Service (ICAS). Contact details are as follows:

Patient Advice and Liaison Service (PALS)
Box 53, Cambridge University Hospitals NHS Foundation Trust,
Addenbrooke's Hospital, Hills Road,
Cambridge, CB2 0QQ
Tel: 01223 216756, Fax: 01223 256170

Cambridgeshire, Norfolk and Suffolk ICAS
POhWER,
PO Box 14043
Birmingham
B6 9BL
Tel: 0300 456 2370, Fax: 0300 456 2365

What if we want to withdraw from the study?
You may withdraw your child from the study at any time without giving a reason. A decision to withdraw will not affect the standard of care you receive. In line with the guidelines from the UK Medical Research Council on the use of biological samples in scientific research, we will treat each of the blood samples which you provide us as a gift / donation. Consequently, even if you decide to withdraw from the study we may (with your permission) continue to retain any / all of the samples which you may have already provided but will discard them if you prefer.
If you wish to withdraw from the study, please let your bedside nurse know and they will get in touch with one of the research nurses or doctors.

Who has reviewed the study?
All research in the NHS is looked at by independent group of people, called a Research Ethics Committee, to protect your interests. This study has been reviewed by the research advisory committee of NHS Cambridge University Hospitals NHS Foundation Trust and the research ethics committee of Bradford-Leeds.

Thank you for considering your child's participation in this research project.

For peer review only - http://bmjopen.bmj.com/site/about/guidelines.xhtml

Department of Paediatric Intensive Care

Dr Roddy O'Donnell
Dr Rosalie Campbell
Dr David Inwald
Dr Shruti Agrawal
Dr Nazima Pathan
Dr Girish Neelegowda
Dr Riaz Kayani

Cambridge University Hospitals NHS
NHS Foundation Trust

Addenbrooke's Hospital
Hills Road
Cambridge CB2 0QQ

Switchboard: 01223 245151
www.addenbrookes.org.uk

Rapid Assay for Sick Children with Acute Lung infection Study
Consent Form – Parent/Carer of critically ill child

RASCALS

Rapid Assay for Sick Children with Acute Lung infection Study

Thank you for taking the time to consider having your child participate in 'RASCALS' – the Rapid Assay for Sick Children with Acute Lung infection Study and reading the information sheets.

If you would like your child to take part in the study, please read and sign the following forms.

We will provide you with one copy of this form and retain a second copy securely in our research department at the hospital.

Kind regards,

The Paediatric Intensive Care Unit Research Team
RASCALS researchers: Dr Nazima Pathan, Dr John Clark, Dr Iain Kean, Dr Estée Török, Prof Gordon Dougan, Prof Stephen Baker, Dr Vilas Navapurkar, Ms Esther Daubney & Ms Deborah White.
Phone: 01223 336883
Email: np409@medschl.cam.ac.uk

For peer review only - http://bmjopen.bmj.com/site/about/guidelines.xhtml

Patient identification number:		Initials
1.	I have read the information sheet on this project, dated ___/___/____ (Version________) and have been given a copy to keep. I have been able to ask questions about the project and I understand why the research is being done and any risks involved.	
2.	I give my permission for a breathing tube secretion sample, blood sample, swabs and faecal samples to be used for the purposes of this study. I understand that this blood sample did not result in any additional needle procedures for my child for the purposes of the study.	
3.	I understand that I may be asked to assist with obtaining swabs and stool specimens from my child if they are well enough to go home in the next 4 weeks if they have strongly suspected or confirmed Coronavirus disease 2019.	
4.	I understand how the samples will be collected and that giving samples for this research is voluntary. I understand I can withdraw consent for my child's participation in the study at any time, without giving a reason and without my child's medical treatment or legal rights being affected.	
5.	I understand that the samples taken could be used for other research studies, if approved by an ethics committee, for up to 10 years after the study.	
6.	I understand that the research team will email me a survey to complete via a secure online platform in 4 weeks to ask about my child's symptoms, and treatment.	
7.	I give permission for someone from the research team to look at my child's medical records to get information on my child's illness and related medical history where it is relevant to the research.	
8.	I know how to contact the research team if I need to, and how to get information about the results of the research.	
9.	I understand that any information collected about my child as part of this study will be stored securely in line with current NHS and University guidelines. I understand that only members of the clinical project team will have access to my child's identifiable information; all others analysing my data will only have access to anonymised data. I understand that my child's data will be retained for 15 years.	
10.	I understand that the information collected about my child will be used to support other research in the future, and may be shared anonymously with other academic and commercial researchers external to the project within the UK and beyond	
11.	I consent to my child taking part in this study	

For peer review only - http://bmjopen.bmj.com/site/about/guidelines.xhtml

Written consent

Carer

Name (Capitals):____

Signature:______________________________ Date: dd/mm/yyyy

Relationship to patient: ________________________________

Person obtaining consent

Name (Capitals):____

Signature:______________________________ Date: dd/mm/yyyy

Role: _____________________________

Electronic consent

Carer name (Capitals):____

Person obtaining consent

Name (Capitals):____

Signature:______________________________ Date: dd/mm/yyyy

Role: _____________________________

Verbal consent

Carer name (Capitals):____

Person obtaining consent

Name (Capitals):____

Signature:______________________________ Date: dd/mm/yyyy

Role: _____________________________

Witness to verbal consent

Name (Capitals):____

Signature:______________________________ Date: dd/mm/yyyy

Role: _____________________________

Primary investigator countersign for electronic and unwitnessed verbal consent

Dr Nazima Pathan _____________________________ Date: dd/mm/yyyy

For peer review only - http://bmjopen.bmj.com/site/about/guidelines.xhtml

For peer review only

Paediatric intensive care unit (PICU)

Division E

Policy

Non-bronchoscopic bronchoalveolar lavage sampling in children

Key messages

- Non-bronchoscopic bronchoalveolar lavage (NB-BAL) can be used to obtain lower respiratory tract secretions to assist in the diagnosis and treatment of lower respiratory tract infection
- NB-BAL samples have a higher yield than endotracheal samples alone
- NB-BAL sampling should only be undertaken after discussion with the PICU consultant that the patient is safe for the procedure
- The procedure should only be undertaken when a senior PICU nurse (in addition to the bedside nurse) and PICU doctor are available on the unit to assist if required

1 Scope

Paediatric intensive care unit – Addenbrooke's Hospital

2 Purpose

This document is to assist staff in understanding the procedure of NB-BAL sampling, and standardise the way samples are being obtained via NB-BAL in the PICU

3 Definitions

ETT: Endotracheal tube
NB-BAL: Non-bronchoscopic bronchoalveolar lavage
PICU: Paediatric intensive care unit

4 Introduction

Non-bronchosopic bronchoalveolar lavage (NB-BAL) may be also referred to as 'blind' bronchoalveolar lavage. It is a method of sampling secretions found in the lower respiratory tract, for children that are requiring invasive ventilation in intensive care for suspected or confirmed infection. Infection may be suspected based on clinical findings or chest x-ray changes.

NB-BAL has a sensitivity of up to 50-84% and specificity up to 50-95%, for detecting microorganisms in ventilator associated pneumonia.[123] There is higher

For peer review only - http://bmjopen.bmj.com/site/about/guidelines.xhtml

yield than endotracheal tube (ETT) sampling alone.[1] Advantages include reduced cost in comparison to bronchoscopic bronchoalveolar lavage, less expertise required and the ability to undertake the procedure on smaller children that may not have an airway large enough for bronchoscopy sampling to be undertaken.

Samples of lower respiratory tract secretions can assist in the management of children by way of identifying pathogens causing underlying lower respiratory tract infection. This may then allow antimicrobial therapy to be directed towards the causative bacteria, or ceased if the illness has no underlying bacterial aetiology.

Complications are uncommon in children,[1] and can be prevented or minimised with careful prior assessment of patients.

5 Responsibilities

- PICU consultant – advise whether patient is safe to undergo NB-BAL
- Senior PICU nurse and PICU doctor – maintain availability within the PICU whilst patient is undergoing NB-BAL, in case additional intervention is required
- NB-BAL proceduralist (physiotherapist/nurse/PICU doctor) – undergo procedure as per policy

6 Procedure

6.1 Indications

1. To obtain specimens for microbiology and virology that can assist in identifying pathogens causing lower respiratory tract infection

6.2 Contraindications

- FiO2 >0.85[4]
- PEEP >10cm H_2O[4]
- Pneumothorax
- Raised intracranial pressure
- Acute pulmonary oedema
- Active seizures
- Severe acute asthma
- Thrombocytopaenia[4] with platelets <50 x 10^9 L^{-1}

6.3 Precautions (discuss with PICU consultant)

- Cardiovascular instability

For peer review only - http://bmjopen.bmj.com/site/about/guidelines.xhtml

For peer review only

- Deranged clotting
- Pulmonary hypertension
- Pulmonary haemorrhage
- High ventilation pressures required

6.4 Complications

- Hypoxia[145]
- Transient reduction in tidal volume[4]
- Vagal stimulation[5]
- Pneumothorax
- Mucosal trauma
- Increased intracranial pressure
- Haemodynamic instability[5]
- Arrhythmia[3]
- Bronchospasm[3]
- Haemoptysis[6]
- Haemorrhage

6.5 Pre-procedure preparation

This procedure should only be performed where senior nursing staff and a member of medical staff are present in the PICU. These staff should be notified prior to the procedure taking place.

Consider whether additional sedation will be required for the child to facilitate the procedure. This should be discussed with nursing and medical staff.

Review the most recent chest x-ray - note the position of the tip of the ETT and distance from the carina. The suction catheter will be advanced to the level of the carina i.e. depth of ETT + distance from tip of ETT to carina

6.6 Equipment required

- T-piece anaesthetic circuit
- Bag valve mask available
- Alternative suction device eg. Yankauer suction tip available
- End-tidal CO_2, oxygen saturation and heart rate monitoring, blood pressure monitoring
- Dressing pack with sterile gloves
- Sterile suction catheter (Bicakcilar, suction catheter ideal tip, REF 190 1085 1)
- 2x Tube suction connector with female Luer-lock port (Vycon REF 801.00)
- 1x Male:Male Luer-lock connector (Vycon REF 893.00)
- Sputum trap
- 10mL, 5mL or 2mL syringes (dependent on patient size)
- 3 way tap
- 0.9% sodium chloride at room temperature

For peer review only - http://bmjopen.bmj.com/site/about/guidelines.xhtml

Paediatric intensive care unit (PICU)

Division E

6.7 NB-BAL procedure

6.7.1 Preparation

1. Discuss procedure with legal guardian/care giver where possible, and the child if requiring minimal sedation.
2. Inform nursing and medical staff plan to undertake the procedure and discuss whether additional sedation is required
3. Wash hands and put on face shield, gloves and apron, in addition to any patient specific infection control attire. Assemble equipment as shown in image 1.
4. Draw up 1ml/kg of 0.9% sodium chloride, to a maximum of 10mL in a syringe, leaving an additional 2mL air in the syringe. Connect to 3 way tap and prime suction catheter.
5. Ensure 3 way tap is open to saline flush but closed to suction system
6. Connect suction circuit, with appropriate suction pressure for age of child
7. Review observations and ventilation settings, and confirm with nursing staff safety to proceed with sedation (if indicated) and NB-BAL
8. Pre-oxygenate for 2 minutes[4] (unless there is a cardiac contraindication)
9. Auscultate lung fields

Image 1: Assembled equipment for non-bronchoscopic bronchoalveolar lavage

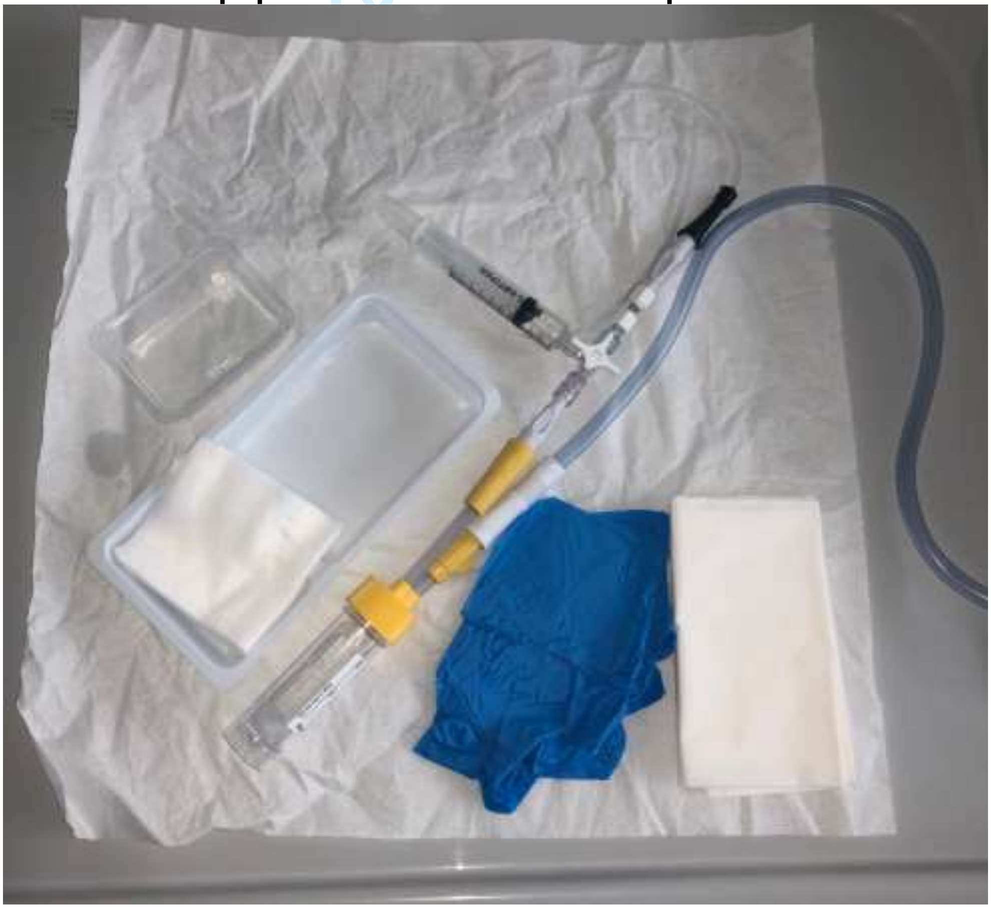

For peer review only - http://bmjopen.bmj.com/site/about/guidelines.xhtml

Paediatric intensive care unit (PICU)

Division E

6.7.2 Procedure

1. Request nursing team to administer sedation agent if this is required
2. Position child. If there is unilateral lung pathology, position the head to the contra-lateral side. i.e. If there is right side lung consolidation, position the head to the left.
3. Provide manual inflations via t-piece
4. Advance the suction catheter to the level of the carina
5. Instill saline and the 2mL of air contained in the syringe. Volume instilled is at the discretion of the proceduralist, as this may vary depending on quality of the secretions but should not exceed volume described in 'preparation'.
6. Open 3 way tap to suction, delivering suction for 2-4 seconds, withdrawing the catheter by maximum 2cm. Ensure sputum trap remains in the upright position.[7]
7. Close 3 way tap to suction and withdraw catheter

6.7.3 Post-procedure

1. Reconnect to mechanical ventilation
2. Label and send sputum sample for microbiology, cultures and sensitivities

7 Monitoring compliance with and the effectiveness of this document

Annual inspection of NB-BAL procedure whilst undertaken on PICU to review appropriateness of protocol for current equipment available on the unit and needs of staff. This will be the responsibility of PICU consultant medical staff and discussed at the following unit consultant meetings should modification of the protocol be required.

8 References

1. Khilnani, G. C. *et al.* Comparison of bronchoscopic and non-bronchoscopic techniques for diagnosis of ventilator associated pneumonia. *Indian J. Crit. Care Med.* **15**, 16–23 (2011).
2. Papazian, L. *et al.* Bronchoscopic or blind sampling techniques for the diagnosis of ventilator-associated pneumonia. *Am. J. Respir. Crit. Care Med.* **152**, 1982–1991 (1995).
3. Boots, R. J., Phillips, G. E., George, N. & Faoagali, J. L. Surveillance culture utility and safety using low-volume blind bronchoalveolar lavage in the diagnosis of ventilator-associated pneumonia. *Respirology* **13**, 87–96 (2008).
4. Bonvento, B. *et al.* Non-directed bronchial lavage is a safe method for sampling the respiratory tract in critically ill patient. *J. Intensive Care Soc.* **20**, 175114371881111 (2018).
5. Burmester, M. & Mok, Q. How safe is non-bronchoscopic bronchoalveolar lavage in critically ill mechanically ventilated children? *Intensive Care Med.* **27**, 716–721 (2001).

For peer review only - http://bmjopen.bmj.com/site/about/guidelines.xhtml

6. Gauvin, F. *et al.* Reproducibility of Blind Protected Bronchoalveolar Lavage in Mechanically Ventilated Children. *Am. J. Respir. Crit. Care Med.* **165**, 1618–1623 (2002).
7. Greater Glasgow and Clyde NHS. Bronchoalveolar (blind) lavage (PICU). *GG&C Paediatric Guidelines* (2019). Available at: http://www.clinicalguidelines.scot.nhs.uk/ggc-paediatric-guidelines/ggc-guidelines/respiratory/bronchoalveolar-blind-lavage-picu/. (Accessed: 7th November 2019)

Equality and diversity statement

This document complies with the Cambridge University Hospitals NHS Foundation Trust service equality and diversity statement.

Disclaimer

It is **your** responsibility to check against the electronic library that this printed out copy is the most recent issue of this document.

Document management

Approval:	29 November 2019		
JDTC approval:	N/A		
Owning department:	Paediatric intensive care unit		
Author(s):	John Clark		
Pharmacist:	N/A		
File name:	NB-BAL Version1 November 2019.doc		
Supersedes:	n/a (new document)		
Version number:	1	Review date:	November 2022
Local reference:		Document ID:	101409

For peer review only

For peer review only - http://bmjopen.bmj.com/site/about/guidelines.xhtml

Procedure modifications for lower respiratory lavage sampling on potential COVID-19 patients

Procedural equipment

- T-piece anaesthetic circuit
- Bag valve mask available
- Alternative suction device eg. Yankauer suction tip available
- End-tidal CO_2, oxygen saturation and heart rate monitoring, blood pressure monitoring
- Dressing pack
- In-line suction catheter (if not already connected to patient)
- 1x green tube connector for in-line suction catheter
- 1x Tube suction connector with female Luer-lock port (Vycon REF 801.00)
- 1x Male:Male Luer-lock connector (Vycon REF 893.00)
- Sputum trap
- 10mL or 20mL syringe (dependent on patient size)
- 3 way tap
- 0.9% sodium chloride at room temperature

COVID protective equipment

- Bag to dispose used equipment
- Personal protective equipment
- 2x bags for specimen

Syringe size

- If the patient is <=5kg, use a 10mL syringe
- If the patient is >5kg use a 20mL syringe

Draw up required amount of air and saline in syringe

- Saline: 1mL/kg to maximum 10mL saline
- Air:
  - If the volume of saline is <5mL: draw up 5mL air + (5 – volume of saline in mL)
  - If the volume of saline is >5mL: have 5mL air in the syringe

Procedure

- Assemble equipment, ensuring connections to sputum trap not too tight (2nd line on the connector)
- Close catheter to suction using 3-way tap
- Prime suction catheter with first 5mL of syringe contents (air/saline), holding down the button on the in-line suction catheter
- Advance suction catheter to below the tip of the ETT
- Deliver all of the remaining syringe contents (air + saline), holding down the button the in-line suction catheter
- Switch 3 way tap back to suction, hold down the button on the in-line suction catheter, ensuring the sputum trap remains upright

For peer review only

- Close the 3 way tap to suction and withdraw the suction catheter
- Turn off suction at the wall to reduce potential aerosolisation
- If patient remains stable, only 1 staff member should be in the room to complete final step if possible
- Disconnect the sputum trap and connect the in-line suction catheter back to wall suction. Place universal container lid on the specimen container.
- Dispose of used equipment in bag for highly infectious waste
- Label and package aspirated sample as required for clinical/research purposes as per current guidance

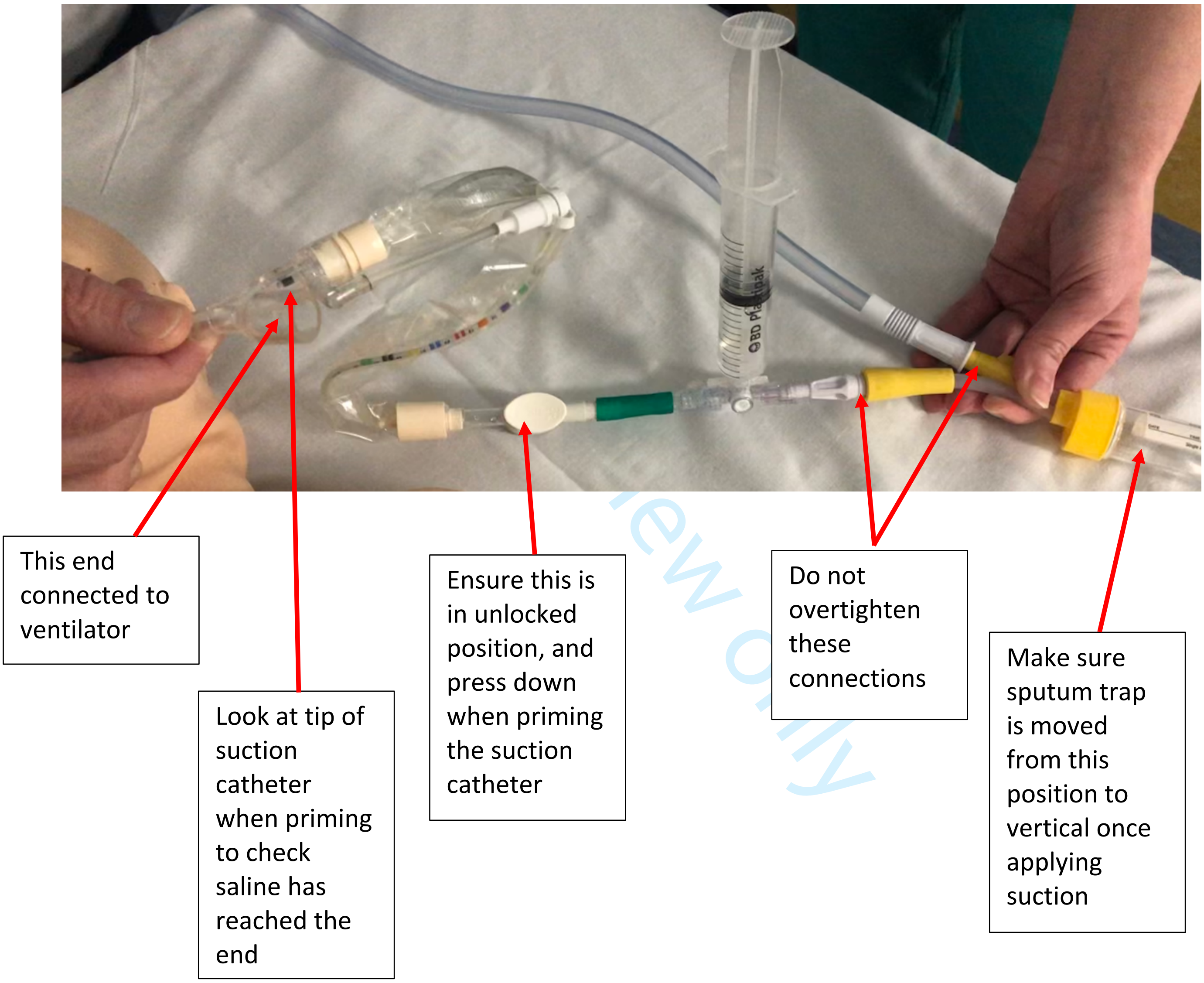

Clinical team survey

Thank you for participating in RASCALS.

Lower respiratory tract infection is a leading cause of admission to paediatric intensive care, and indication for antimicrobial therapy. It is difficult to rationalise antimicrobial therapy given these patients are so unwell, and with current microbiology techniques taking several days to provide a result. Rapid diagnostic tests have the potential to reduce the exposure of our patients to antimicrobials and provide earlier rationalisation of therapy.

We are investigating the impact of a rapid diagnostic test, a Taq-Man array, on clinical practice for ventilated children who are receiving antimicrobial treatment for lower respiratory tract infection. The following survey is to determine whether changes are implemented following Taq-man array results and would appreciate your assistance with this.

We request that you complete this survey at the time the Taq-Man array result becomes available. This survey should take less than 5 minutes. We are incredibly grateful for your time.

Kind regards,

The Paediatric Intensive Care Unit Research Team

RASCALS researchers: Dr Nazima Pathan, Dr John Clark, Dr Iain Kean, Dr Estée Török, Prof Gordon Dougan, Prof Stephen Baker, Dr Vilas Navapurkar, Ms Esther Daubney & Ms Deborah White.

Phone: 01223 336883

Email: np409@medschl.cam.ac.uk

1) Did the Taq-Man result impact on the ongoing antimicrobial type prescribed by the clinical team?
- ◯ No
- ◯ Yes antimicrobial therapy was de-escalated
- ◯ Yes, antimicrobial therapy was escalated

2) Did the Taq-Man result impact on the clinical team's decision regarding planned duration of antimicrobial therapy?
- ◯ No
- ◯ Yes, there was a plan for longer duration of antimicrobial therapy
- ◯ Yes, there was a plan for shorter duration of antimicrobial therapy
- ◯ Yes, antimicrobial therapy was ceased

3) Did the Taq-Man result contribute to a change in the patient's isolation status?
- ◯ No, the test had no impact on isolation status
- ◯ Yes, isolation was ceased due to the Taq-Man result
- ◯ Yes, isolation was continued due to the Taq-Man result

4) If you have any other comments regarding the Taq-Man result, antimicrobial prescription and isolation status please write here

____

For peer review only

Department of Paediatric Intensive Care

Dr Roddy O'Donnell
Dr Shruti Agrawal
Dr David Inwald
Dr Riaz Kayani
Dr Girish Neelegowda
Dr Nazima Pathan
Dr Stewart Reid
Dr Francesc Torres

Cambridge University Hospitals NHS
NHS Foundation Trust

Addenbrooke's Hospital
Hills Road
Cambridge CB2 0QQ

Switchboard: 01223 245151
www.addenbrookes.org.uk

Rapid Assay for Sick Children with Acute Lung infection Study
Staff focus group – experiences of TaqMan diagnostic array

RASCALS

Rapid Assay for Sick Children with Acute Lung infection Study

Thank you for taking the time to consider participating in a focus group for 'RASCALS' – the Rapid Assay for Sick Children with Acute Lung infection Study.

Before you decide to participate it is important for you to understand why the research is being done and what it will involve. Please take time to read the following information carefully. Do ask us if there is anything that is not clear or if you would like more information. Take time to decide whether or not you wish to take part.

Kind regards,
The Paediatric Intensive Care Unit Research Team

RASCALS researchers: Dr Nazima Pathan, Dr John Clark, Dr Iain Kean, Dr Estée Török, Prof Gordon Dougan, Prof Stephen Baker, Dr Vilas Navapurkar, Ms Esther Daubney & Ms Deborah White.
Phone: 01223 336883
Email: np409@medschl.cam.ac.uk

*This study has been co-sponsored by Cambridge University Hospitals NHS Foundation Trust and the University of Cambridge. Any reference to 'we' or 'us' in these documents refers to these entities.

For peer review only - http://bmjopen.bmj.com/site/about/guidelines.xhtml

Focus group participant information: Rapid Assay for Sick Children with Acute Lung infection Study

What is the purpose of this study?
The Department of Paediatric Intensive Care at Addenbrooke's Hospital is actively involved in research in children who are critically ill. We want to do research that helps us develop better ways of diagnosing and treating children admitted to our unit. Our research team would like to talk to you about the work that we are doing.

In the RASCALS project we have been researching the lung microbiome in critical care, rapid diagnostic tools for pneumonia and biomarkers of COVID-19 in children. In this focus group however, we are specifically interested in staff experiences of the TaqMan respiratory diagnostic array, which has been trialled on the unit since April 2020.

Why have I been asked to participate?
We are interested in capturing the experiences of staff currently working on the PICU in relation to the TaqMan diagnostic array. We are the first team in the world to integrate this test into a paediatric clinical service – we would like to capture this experience to drive future research and implementation of this test.

Who is organising the study?
The study is being organised by the Department of Paediatric Intensive Care by Dr Nazima Pathan and Dr John Clark, here at Addenbrooke's Hospital. This work is supervised by the University of Cambridge. No specific payments will be made to any of the staff involved for including your child in this study other than their normal salary.

Does I have to take part?
Participation in this focus group is completely voluntary.

What will happen if I take part?
A member of the research team will conduct a focus group discussion with you within a small group. This discussion will take place over a maximum of 30 minutes. This will only take place where there is sufficient staffing and time, so as to not impact on the clinical care of our patients. The research team member will provide broad signposting for areas to discuss but the discussion itself is open to the group. We are keen to obtain both positive and negative aspects of the diagnostic array and its implementation. With your permission the conversation will be recorded for coding by the research team. Your identity will be anonymised, however we would like to know your clinical role in the team to assist interpretation of our research findings.

What happens at the end of the study?
The focus group discussion will be anonymised and coded by the research team. We will use the themes and some direct quotes discussed by the focus group and publish these findings alongside our scientific findings of the RASCALS project.

What if I want to withdraw from the study?
You may withdraw your consent to participate from the project at any time. Withdrawal of consent will be a confidential process and will not fed back to staff or management within the clinical service.

For peer review only - http://bmjopen.bmj.com/site/about/guidelines.xhtml

Who has reviewed the study?
All research in the NHS is looked at by independent group of people, called a Research Ethics Committee, to protect your interests. This study has been reviewed by the research advisory committee of NHS Cambridge University Hospitals NHS Foundation Trust and the research ethics committee of Bradford-Leeds.

Thank you for considering your participation in this research project.

For peer review only

For peer review only - http://bmjopen.bmj.com/site/about/guidelines.xhtml

Department of Paediatric Intensive Care

Dr Roddy O'Donnell
Dr Shruti Agrawal
Dr David Inwald
Dr Riaz Kayani
Dr Girish Neelegowda
Dr Nazima Pathan
Dr Stewart Reid
Dr Francesc Torres

Cambridge University Hospitals NHS
NHS Foundation Trust

Addenbrooke's Hospital
Hills Road
Cambridge CB2 0QQ

Switchboard: 01223 245151
www.addenbrookes.org.uk

Rapid Assay for Sick Children with Acute Lung infection Study
Staff focus group – experiences of TaqMan diagnostic array

RASCALS

Rapid Assay for Sick Children with Acute Lung infection Study

Thank you for taking the time to consider participating in the focus group for 'RASCALS' – the Rapid Assay for Sick Children with Acute Lung infection Study and reading the information sheets.

If you would like to participate in the focus group, please read and sign the following forms.

We will provide you with one copy of this form and retain a second copy securely in our research department at the hospital.

Kind regards,

The Paediatric Intensive Care Unit Research Team

RASCALS researchers: Dr Nazima Pathan, Dr John Clark, Dr Iain Kean, Dr Estée Török, Prof Gordon Dougan, Prof Stephen Baker, Dr Vilas Navapurkar, Ms Esther Daubney & Ms Deborah White.
Phone: 01223 336883
Email: np409@medschl.cam.ac.uk

For peer review only - http://bmjopen.bmj.com/site/about/guidelines.xhtml

Participant identification number:		Initials
1.	I have read the information sheet on this project, dated ___/___/____ (Version________) and have been given a copy to keep. I have been able to ask questions about the project and I understand why the research is being done and any risks involved.	
2.	I know how to contact the research team if I need to, and how to get information about the results of the research.	
3.	I understand that the focus group interview will be recorded, and this data will be transcribed with participant identity anonymised by the research team.	
4	I understand that direct quotes from the deidentified interview transcript may be included in published research findings.	
5.	I understand that any information collected as part of this study will be stored securely in line with current NHS and University guidelines. I understand that only members of the clinical project team will have access to my identifiable information; all others analysing my data will only have access to anonymised data. I understand that my data will be retained for 15 years.	
6.	I understand that the information collected may be used to support other research in the future, and may be shared anonymously with other academic and commercial researchers external to the project within the UK and beyond	
7.	I consent to taking part in this study	

For peer review only

For peer review only - http://bmjopen.bmj.com/site/about/guidelines.xhtml

Written consent

Carer

Name (Capitals):____

Signature:______________________________ Date: dd/mm/yyyy

Relationship to patient: _________________________________

Person obtaining consent

Name (Capitals):____

Signature:______________________________ Date: dd/mm/yyyy

Role: _____________________________

Electronic consent

Carer name (Capitals):____

Person obtaining consent

Name (Capitals):____

Signature:______________________________ Date: dd/mm/yyyy

Role: _____________________________

Verbal consent

Carer name (Capitals):____

Person obtaining consent

Name (Capitals):____

Signature:______________________________ Date: dd/mm/yyyy

Role: _____________________________

Witness to verbal consent

Name (Capitals):____

Signature:______________________________ Date: dd/mm/yyyy

Role: _____________________________

Primary investigator countersign for electronic and unwitnessed verbal consent

Dr Nazima Pathan _____________________________ Date: dd/mm/yyyy

For peer review only

For peer review only - http://bmjopen.bmj.com/site/about/guidelines.xhtml
